# Effects of Twisting and Surface Finish on the Mechanical Properties of Natural Gut Harp Strings

**DOI:** 10.3390/ma16155444

**Published:** 2023-08-03

**Authors:** Nicolas Lynch-Aird, Jim Woodhouse

**Affiliations:** 1 The Old Forge, Burnt House Lane, Battisford, Suffolk IP14 2ND, UK; 2 Department of Engineering, University of Cambridge, Trumpington St., Cambridge CB2 1PZ, UK; jw12@cam.ac.uk

**Keywords:** gut, string, harp, twist ratio, twist angle, surface finish, humidity, creep, Young’s modulus, breaking strength

## Abstract

Natural gut harp strings are made from twisted bundles of gut strips, which are dried, ground, and varnished. The effects of varying the twist angle and surface finishing on the mechanical properties of gut harp strings have been explored. Strings were tested over a range of twist angles from 23.5${}^{\circ }$ to 58.3${}^{\circ }$, and with all four combinations of ground or unground and varnished or unvarnished surface finishing. The principal effects of varying the degree of twisting were that the breaking strength and tensile Young’s modulus both fell as the twist angle was increased. String makers must therefore make a compromise between sound quality and string strength and durability. Leaving the string unvarnished dramatically increased the sensitivity to changes in humidity, which, in turn, affected the thermal tuning sensitivity and creep behaviour. Grinding the string surface prior to varnishing had no significant effect on the behaviour, but did make some difference to the thermal tuning sensitivity if the string was left unvarnished. Increasing the humidity frequently triggered episodes of additional string creep. There appeared to be a threshold effect, with the additional creep triggered when the string linear density exceeded its previous maximum. When the string was not creeping, there appeared to be a strong coupling mechanism between changes in the linear density and complementary changes in the string tension, such that there was almost no net effect on the string frequency. This behaviour was independent of the twist angle and the surface finishing, suggesting that whatever the coupling mechanism was, it was not dependent on the twisted structure of the string.

## 1. Introduction

Harp players generally prefer natural gut strings over synthetic alternatives, but gut strings are highly sensitive to changes in humidity and, at the higher notes, are under sufficiently high stress that frequent breakage can be a problem. Previous studies have investigated the long-term (post-creep) mechanical properties of natural gut harp strings, and compared these to nylon and fluorocarbon synthetic harp strings [1,2]. The synthetic strings studied were either monofilaments or over-wound with a monofilament core of the same material. The gut strings, in contrast, were formed from bundles of strips of washed cow gut, which were twisted together, with no central core, prior to being dried slowly under controlled conditions, centreless ground to the required diameter, and varnished. The studies examined the stretching behaviour of the strings over three different time scales and their sensitivity to temperature changes.

The transverse resonant frequencies $f_{n}$ of an ideal fully flexible string clamped at both ends are given by the standard textbook equation [3,4]:

(1) $$f_{n}=\frac{n}{2L_{V}}\sqrt{\frac{F}{\mu }}=\frac{n}{2L_{V}}\sqrt{\frac{\sigma }{\rho }}$$

where *n* is the harmonic number, *F* is the string tension, μ is its linear density, $L_{V}$ is the vibrating length, ρ is the material bulk density, and σ=F/A is the stress, with *A* being the cross-sectional area. In practice, while Equation (1) provides a good approximation for calculating the fundamental (n=1), the bending stiffness of the string means that the actual overtone frequencies become progressively larger than the true harmonics as *n* is increased [5,6].

Natural gut and synthetic polymers are viscoelastic materials. As such, their Young’s modulus varies with frequency [7,8]. The previous studies measured the tensile tangent Young’s modulus (the stress:strain ratio for relatively small changes around the base point of interest) in three different ways: a slow measure $E_{S}$ was obtained from the gradient of the long-term stress–strain response of the string, with the data collected typically over a number of months; a medium measure $E_{T}$ was obtained by applying a tension modulation cycle with a period of about 30 min; and a fast measure $E_{B}$ was obtained via the string bending stiffness by analysing the deviation from the true harmonic ratios of the overtones in string pluck responses.

One of the most striking differences between the gut and synthetic strings, and from a musical perspective arguably the most important, was in the relationships between the faster measures of Young’s modulus and the applied stress: for both nylon and fluorocarbon strings, the faster measures of Young’s modulus, $E_{T}$ and $E_{B}$, increased fairly linearly as the applied stress was increased, and at about the same rate for both measures of Young’s modulus and both materials, strongly suggesting some common underlying mechanism, possibly corresponding to progressive straightening of the intramolecular bonds. For the gut strings, though, both the faster measures of Young’s modulus, and also the slower measure $E_{S}$, remained largely constant as the applied stress was increased, even up to the string breaking point.

For the higher strings on a harp, the $E_{B}$ values for the synthetic strings were up to three times higher than for the gut strings, which has direct implications for the degree of inharmonicity in the string overtones and their degree of damping [9], and also for the magnitude of phantom partials generated as a non-linear effect of the coupling between the longitudinal and transverse motions of the string, due to the acute angle between the string and the harp soundboard [10].

The Young’s modulus of the gut strings did appear to increase fairly linearly with the inverse of the string diameter or cross-sectional area, although preparation work for this present study suggested that this dependency was actually due to different gauge strings being twisted to different extents during manufacture.

The test rigs used for these studies had a baseboard of well-seasoned Canadian rock maple. This would be expected to have a very low coefficient of linear thermal expansion (CLTE) of 3.1–4.5 ×$10^{-6}{/}^{\circ }$C [11], less than half that of steel [12]. The longitudinal CLTE of the tested strings was typically well over an order of magnitude greater than this, and often closer to two orders of magnitude greater, so the string vibrating length $L_{V}$ could be treated as a constant, with variations in the string frequency determined primarily by changes in the string tension and linear density.

As well as measuring the thermal variation in the string frequency, the previous studies also examined the changes in the string tension and linear density. The tension behaviour was further studied by measuring the thermal variation in the medium measure of Young’s modulus $E_{T}$, which, in turn, enabled estimates of the longitudinal CLTE to be obtained for the synthetic strings.

**Figure 1:**
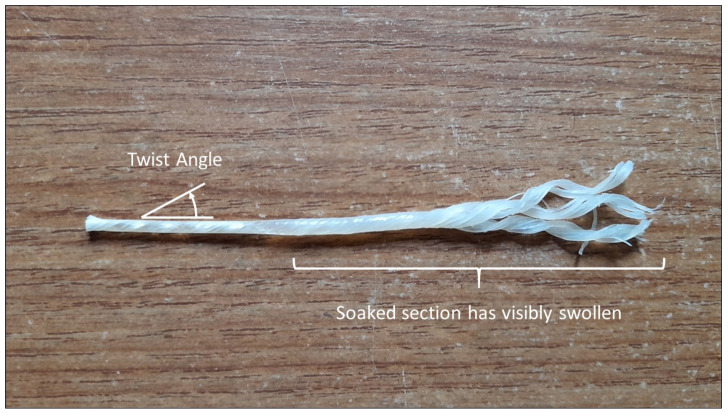
Section of gut string. The ‘twist angle’ is defined as the angular deviation of the twisted string from the untwisted case. To unravel the string, the varnish was scraped off at one end, and the string was soaked in water for a few days. The soaked section can be seen to have swollen. Reproduced from [13], with the permission of AIP Publishing.

The reasons for the sensitivity of gut instrument strings to changes in humidity are poorly understood. As the humidity changes, the gut strips within a string would be expected to absorb or release water, changing the string’s linear density. The gut strips would also be expected to swell or shrink radially. Figure 1 shows a section of gut string that was unravelled at one end by scraping off the varnish and soaking it in water for a few days. The part of the soaked section that was not unravelled can be seen to have swollen.

In the only previously published study of twist in gut strings, Bell and Firth [14] characterised the degree of twisting using the twist ratio, which is defined as the “distance along the string for one complete turn of twist (the twist-period) divided by the string diameter” [15]. A more intuitive measure, which increases the more the string is twisted, is to define the ‘twist angle’ as the angular deviation of the twisted string from the untwisted case (see Figure 1). This can be estimated as the arctangent of the ratio of the circumference of the string to the longitudinal twist period, and relates to the twist ratio via twistangle=arctan(π/twistratio).

The previous study of gut strings certainly observed humidity-related effects [1], and some initial attempts were also made to explore the sensitivity of the gut strings to changes in humidity. A rather remarkable finding was that the thermal sensitivity of the string tuning (the rate at which its fundamental frequency varied with temperature) appeared largely unaffected by changes in humidity, even when these changes led to quite noticeable changes in the string linear density. It appeared that there was a direct coupling between humidity-related changes in the linear density and complementary changes in the string tension, with the effects of these changes largely cancelling each other out.

It has been claimed that, as the gut strips swell, the twisted structure of the string leads to an increase in the string tension, and vice versa [16]. This would certainly provide a coupling mechanism between changes in the string’s linear density and corresponding changes in the tension, but any effect dependent on the twisted structure of the string should vary with the degree of twisting. This does not appear to have been tested directly until now. In the present work, the cancellation between changes in the string linear density and tension will be explored more directly, for strings with different twist angles, and with changes made to the humidity while maintaining a constant temperature.

The previous study [1] found indications that a more significant effect of changes in the humidity might be the triggering of additional episodes of string creep. In response to an increase in tension, viscoelastic materials exhibit both instantaneous, or near-instantaneous, elastic stretching and slower ‘viscous’ stretching. Both of these stretching components are reversible, but gut and synthetic polymer strings also appear to exhibit non-reversible ‘plastic’ stretching. The term string ‘creep’ is commonly used to refer to both the viscous and plastic components of string stretching, which are hard to distinguish in practice. Tests run with nylon strings to explicitly investigate the string creep behaviour [17,18,19] showed that episodes of what appeared to be plastic creep could be triggered by increasing the applied stress or the temperature beyond the previous maximum levels experienced by the strings. In both cases, it appeared that there were thresholds that had to be exceeded before the plastic creep would occur. Furthermore, there were hints that the extent of the plastic creep, triggered by an increase in the applied stress, was significantly greater if the string was already undergoing plastic creep than if the string had been allowed time to settle before increasing the stress; an observation worth further investigation.

This paper presents the results of studies investigating how aspects of the construction and finishing of natural gut harp strings affect their long-term (post-creep) mechanical properties. The effects of varying the twist angle of the string were studied, and also the effects of leaving the string unground, unvarnished, or both unground and unvarnished. In addition to the range of tests included in the previous studies [1,2], specific tests were run to investigate the effects of changing the humidity level, exploring the extent to which the string twist angle and surface finish affected sensitivity to humidity changes. Of particular interest were to investigate how changing the string construction might affect the stretching behaviour, the apparent coupling between changes in linear density and tension in response to humidity variations, and episodes of humidity-induced string creep. Some of the results from this study were previously presented in [13].

During the course of this study, it was discovered that the load cell used in the second test rig (used for testing the gut strings studied in [1]) had been insufficiently characterised, with the result that tension measurements taken at temperatures below about 20 ${}^{\circ }$C, and especially for loads above 200 N, were unreliable and frequently inaccurate. While it was possible to discard the affected results from this study, it was unfortunately too late for some of the results already presented in [1]. The consequences of these errors, and corrected results, are detailed in the main body of this paper and summarised in the Discussion and Conclusions (Section 4). The two key findings mentioned above were not affected by this discovery.

## 2. Materials and Methods

**Figure 2:**
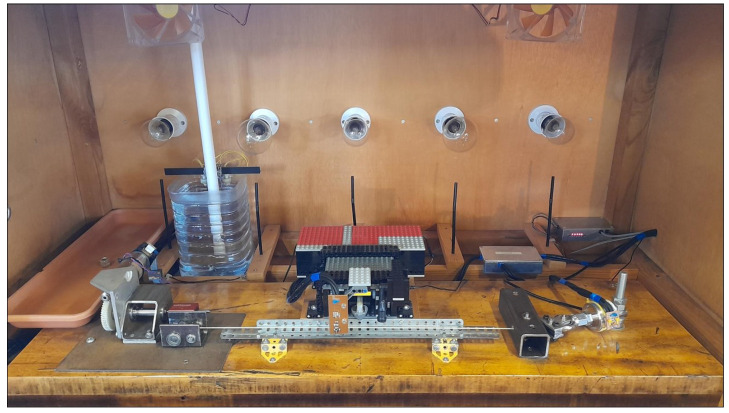
One of the two custom-built test rigs used for this study. The shallow tray on the left was added to provide a means of increasing the surface area for water evaporation, which, in turn, provided a temporary increase in the humidity level. Reproduced from [13], with the permission of AIP Publishing.

Figure 2 shows one of two test rigs used for this study. These were the same test rigs used for the previous studies, and a detailed description is given in [2]. The main test rig components were mounted on the hardwood baseboard, with a single string running along the grain of the baseboard and about 2 cm above it. The string passed over two bridge pins, one horizontal and one vertical. The bridge pins were mounted in ball bearings, and were set perpendicular to each other to minimise the effect that the direction of plucking would have on the vibrating length of the string [20]. A motorised string winder was placed at one end, seen together with the horizontal bridge pin on the left in Figure 2. The drive motor was equipped with an integral shaft rotation sensor which, coupled with two stages of worm gear reduction, enabled string tuning length adjustments to be made with an accuracy of better than ±0.008 mm. The other end of the string was connected to a load cell (Novatech F256EFR0KN 40 kg). This had a digital thermometer strapped to it and, after thermal characterisation, could be used to measure string tensions over a range of 0–300 N, accurate to within ±0.2 N.

The string was plucked at its mid-point to minimise the generation of the second and other even harmonics, and hence assist in the automated identification of the string fundamental. The string plucker was built from Lego and used a plastic guitar plectrum to pluck the string. A microphone to record the pluck responses was mounted on the plucker assembly. The temperature and relative humidity (RH) close to the string were measured using a digital temperature and humidity sensor, also mounted on the plucker assembly. The original sensors (Sensirion SHT75) had a nominal accuracy of ±0.3 ${}^{\circ }$C and ±1.8% RH. One of these failed during the study, so the sensors in both test rigs were replaced with updated versions (Sensirion SHT85) with improved nominal accuracy of ±0.1 ${}^{\circ }$C and ±1.5% RH.

The whole test rig was contained in a wooden chamber with a removable Perspex front panel. Heating was provided using combinations of 100 W and 150 W incandescent light bulbs, and a water reservoir was included to maintain the humidity levels within the chamber. Fans were provided for air circulation, and a metal heat distribution bar, made from Meccano, ran behind and below the string to help even out the temperature along the string. Black plastic drinking straws below the light bulbs acted as light pipes, leading to sensors monitoring the light bulbs. For this study, a shallow tray, approximately 42 cm long by 13 cm wide, was added on the left side of each test chamber. Adding water to the tray increased the surface area for evaporation, which, in turn, temporarily increased the humidity within the test chamber until the water in the tray had evaporated.

The Novatech load cells used in the test rigs were constructed mainly from steel. As the temperature of these load cells varied, both the gain and zero offset changed. The gain of the electronics (located outside the test chamber) used to measure the load cell output was also sensitive to temperature changes. Both the load cell and the associated electronics were equipped with digital thermometers to monitor their temperatures, and had to undergo a thermal characterisation procedure to enable suitable temperature compensation functions to be devised. As mentioned in the Introduction (Section 1), it was discovered during the course of this study (during the first part examining the effects of varying the string twist angle) that the thermal characterisation of the Novatech load cell in one of the test rigs (Rig 2), and its associated electronics, was insufficient. The original characterisation was run during the summer months when the temperatures rarely fell much below 20 ${}^{\circ }$C. The derived temperature compensation functions did not extend well to lower temperatures, with the result that tension readings taken at lower temperatures, especially for loads above 200 N, were unreliable and often inaccurate. Having discovered this issue, the load cell and associated electronics were re-characterised during the winter months, with the load cell electronics (situated outside the test chamber) subjected to temperatures across a range of 8–33 ${}^{\circ }$C, and the load cell itself subjected to temperatures across a range of 6–44 ${}^{\circ }$C. The opportunity was also taken to check the performance of the load cell in Rig 1, for which no further characterisation was found to be required. The full characterisation reports are included in the data archive [21].

Direct measurements could therefore be made of the string fundamental frequency (via FFT of the recorded pluck response), the string tension, and any tuning length adjustments (the lengths of string wound onto or off the winding shaft), together with the temperature and relative humidity close to the string. The string diameter was measured at each target fundamental frequency using the same manual micrometer screw gauge used for the initial string measurements (see Table 1), with a resolution of 0.01 mm. The string linear density was obtained from the measured frequency and tension using Equation (1), and combined with the measured diameter to give the bulk density.

### 2.1. Strings Tested

The gut strings used in this study all came from Bowbrand (Norfolk, UK) [22] with the exception of one string, designated ST1, which was from Real Guts Strings (Manchester, UK) [23]. Bowbrand makes its strings from strips of washed cow gut. Bundles of gut strips are treated in a chemical bath, which includes a fixative, and then twisted together on a motorised twisting bench. The number of strips in each bundle varies both with the required string gauge and the herd from which the gut has been sourced. The chosen bundle counts are only a rough guide, however, as the final string gauge may differ from the intended diameter due to variations in the thickness of the individual gut strips. The twisted bundles are dried slowly under controlled conditions, centreless ground to give a smooth surface, and coated with several coats of clear or coloured varnish. The gut strings from Real Guts Strings, in contrast, are generally intended for use on bowed instruments rather than harps and are made from sheep gut. They are twisted by hand and often have a more complex construction than just a single twisted bundle, to enable higher twist angles to be obtained, and are neither ground nor varnished, although they are polished to achieve a smoother finish.

The previous studies [1,2] used a number of standard production strings with a range of diameters. For the investigation into the effects of varying the string twist angle, Bowbrand manufactured a bespoke set of strings: four bundles of strips of washed cow gut, all taken from the same batch from the supplying abattoir, were prepared up to the point where they were ready for spinning. The bundles were numbered T1 to T4, and each bundle was cut in half prior to spinning, with the two halves of each bundle labelled A and B, hence, T1A, T1B, T2A, T2B, etc. The string bundles are normally twisted three times during the manufacturing process: the bulk of the twisting is applied when the string bundles are still wet from the treatment tanks, and a smaller number of additional turns are applied at two points during the drying process. The initial spinning is done on a motorised bed, while the additional turns are applied manually using a geared spinner. For these bespoke strings, a different number of initial turns was applied to each half of the same original bundle. Four different turn counts were used: one being the most that one of the bundles (T1A) would tolerate before breaking; one being the minimum thought to be sufficient to avoid the string falling apart; and two intermediates, one of which corresponded to the normal count for this type of string bundle. All eight half-bundles were given the usual additional number of turns during the drying process. Once the strings had been dried, they were centreless ground, reducing their diameter by about 0.15 mm in each case, and then clear varnished in the normal way.

To study the effects of varying the string surface finish, a set of six strings, designated S1, S11, S2, S12, S3, and S5, were selected which had all received the same number of twists and which had been treated, wound, and dried in the normal way. Each string was cut into two sections, one of which was centreless ground in the normal way, while the other section was left unground. The two sections from all but one of the strings (S5) were then clear varnished. The strings were chosen so that the ground diameter of all but the thinnest string (S3) would match, approximately, the unground diameter of one or more other strings in the set. The ground (‘polished’) and unground sections were labelled P and U, respectively, hence, S11U, S11P, S12U, S12P, etc., with S11P and S12U, for example, having approximately the same diameter (see Table 1). The sheep gut string, ST1, which had a much higher twist angle than any of the cow gut strings, was included in the surface finish tests for comparison.

Table 1 lists the strings, with their unstretched diameters and bulk densities, which were measured prior to testing. String identification numbers are shown in the first column of Table 1. These string identification numbers include a suffix letter to differentiate between sections of the same string, since most of the strings were long enough to provide multiple sections for testing, and are used consistently in the summary data sets accompanying this paper and in the much larger comprehensive data archive [21]. The string diameters were measured using a manual micrometer screw gauge with a resolution of 0.01 mm. Multiple measurements were taken along the length of each string and averaged to give the values shown in Table 1.

While measuring the diameters of the unground Bowbrand strings, it was noted that the roughness of the string surface made it impossible to obtain accurate measurements of the average diameter. This was especially the case for string S2U, which had a very rough surface. On the basis that the string density could be expected to be the same for the ground and unground sections of the same string (with perhaps a small difference due to the layer of varnish, which has a typical density of 930 kg/m${}^{3}$), a diameter correction estimate was obtained for each of the unground string sections. These corrections are included in the values shown in Table 1. With the exception of string S2U, which, as noted earlier, had a very rough surface (and which, consequently, was not included in the tests), the diameter corrections for the unground varnished Bowbrand strings were reassuringly consistent at about −0.04 mm. The diameter correction for the unground and unvarnished Bowbrand string (S5U) was larger at about −0.07 mm, but this was probably to be expected on the basis that the varnishing would have resulted in a smoother surface finish. Examination of the string from Real Guts Strings (string ST1) showed that, despite being unground and unvarnished, it had a surprisingly smooth surface finish. In the absence of any equivalent ground string, it was not possible to estimate a diameter correction for this string, but the surface finish appeared smooth enough that any diameter correction was probably not required.

It might be expected that the string diameter should increase with the twist angle. This was the case for the two halves of strings T3 and T4, but not for strings T1 and T2, presumably due to diameter variations along the original bundles of gut strips. Consequently, the string diameter has not been used in this study as an explanatory variable or as the abscissa in any plots.

In general the twist angle values shown in Table 1 were calculated using the diameter of the finished string. In the case of the strings with one half ground and the other half left unground, the twist angle was taken to be the same for both halves and was calculated using the diameter of the ground half. No special allowance was made for whether or not the string had been varnished.

Each string section was tested over a range of different target fundamental frequencies, listed in Table 1. Where possible the same set of four frequencies were used for each string: 174, 235, 288, and 324 Hz. The string sections identified as ST1a and ST1b were actually the same piece of string. The string proved to be much weaker than the cow gut strings, and failed very early in its original test sequence after just one set of tests with a target fundamental frequency of 174 Hz. Fortunately, the string broke at the load cell clamping point and could be remounted onto the test rig. A set of tests was then run using a set of lower target fundamental frequencies. Different identification numbers were assigned to avoid confusion in the resulting data files and processing.

The string sections have been listed in Table 1 in the order in which they were tested. Where possible, complementary pairs of strings were tested alongside each other (one in each test rig) to control for environmental changes due to the time of year and prevailing weather conditions. For the first three string sections, however, the results from the tests in Rig 2 had to be discarded when it was found that the Rig 2 load cell had been insufficiently characterised. And for the next four string sections (T4Ac to T3Ba), only one test rig and load cell combination (Rig 2 with the load cell from Rig 1) was available while waiting for the re-characterisation of the Rig 2 load cell to be completed (which required the use of the Rig 1 chamber). Some string sections broke before the tests at the full set of target fundamental frequencies could be completed: T4Bb, T2Bb, ST1a, S11Pa, and ST1b. In these cases, the test sequence in the other test rig was usually halted at the same point. This was the case for string sections T2Ba and S11Ua. The testing of string section T2Ab was halted when it was found that problems incurred during the diagnosis of the failed SHT75 temperature and humidity sensor had led to excessive stretching of the string. The full set of tests spanned a period of more than four years, albeit with an eight-month break during the COVID-19 pandemic while waiting for the strings for the surface finish study to become available.

### 2.2. Test Protocols

The test methods used for measuring the string behaviour were the same as those employed in the previous studies [1,2], except that additional humidity-variation tests were run specifically to explore the string behaviour in response to humidity changes (see below), and some minor changes were made to some of the data processing techniques, as detailed in Section 2.3. In addition to the tests run in the custom-built test rigs described above, a number of breaking strength tests were run using a variety of methods and test machines (see below).

For each of the string sections listed in Table 1, a sequence of tests was run at each of the listed target fundamental test frequencies. A creep-settling test was run first, with the test rig operating in its constant-frequency mode, until the string appeared to have settled, as determined using the creep-monitoring approach described in [2]. For the first part of this test, the string target frequency was increased by 6% (about one semitone) for a few days to accelerate the creep process and significantly reduce the settling time [2]. A Young’s modulus $E_{T}$ test was run, using a 30-min cycle of tension modulation around a base tension, measured when the string was tuned to the target frequency at the base temperature of $T_{0}=20{\;}^{\circ }$C. At the end of the Young’s modulus test, the test chamber was opened, the string diameter was re-measured, and a series of manual pluck responses were recorded. Tests were also run with the string held at constant length (no tuning adjustments) and at constant tension. These latter tests were used to measure the string’s thermal tuning sensitivity and the accompanying changes in the string tension and linear density under these two conditions. It was found that a satisfactory alternative to running separate tests with the string held at constant tension was to use the data recorded at the base tension points during the Young’s modulus $E_{T}$ tests. Consequently, the constant-tension tests were only run for some of the strings.

During all of these tests, a 24 h heating cycle was applied, to synchronise with the daily cycle in ambient temperature. Care had to be taken not to overheat the strings, so the temperature inside the test chambers was kept below about 35 ${}^{\circ }$C. By fitting lines to the measured test responses, it was possible to obtain values for the string frequency, tension, linear density, and Young’s modulus $E_{T}$, at the chosen base temperature $T_{0}$ of 20 ${}^{\circ }$C, together with the thermal sensitivities of each of these parameters around $T_{0}$. The exact test sequence at each target fundamental test frequency was occasionally varied to accommodate periods of being away. As with the previous studies, each string was typically mounted on its test rig for several months.

The fast measure of Young’s modulus $E_{B}$ was derived from the string bending stiffness, which, in turn, was obtained by analysing the deviation of the string overtones from an exact harmonic series [20]. The test rig control program was able to identify and analyse the overtone frequencies obtained from the automated string plucks, to provide estimated values for $E_{B}$, both at the base temperature $T_{0}$ and as the temperature was varied [2]. This approach worked well for synthetic strings, but gave poor results with gut strings. For this study, the values of $E_{B}$ were therefore obtained by analysing the manual pluck responses, which were recorded using the wire-break method [20] to pluck the string close to one end. Using the manual pluck responses did not, however, provide a means to estimate the thermal sensitivity in $E_{B}$.

Ambient humidity levels can vary both with and relative to the temperature. As the temperature increases, the absolute humidity will usually increase with it, while the relative humidity falls. At other times, however, the humidity may change independently of the temperature, with both the absolute and relative humidity levels changing in the same direction. The primary water reservoir in each test chamber generally maintained the humidity at around 60% RH, when the temperature inside the chamber was 20 ${}^{\circ }$C, although this could still be affected to some extent by changes in the ambient humidity levels outside the chambers, which changed with the weather.

To examine the effects of humidity changes more specifically, an additional isothermal humidity-variation test was run at the end of the main test sequence for a number of the strings. For these tests, the test rig was first run in its constant-frequency mode, with the usual 24 h heating cycle, until the string had resettled. When the temperature next reached 25 ${}^{\circ }$C, the test rig was switched to its constant-length (no adjustments) mode, and the temperature was maintained at 25 ± 2 ${}^{\circ }$C. After a further settling period of a day or so, water was added to the shallow tray, to a depth of a few mm. This caused a sharp increase in the humidity levels inside the chamber, which persisted for a few days until the water in the tray had evaporated. The string frequency and tension were recorded every 5 min until the water in the tray had evaporated, the humidity had returned to (or close to) its initial level, and the string had settled again. This cycle of humidity variation could then be repeated. The requirement to run these tests at constant temperature, and the absence of active cooling in the test rigs, meant that these tests could only be run during the colder parts of the year, as indicated in Table 1.

It is well established, for synthetic polymers at least, that the breaking strength varies with the strain rate: reducing the strain rate lowers the breaking strength (see, for example, [24]). Intuitively, this makes sense: at lower strain rates, there will be more time for string creep to occur, weakening the string. The situation for natural polymers is less clear, but a fairly recent study of muscle fibre bundles [25] also found that the breaking strength fell as the strain rate was reduced. The strings selected for this present study were mostly too thick to be able to break them on the custom-built test rigs used for the bulk of the testing (Figure 2), but it was possible to run breaking strength tests on some thinner sections of gut strings left over from the previous study. For these tests, the length adjustment (strain) was increased in 1 mm increments at intervals of 1, 10, 100, and 1000 s, corresponding to strain rates between $2\times 10^{-6}$ /s and 0.001 /s. The temperature inside the test chamber was maintained at 20 ± 2 ${}^{\circ }$C during these tests.

**Figure 3:**
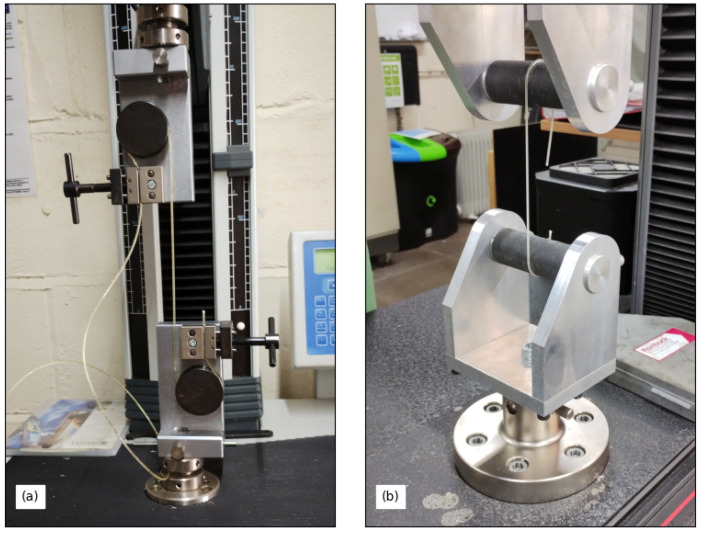
The string mounting arrangements for the breaking tests run at (**a**) Bowbrand, using a Lloyd Instruments LRX Plus series Materials Testing Machine, and (**b**) the University of Cambridge Department of Engineering, using an Instron 5985.

The breaking strengths of the finished strings manufactured with different twist angles (T1A, etc.) were measured using Bowbrand’s own string strength tester (a Lloyd Instruments LRX Plus series Materials Testing Machine fitted with 01/4622 Bollard Grips). At each end, a section of string was passed around a circular bollard, 4 cm in diameter, and then into a parallel-plate clamp, forming an arrangement similar to an elongated S or butcher’s hook (Figure 3a). The strings were stretched at a constant rate of 100 mm/min, corresponding to a strain rate of approximately 0.007/s, and the tests were conducted at ambient room temperature.

COVID-19 restrictions made it impractical to travel to Bowbrand to measure the breaking strengths of the set of strings with different surface finishes at the time of their manufacture. Instead, after the other tests for this study had been completed, and the COVID-19 restrictions had been lifted, a series of breaking strength tests were run in the University of Cambridge Department of Engineering, using an Instron 5985 fitted with a 10 kN load cell. The string mounts consisted of horizontal shafts with a rough emery-coated surface and a clamp on the back side (Figure 3b). The gauge length, taken as the distance between the centres of the string mount shafts, was set to 120 mm for all the tests. Tests were run on sections of all the strings except for T4A, of which there was none left. Most of the tests were run at a stretching rate of 3 mm/min, but a small number of additional tests, with sections of strings S1P and T3A, were run at faster and slower rates, ranging from 0.1 to 30 mm/min. These tests were also run at ambient room temperature.

All but two of the tests were run with new sections of string that had not been previously stretched, and with the string mounted as shown in Figure 3b. The exceptions were the tests with sections of string ST1. There was only a short (approx. 20 cm) section of new string available, and this was mounted directly between the clamps on the backs of the mounts, rather than around the mount shafts. Even if the gauge length had been shortened, passing the string around the mount shafts would have left a very short test length, and it was not thought the moment exerted through the mounts would make any significant difference. To supplement this test, a piece of the previously tested string ST1a/b was also tested.

### 2.3. Data Processing

The raw data files obtained from the test rigs were processed in the same way as described in detail in [2], but with a few refinements. For the previous studies [1,2], the data processing was conducted rather manually using Excel spreadsheets supplemented by a small number of specially written programs, particularly for generating the $E_{T}$ values. For this study, the analysis procedure was sufficiently well established that Python scripts [26,27,28] could be produced, which considerably automated the data analysis. These scripts are included in the data archive [21]. In addition, a few changes were made to the way the test data were analysed, as described in the following subsections.

#### 2.3.1. Length Adjustment Correction Offset

When a string was first mounted on its test rig, it was hard to define accurately the zero point of length adjustment, because of the details of string take-up onto the winding shaft. As a result, the raw measurements of length adjustment had an unknown offset. The approach taken in the previous studies, to estimate the required length adjustment correction, was to fit a quadratic to the length adjustment and tension values recorded at the start of the Young’s modulus $E_{T}$ tests, for the first three target fundamental frequencies, and extrapolate the fitted curve back to zero tension.

For this study a simpler approach was taken: a line was fitted to the first two points of the tension versus length adjustment response recorded at the start of the first constant-frequency creep-settling test, when the string had just been mounted on the test rig and was being tuned for the first time. The required correction offset was estimated by extrapolating the fitted line back to zero tension. Using this simpler approach resulted in a far lower spread in the long-term stress–strain responses compared to the previous technique, especially for sections taken from the same string, and had the added advantage that the correction offset could be estimated from the very first measured test response.

This method of estimating the length adjustment offset correction is still not perfect: there will still be some string creep not accounted for, such that the first tension value used to estimate the stiffness gradient may be smaller than it should, and the estimated correction offset may be too small. It seems likely that this approach will also work better at low stress levels, with correspondingly slow creep rates.

#### 2.3.2. Filtering of $E_{T}$ Values

The study of nylon strings [2] found what appeared to be a friction effect in Rig 1, which caused jumps in the length adjustment response during the tension modulation cycle for the Young’s modulus $E_{T}$ tests. This behaviour persisted despite changing the ball bearings used to mount the vertical bridge pin at the end of the string connected to the load cell. To get around this, values for $E_{T}$ were calculated from the tension and length adjustment changes for each step of the modulation cycle, and then filtered using a program that compared the ratio of the total length adjustment for each tension step to the first length adjustment for that step. Steps where the adjustment ratio fell outside upper and lower bounds were excluded. For this study, values of $E_{T}$ were again calculated for each step of the tension modulation cycle, but a simpler filtering approach was used, which just applied upper and lower limits to these $E_{T}$ values. This simpler filtering approach gave very similar results to the previous approach.

#### 2.3.3. Loss Factor Plots

The manual pluck responses used to calculate values for the fast measure of Young’s modulus $E_{B}$ were also processed to produce plots of the damping loss factor [29,30] for each overtone, plotted against the overtone frequency. To achieve this, a spectrogram of each pluck response was first calculated and scanned to identify the overtones. For each overtone *n*, the precise frequency $\omega_{n}$ (rad/s) was calculated by fitting a line to the phase versus time response for the corresponding frequency bin, while the decay rate $d_{n}$ (dB/s) was calculated by fitting a line to the power spectral density versus time response for that same frequency bin. The corresponding loss factor $\eta_{n}$ could then be calculated as:

(2) $$\eta_{n}=-\frac{d_{n}}{10\log_{10}\left(e\right)\;\omega_{n}}=-0.23\frac{d_{n}}{\omega_{n}}$$

## 3. Results

### 3.1. Stretching and Bending Behaviour

#### 3.1.1. Bulk Density

**Figure 4:**
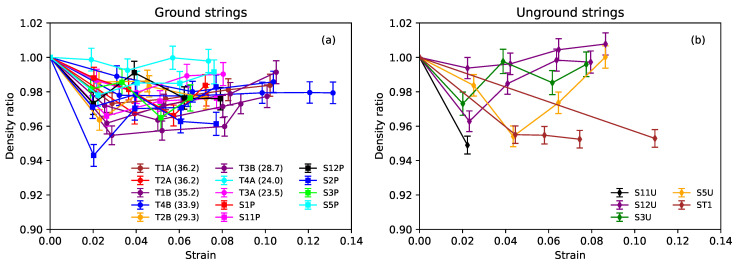
The ratio of the bulk density to that of the unstretched string, plotted against strain for (**a**) the ground strings and (**b**) the unground strings. The apparent rise in the density of the unground strings from Bowbrand was probably a consequence of the diameter adjustment being applied (see Section 2.1) rather than a real effect.

Figure 4a shows the ratio of the bulk density of each of the ground strings to the unstretched density, at the base temperature of $T_{0}=20$${}^{\circ }$C, plotted against the strain. These strings exhibited the same behaviour as seen before (Figure 5c in [1]), with the bulk density falling initially, and quite quickly, before settling out at between 97% and 98% of the unstretched value. There did not appear to be any dependency on the string twist angle, nor on whether or not the string was varnished. The error bars show the range of variation that could be attributed to the limited accuracy of the string diameter measurements.

Figure 4b shows the results obtained from the unground strings. On the face of it, the bulk density of the unground strings from Bowbrand appeared to fall initially, in the usual manner, and then rise as the strain increased, in some cases finishing up at higher density levels than before any stretching was applied. Examination of the linear density and cross-sectional area ratios for these strings (again referred to the unstretched values) revealed that the difference was due to the cross-sectional area ratio falling at a faster rate than for the equivalent ground strings, while the linear density ratios fell at similar rates. It seems that a more likely explanation for this difference in behaviour is that the diameter adjustments applied to the unground strings (Section 2.1) should have been progressively reduced as the strings stretched. Tellingly, the behaviour of string ST1, for which no diameter adjustments were made, exhibited the same form of behaviour as the ground strings.

Assuming the density behaviour of the unground strings matched that of the ground strings, the required reduction in the diameter adjustments would only amount to around a 3% or 4% increase in the cross-sectional area (at full strain), so the effect on the derived stress values used in the plots below could be safely ignored.

#### 3.1.2. Breaking Stress

**Figure 5:**
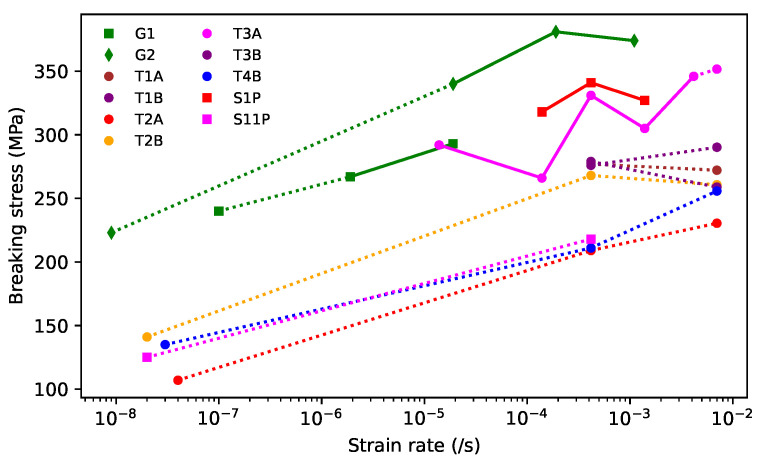
Breaking stress results obtained using the various test approaches, plotted against the strain rate. Solid lines link results obtained using the same test method, while dotted lines link results from different test methods.

Figure 5 shows the breaking stress results obtained using the various test approaches described in Section 2.2, plotted against the strain rate. The thinner sections of gut strings, left over from the previous study [1], were designated G1 (diameter 0.64 mm) and G2 (diameter 0.84 mm). Solid lines link results obtained using the same test method, while dotted lines link results from different test methods. On the right side of the plot, the dotted lines connect results from the tests run at Bowbrand and at Cambridge University. For strings G1, G2, T2A, T2B, T4B, and S11P, additional points, shown on the left of the plot, were obtained from the breaking tensions of sections of the same strings, which broke while undergoing the normal test sequence as described in Section 2.2, with the strain rate estimated from the total test duration [21]. The different test approaches provided results over a range of strain rates spanning nearly six decades.

While there was quite a considerable variation in the breaking strengths of different sections of the same string, the overall trend seems clear: as expected, reducing the strain rate resulted in lower breaking stress levels. From the discussions to come below, it might have been expected that G1, being thinner, should have been less twisted and stronger than G2. In fact, string G1 had proven to be consistently weaker than expected, to the extent that it provided no useable results during the previous study, as the different string sections tested consistently broke before a set of tests could be completed.

The breaking stress results reported here, and presumably also in Bell and Firth’s study [14], were referred to the unstretched string diameters and made no allowance for the strings getting thinner as they were stretched. Looking at the diameter variations of the strings tested in this study, and the strings stretched to breaking in the previous study [1], suggested that the breaking stress values referred to the actual string diameter would be around 10% to 15% higher. This does not affect the overall finding though: the breaking stress fell as the strain rate was reduced.

**Figure 6:**
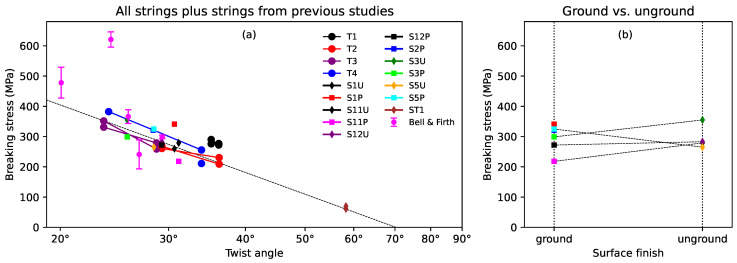
Breaking stress results plotted against (**a**) twist angle on a logarithmic scale and (**b**) surface finish. The solid lines connect the results from the two halves of each of T1, T2, T3, and T4. The black dashed line in (**a**) was fitted to all the Instron test results except those for strings T1A, T1B, and ST1, while those in (**b**) connect the results from the ground and unground halves of the same string. Both subplots use the legend given in (**a**).

Figure 6a shows the breaking stress results for the various strings, plotted against the twist angle. The solid lines connect the results from the two halves of each of T1, T2, T3, and T4. For the tests run using the Instron tensile tester at Cambridge University, only the results for the tests run at 3 mm/min are shown. These results clearly showed that, as would be expected, increasing the twist angle reduced the breaking stress of the string. Figure 6b compares the results from the ground and unground Bowbrand strings provided for the surface finish study. The dashed lines connect the results from the ground and unground halves of the same string. There was no clear dependency on the surface finish, either for ground versus unground or varnished versus unvarnished strings. The corresponding breaking strains, across all the tested strings, were typically between 0.15 and 0.28, and showed no obvious dependency on the strain rate, twist angle, or surface finish of the string.

The dashed line in Figure 6a was fitted to the results obtained using the Instron tensile tester at Cambridge University, with a strain rate of 3 mm/min, for all the tested strings except the two halves of T1 (which often seemed to be something of an outlier), and ST1. A logarithmic axis was used for the twist angle since this showed a better straight-line trend than using a linear axis, and resulted in the fitted line passing very neatly through the results for string ST1, despite those data not being included in the fit. However, rather than crossing the zero-stress axis at a twist angle of 90${}^{\circ }$, as might be expected, the fitted line reached zero stress at about 70${}^{\circ }$. As mentioned earlier, the breaking stress values were referred to the unstretched string diameters. Including the reduction in diameter as the strings were stretched would certainly raise the breaking stress values and lift the fitted line. However, given that the strings’ breaking strains and their density (Figure 4), and hence volume, behaviour appeared largely independent of the twist angle, it would seem likely that correcting for the diameter reduction would increase the breaking stress values by a fairly consistent percentage, largely independent of the twist angle. This would have the effect of scaling the *y*-axis of Figure 6a, but would otherwise leave the plot unchanged.

At lower twist angles, the trend suggested by the fitted line in Figure 6a clearly cannot be extrapolated back to a twist angle of 0${}^{\circ }$, on a logarithmic axis, to provide an estimate of the maximum breaking stress for an untwisted string; instead, the linear trend must break down at some point.

The results from Bell and Firth’s study [14] are included in Figure 6a for comparison. Bell and Firth tested multiple strings at each diameter. The vertical bars span ±1 standard deviation about the mean breaking stress for each string diameter. While there is a much greater spread in their results, the same general trend seems to apply, with the breaking strength falling as the twist angle was increased. Bell and Firth did not provide breaking strain data, but their breaking stress and Young’s modulus results might suggest breaking strains in the range of 0.11 to 0.20. These are probably over-estimates, however, assuming the stress–strain responses for their strings displayed the same pattern of behaviour seen during the breaking strength tests run for the present study (see below). The strings used by Bell and Firth, which were provided by Salvi Harps, were also made from cow gut, but would not have been manufactured by Bowbrand. Bowbrand was still using sheep gut for their harp strings at that time, and only later switched to using cow gut. Bell and Firth plotted their results against the string diameter, and found that the breaking stress and Young’s modulus (see Section 3.1.3) both fell as the diameter increased. The twist angle generally increased with the string diameter, and it would seem that this is the more likely explanatory parameter.

Bell and Firth ran their tests using a Monsanto Tensometer and stated that their “tests were conducted with the tension increasing at a rate of, at most, 50 N per minute” [14]. This suggests that the different diameter strings may have been tested at different strain rates, possibly of around $10^{-4}$/s, which may have accounted for some of the variation in their breaking stress results included in Figure 6a.

#### 3.1.3. Young’s Modulus

As mentioned above, the slow measures of Young’s modulus $E_{S}$ were obtained from the gradients of the long-term stress–strain responses. At each point, the string had been given the time required to finish creeping and settle at the target fundamental frequency. All the long-term stress–strain responses showed a high degree of linearity, similar to those seen before for gut strings (Figure 1c in [1]), corresponding to almost constant values for the slow measure of Young’s modulus $E_{S}$.

Indeed, all three measures of Young’s modulus remained largely constant as the applied stress was increased, similar to the behaviour reported in the previous study (Figure 2c in [1]). With very few exceptions, the variation in Young’s modulus with the applied stress was less than 0.5 GPa, for all three measures across all the strings tested for the present study.

In contrast to the long-term stress–strain responses used to generate the slow measures of Young’s modulus $E_{S}$, the stress–strain responses during the breaking strength tests typically rose at a faster rate initially, up to a strain of around 0.025, before settling into a slower but largely linear rise. Fitting lines to these stress–strain slopes from the results obtained using the Instron tensile tester at Cambridge University, with a strain rate of 3 mm/min, provided a further measure of the tensile Young’s modulus, designated here as $E_{X}$.

**Figure 7:**
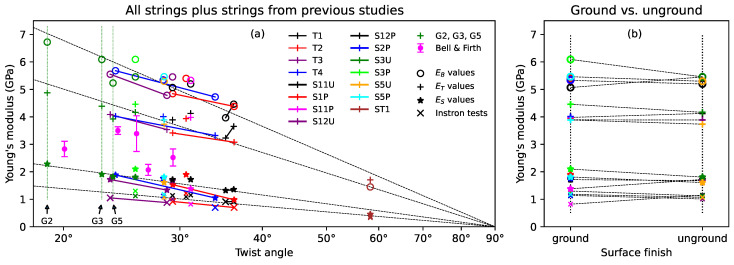
The average Young’s modulus at 20 ${}^{\circ }$C, plotted against (**a**) twist angle on a logarithmic scale and (**b**) surface finish. Results are shown for the four measurement approaches: $E_{B}$ (o) from bending stiffness, $E_{T}$ (+) from modulation of string tension, $E_{S}$ (*) from the slope of the long-term stress–strain responses, and $E_{X}$ (×) obtained from the Instron breaking tests run at 3 mm/min. Subplot (**a**) includes the values for strings G2, G3, G5 from the previous study [1], as well as the results from Bell and Firth’s study [14]. The black dashed lines in (**a**) were fitted to all the results shown except for those from Bell and Firth and string ST1, and were constrained to pass through the point (90${}^{\circ }$, 0), while those in (**b**) connect the results from the ground and unground halves of the same string. Both subplots use the legend given in (**a**).

Figure 7a shows the mean of each of the measures of Young’s modulus $E_{S}$, $E_{T}$, $E_{B}$ at 20 ${}^{\circ }$C for each string, together with the additional measure $E_{X}$, plotted against the twist angle, on a logarithmic scale. The solid lines connect the results from the two halves of each of T1, T2, T3, and T4. The results for strings G2, G3, and G5 from the previous study [1], as well as the results from Bell and Firth’s study [14], are also shown. For all four measures of Young’s modulus, and across all the tested strings, there was a clear dependency on the twist angle, with the Young’s modulus falling as the twist angle was increased. This has implications for damping behaviour and sound quality: increasing the twist angle will reduce the inharmonicity associated with bending stiffness and also reduce the high-frequency damping (leading to a brighter sound), but at the cost of making the string weaker. The reduction in inharmonicity, and perhaps in damping, may explain why these high-twist strings are often favoured by bowed-instrument players.

The Young’s modulus $E_{X}$ results obtained from the Instron tests, and the results from the study made by Bell and Firth, present something of a puzzle: both sets of results were obtained in a fairly similar manner, and yet the results from Bell and Firth lay between the $E_{S}$ and $E_{T}$ values obtained in this study, while the $E_{X}$ values were consistently lower than the $E_{S}$ values. Since the $E_{X}$ values were obtained from sections of the same strings as the $E_{S}$, $E_{T}$, and $E_{B}$ values, these should arguably take primacy here. Recall the observations on plastic string creep from the Introduction (Section 1): for the $E_{S}$ values, each time the target frequency, and hence the applied stress, was increased beyond its previous maximum by a significant amount (overcoming any threshold effect) the string was allowed time to finish creeping. In contrast, during the breaking strength tests, the applied stress was steadily increasing throughout the test, potentially resulting in more extensive plastic creep than for the $E_{S}$ values, as seen with the nylon strings, and hence in a lower measure of the tensile Young’s modulus. Exactly the same arguments should, however, also apply to the tests run by Bell and Firth, leaving the puzzle unresolved. Perhaps the explanation lies in some difference in the details of the string manufacturing approach and the chemical treatments used, or some subtlety of the testing or the way in which Bell and Firth extracted their measures of Young’s modulus from their stress–strain data.

It would be expected that all four measures of Young’s modulus should fall to zero as the twist angle approaches 90${}^{\circ }$. Figure 7a was plotted using a logarithmic scale for the twist angle. The dashed black lines were fitted to all the results shown, except for those from Bell and Firth and string ST1, and were constrained to pass through the point (90${}^{\circ }$, 0). There was no particular theoretical basis for choosing a logarithmic scale, but the fitted lines do appear to match the measured data rather well, including the results from string ST1.

Figure 7b compares the results for the ground and unground Bowbrand strings provided for the surface finish study. The dashed lines connect the results from the ground and unground halves of the same string. There was no clear dependency on the surface finish, either for ground versus unground or varnished versus unvarnished strings, across all four measures of Young’s modulus, though it was curious that the spread in values was generally lower for the unground strings.

It is perhaps worth noting that the long-term strain values of both natural gut and synthetic strings are likely to be affected by the maximum temperature and, for gut strings, humidity levels experienced during settling and testing; so, in considering the $E_{S}$ results, knowledge of the test environmental parameters, as described in Section 2.2, is important.

#### 3.1.4. Loss Factor Plots

Loss factor plots were produced in an attempt to explore whether the variations in the string construction might have more subtle effects on how they sounded. In general, the effects on the damping behaviour were small and not very clear. Varying the string twist angle did not have any discernible effect beyond that due to the variation in Young’s modulus.

**Figure 8:**
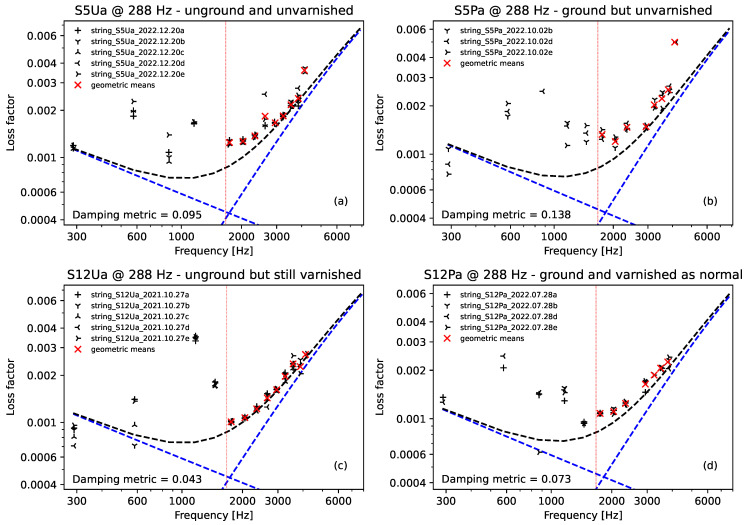
Loss factor plots derived from wire-break pluck responses recorded from strings with different surface finishes: (**a**) S5Ua, unground and unvarnished; (**b**) S5Pa, ground but unvarnished; (**c**) S12Ua, unground but varnished; (**d**) S12Pa, ground and varnished as normal. These strings were all tested in the same test rig: Rig 2.

Figure 8 shows loss factor plots derived from wire-break pluck responses recorded from the two halves of two of the strings, covering the various surface finish combinations: S12P, which was ground and varnished as normal; S12U, which was unground but still varnished; S5P which was ground but not varnished; and S5U, which was neither ground nor varnished. The dashed lines show the expected damping loss variation, dominated by air damping at low frequencies and by internal damping at high frequencies [9,30]. The blue dashed lines show the expected losses due to these two damping components separately, while the black dashed lines show the expected total damping loss. These losses vary with the string stiffness, tuning, density, and diameter. For this set of strings, these parameters were all the same or fairly similar, so the expected loss factor curves are almost identical. The plotted points show the actual loss factors at the different overtones extracted from the wire-break pluck responses, as described in Section 2.3.3. Especially at higher frequencies, the measurements follow the predicted pattern very well.

The plots shown in Figure 8 were all derived from pluck responses recorded from strings tested on Rig 2. At frequencies below around 1600 Hz, additional damping came into effect, associated with vibration modes within the test rig. These additional effects were significantly more disruptive for pluck responses recorded using Rig 1, to the extent that the corresponding loss factor plots were largely useless. Pluck responses recorded for strings tuned to the lower target fundamental frequencies, at and below 235 Hz, also gave unsatisfactory results: the strings were not taut enough to provide sufficiently well-defined decay responses at the higher overtone frequencies required.

To aid the comparison of the behaviour shown in the loss factor plots, a damping metric was calculated as the root mean square (RMS) of the base 10 logarithms of the ratios between the actual and expected loss factor values for all the plotted points above a threshold frequency of 1670 Hz, or, in more visual terms, the RMS distance between the actual and expected values on the logarithmic scale used in Figure 8. The threshold of 1670 Hz was chosen as it was approximately midway between 5×324 and 6×288 Hz. In practice, any loss factor plot yielding a damping metric value much above 0.1 was too messy for any clear interpretation.

Comparing the plots shown in Figure 8 and their associated damping metrics (shown in the bottom left corner of each subplot), the ground strings appeared to exhibit a slight increase in their high-frequency damping, while the varnished strings, perhaps somewhat counter-intuitively, appeared to exhibit slightly lower damping. These effects are, however, very small and may well be inaudible in practice. It should also be borne in mind that this is only one set of plots from a fairly limited data set. Limiting the analysis to pluck responses recorded from strings tested on Rig 2 at 288 Hz or above meant that only 10 sets of pluck responses from five string sections (those shown plus S2Pa, which exhibited similar behaviour to S12Pa) were really worth considering. The full set of results is included in the data archive [21].

### 3.2. Response to Temperature Changes

#### 3.2.1. Thermal Tuning Sensitivity

**Figure 9:**
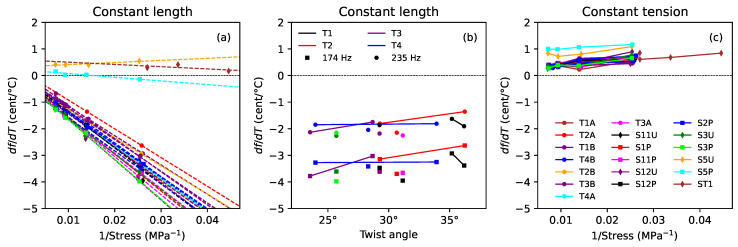
The thermal tuning sensitivity df/dT in $¢{/}^{\circ }$C for (**a**,**b**) strings held at constant length and (**c**) strings held at constant tension, plotted against (**a**,**c**) the inverse of the applied stress and (**b**) the twist angle. The dashed lines shown in (**a**) were fitted to the results for each string separately. (**a**,**c**) use the legend given in (**c**). The individual points plotted in (**b**), for the surface study strings, use the same colours as (**a**,**c**), but with the marker shapes defined in (**b**) for the different test frequencies.

Figure 9 shows the thermal tuning sensitivity for the strings held at constant length, with no tuning adjustments (Figure 9a,b), and at constant tension (Figure 9c). Tuning deviations are conveniently expressed in cents (¢), there being 1200 ¢ in an octave [3]:

(3) $$\delta f_{¢}=1200\log_{2}\left(\frac{f}{f_{0}}\right)\;¢$$

where *f* and $f_{0}$ are the actual and desired frequencies, respectively. The thermal tuning sensitivities shown in Figure 9 are given in $¢{/}^{\circ }$C. As was found previously, for both natural gut and synthetic polymer strings [1], the tuning sensitivity of the strings held at constant length appeared to vary quite linearly with the inverse of the applied stress, especially for the varnished strings. The dashed lines shown in Figure 9a were plotted using a separate linear regression analysis for each string, with the inverse of the applied stress as the explanatory variable. The fits for the varnished strings were all excellent with $r^{2}$ values of 0.988 or greater. As also found previously, the varnished gut strings all went flat as the temperature was increased.

The most noticeable feature in Figure 9a is that the three unvarnished strings behaved very differently from the varnished strings, with much smaller thermal tuning sensitivities, which varied only slightly with the applied stress. The two unground and unvarnished strings, S5U and ST1, both went sharp as the temperature increased, and showed fairly similar levels of sensitivity despite their very different make-up and construction. Unlike the varnished strings, grinding did seem to make a difference for the unvarnished strings, with the ground string S5P showing almost no tuning variation with temperature.

Looking at the spread in the responses for the varnished strings, there was no apparent difference between the ground and unground strings, but it appeared there might be some variation with the twist angle. This is explored in Figure 9b, which plots the tuning sensitivity values obtained at the first two target fundamental frequencies (174 and 235 Hz), since these gave the greatest degree of vertical separation in the plot, against the twist angle. The solid lines connect the results for the two halves of each of T1, T2, T3, and T4. These results might suggest that increasing the twist angle made the strings slightly less sensitive to temperature changes, but the evidence is far from compelling, and any benefit that might be gained would be small.

It is generally accepted that the minimum perceptible change in pitch of a musical note is around 3 ¢, though this varies with the pitch and complexity of the sound [31]. Hence, for the varnished strings, even modest temperature changes could be expected to make a noticeable difference to the string pitch, an effect that is all too well known to harp players.

Figure 9c shows that holding the varnished strings at constant tension significantly reduced their thermal sensitivity: the strings now went sharp instead of flat, and at a much reduced rate of around 0.5 $¢{/}^{\circ }$C, with very little variation with the applied stress. There was now no noticeable variation with the twist angle or between the ground and unground strings. For the unvarnished strings, in contrast, being held at constant tension made very little difference for the unground strings S5U and ST1, but significantly degraded the tuning stability of the ground string S5P, which now went sharp at about 1 $¢{/}^{\circ }$C.

**Figure 10:**
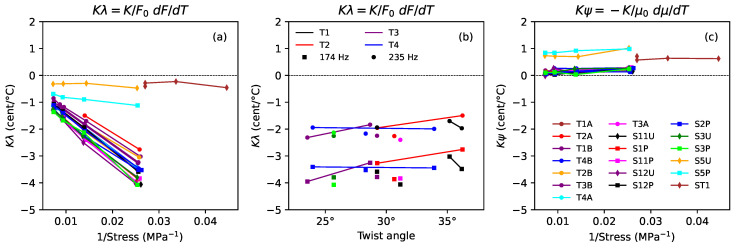
The thermal sensitivity for strings held at constant length in (**a**,**b**) the string tension and (**c**) the string linear density, plotted against (**a**,**c**) the inverse of the applied stress and (**b**) the twist angle. (**a**,**c**) use the legend given in (**c**). The individual points plotted in (**b**), for the surface study strings, use the same colours as (**a**,**c**), but with the marker shapes defined in (**b**) for the different test frequencies.

These constant-tension results, for the varnished strings, mark the first and most significant departure from the results presented in the previous study [1]. It is now clear that the results shown in Figure 9c in [1], and the related results in Figures 10c and 10f in [1], were badly affected by the insufficient initial characterisation of the load cell used for those tests; those earlier results were invalid and should be ignored.

#### 3.2.2. Thermal Variations in Tension and Linear Density

From Equations (3) and (4) in [1], the thermal tuning sensitivity (measured in $¢{/}^{\circ }$C), around a base temperature $T_{0}$, for a string held at constant length with no tuning adjustments, would be expected to be:

(4) $${\left.\frac{df_{¢}}{dT}\right|}_{L}\approx K\left(\lambda +\psi \right)\;¢{/}^{\circ }C$$

where λ and ψ represent the normalised thermal variations in the string tension and linear density:

(5) $$\lambda =\frac{1}{F_{0}}\frac{dF}{dT}\;\;\mathrm{and}\;\;\psi =-\frac{1}{\mu_{0}}\frac{d\mu }{dT},$$

$K=600/\ln\left(2\right)$ and $F_{0}$ and $\mu_{0}$ are, respectively, the string tension and linear density at $T_{0}$. For this study, the base temperature used was $T_{0}=20$ ${}^{\circ }$C.

Figure 10 shows these two components, Kλ and Kψ, for the strings held at constant length, plotted on the same scales as used in Figure 9. Adding the values shown in Figure 10a to those shown in Figure 10c gives the results shown in Figure 9a. Comparing Figure 10a and Figure 10c, it can be seen that, for the varnished strings, the thermal tuning sensitivity was almost entirely governed by the thermal variations in the string tension, with very little contribution from changes in the string linear density. Comparing Figure 10b and Figure 9b, which are almost identical, the variations with twist angle also came from changes in the string tension. It would appear that the layer of varnish was effective at protecting the varnished strings from the changes in humidity which normally accompany changes in temperature.

For the unvarnished strings, the story was quite different, with similarly sized contributions from changes in both the string tension and linear density. The two components, Kλ and Kψ, showed little variation with the applied stress but had opposite signs, and the overall thermal tuning sensitivity of the strings was determined by the balance between them. The unvarnished strings also showed an interesting mix of similarities and differences in their behaviour: the thermal tension sensitivities of the unground strings S5U and ST1 were similar, despite the differences in their make-up and construction, while those of the ground string S5P were significantly different. In contrast, the thermal variations in the linear densities of strings S5U and S5P were almost the same, while those of string ST1 were somewhat different.

Holding the strings at constant tension did not completely remove the effects of temperature changes: as the temperature changed small lengths of string had to be wound on or off the winding pin to maintain the required tension, which, in turn, affected the string linear density and hence its tuning. Consequently, while Figure 9c and Figure 10c are similar, they are not identical. In particular, the small variations with the applied stress, shown in Figure 9c, can now be seen to be due to residual tension effects, rather than changes in the string linear density.

#### 3.2.3. Tension Components

The analysis given in [2] showed that, for synthetic polymer strings, the thermal variation in the string tension would be expected to depend on thermal changes in the Young’s modulus and thermal expansion–contraction effects governed by the longitudinal CLTE α. The previous study for gut strings [1] found that there appeared to be some sort of coupling between humidity-related changes in the linear density and complementary changes in the string tension, with the effects of these changes largely cancelling each other out. This suggests that Equation (16) from [2] should be extended to include an additional term in ψ such that:

(6) $$\lambda =\frac{1}{F_{0}}\frac{dF}{dT}=\frac{1}{A_{0}E_{0}}\frac{d(AE)}{dT}-\frac{A_{0}E_{0}\alpha }{F_{0}}-C\psi$$

where *C* is a weighting for the linked contribution from ψ, and quantities with the subscript 0 refer to values at the base temperature $T_{0}$. The observed behaviour suggests that *C* should perhaps be close to one. The measure of Young’s modulus *E*, which should be appropriate to the time scale of the temperature modulation, was, for these studies, most closely approximated by the values $E_{T}$ obtained through the tension modulation tests. This was also the only measure of Young’s modulus for which it was possible to measure the thermal variation. These measurements are more accurately described as measures of $\left(1/A_{0}\right)\;d\left(AE\right)/dT$, rather than dE/dT, since it was not possible to measure the thermal variation in the string diameter.

**Figure 11:**
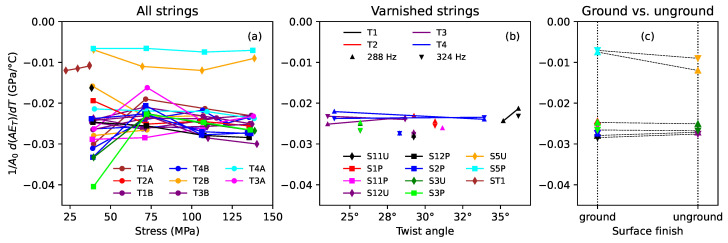
Thermal variation in Young’s modulus $E_{T}$ plotted against (**a**) the applied stress, (**b**) the string twist angle, and (**c**) the surface finish. (**a**) uses the legends given in (**a**) and the lower half of (**b**). The individual points plotted in (**b**,**c**), for the surface study strings, use the colours defined in the lower half of (**b**), but with the marker shapes defined in the upper half of (**b**) for the different test frequencies.

Figure 11 shows the results obtained for the thermal variation in the Young’s modulus $E_{T}$. With the exception of some of the measurements taken at the lower stress levels, the thermal sensitivity of the Young’s modulus generally remained fairly constant as the applied stress was increased, for all the strings tested. This agrees with the findings of the previous study [1], which in this case appear not to have been unduly affected by the problems with the load cell characterisation, perhaps because the measurements involved relatively small and localised tension changes, as opposed to measuring or maintaining absolute tension values across a wide temperature range.

For Figure 11b and Figure 11c, rather than trying to take averages across some or all of the applied stress levels, the results from the tests run at target fundamental frequencies of 288 and 324 Hz are shown. The varnished strings all displayed quite similar levels of thermal sensitivity, and there was no apparent dependency on the string twist angle or on whether the string was ground or unground.

The Young’s modulus of the unvarnished strings appeared to be much less sensitive to temperature changes than the varnished strings. The results for the two unground strings, S5U and ST1, appeared similar, suggesting that there was again no dependency on the twist angle. Possibly there was some difference between the ground and unground strings in this subset, but the results were not clear.

Rearranging Equation (6), the longitudinal CLTE can be estimated as:

(7) $$\alpha =\frac{F_{0}}{A_{0}^{2}E_{0}^{2}}\frac{d(AE)}{dT}-\frac{1}{A_{0}E_{0}}\frac{dF}{dT}-C\frac{F_{0}}{A_{0}E_{0}}\psi$$

**Figure 12:**
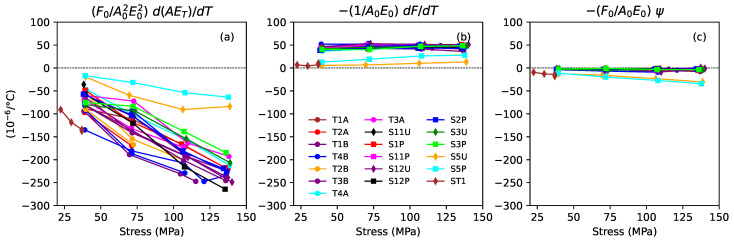
(**a**–**c**) The three component terms from the right-hand side of Equation (7) for the longitudinal CLTE, plotted against the applied stress. The weighting factor *C* for the term in ψ was set to 1 in this case. All three subplots use the legend given in (**b**).

Figure 12 plots the three component terms from the right-hand side of Equation (7) against the applied stress for all the strings tested. The weighting factor *C* for the term in ψ was set to 1 for this purpose. For the varnished strings, pretty much all the variation was due to the first term. The other two terms were almost the same across all the varnished strings and showed practically no variation with the applied stress. More significantly, the term in ψ was relatively very small, at only about $-2.5\times 10^{-6}{/}^{\circ }C$, and could probably be safely ignored for realistic values of the weighting factor *C*.

For the unvarnished strings, the term in ψ cannot be so easily ignored. If the weighting factor C≈1, then it may be that the contributions from the second and third terms on the right-hand side of Equation (7) would largely cancel each other out, with the CLTE being determined mainly by the first term, but this is largely speculation.

**Figure 13:**
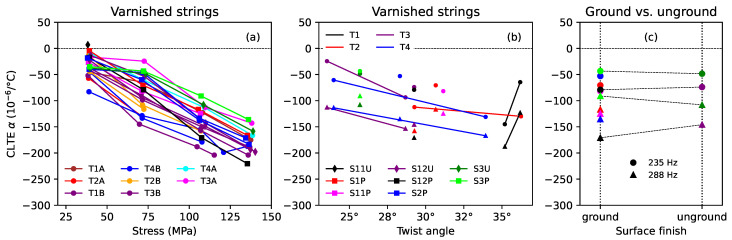
Estimated coefficient of linear thermal expansion (CLTE) for the varnished strings plotted against (**a**) the applied stress, (**b**) the string twist angle, and (**c**) the surface finish. (**a**) uses the legends given in (**a**) and the lower half of (**b**). The individual points plotted in (**b**,**c**), for the surface study strings, use the colours defined in the lower half of (**b**), but with the marker shapes defined in (**c**) for the different test frequencies.

Based on the above findings, the CLTE for the varnished gut strings could be estimated fairly confidently in the same way as done previously for the synthetic strings [1], using Equation (7) without the term in ψ. Figure 13 shows the estimated CLTE responses obtained in this way. Figure 13b and Figure 13c show the values obtained at the second and third target fundamental frequencies (235 and 288 Hz). The CLTE responses for these gut strings were quite different from those obtained previously for nylon and fluorocarbon strings, which either remained fairly constant or became smaller in magnitude (i.e., less negative) as the applied stress was increased [1]. Since the CLTE behaviour was determined primarily by the first term on the right-hand side of Equation (7), it could readily be explained: the CLTE for all the varnished strings fell fairly linearly as the stress increased due to the $F_{0}$ term; the spread in the CLTE responses was due to the variation with twist angle, which, in turn, was due to the $1/E_{0}^{2}$ term; and none of the component terms exhibited any dependence on whether the string was ground or unground.

### 3.3. Response to Humidity Changes

**Figure 14:**
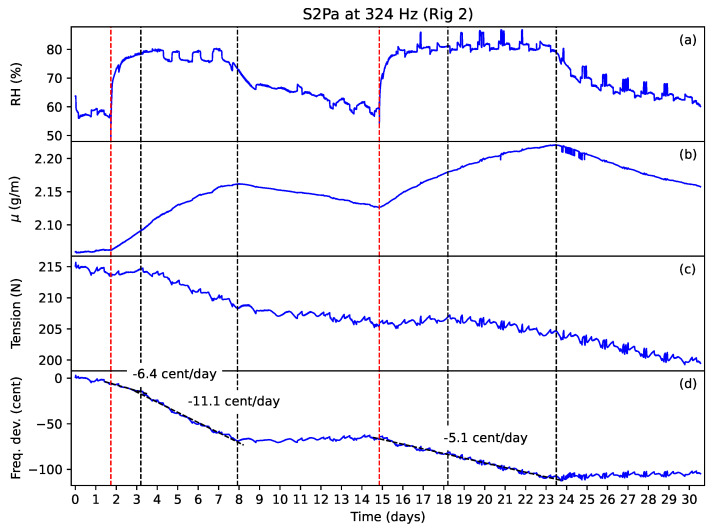
Humidity-variation test results for string S2Pa: (**a**) relative humidity (RH), (**b**) string linear density μ, (**c**) string tension, and (**d**) frequency deviation, all plotted against time. The dashed red lines mark the times when water was added to the shallow tray to boost the humidity level inside the test chamber, while the dashed black lines mark various transition points.

Figure 14 shows the results from the humidity-variation tests run on string S2Pa, which was ground and varnished as normal. Similar plots for other ground and varnished strings covering a range of twist angles have been previously presented in [13]: T3A (23.5${}^{\circ }$), T3B (28.7${}^{\circ }$), and T1A (36.2${}^{\circ }$). These and the results of the humidity-variation tests run on other strings are included in the data archive [21].

The red dashed lines mark the times when water was added to the shallow tray, while the black dashed lines mark particular transitions and other points of interest. In every case, adding water to the tray caused the humidity levels in the test chamber to rise sharply, remaining elevated until the water in the tray had evaporated, before falling back towards the original levels. Typically, the relative humidity, shown in subplot (a), was boosted by about 20%, from 55–60% to 75–80%. The string linear density, shown in subplot (b), rose and fell with the humidity, but with slower response times. The ripples in the various plots correspond to temperature variations around the target temperature of 25 ${}^{\circ }$C. The linear density was affected less than the string tension by these temperature variations simply because it was slower to respond to the corresponding changes in relative humidity.

For the varnished strings like this one, each time water was added to the shallow tray, the string tension, shown in subplot (c), would typically initially rise with the linear density. Then, at some point, the gradient in the tension response would change, with the tension usually now falling, or sometimes remaining more or less constant, while the linear density continued to rise. Sometimes the tension would start to fall almost immediately after the water was added to the tray. On the second or subsequent times water was added to the tray, the gradient change in the tension response often appeared to coincide with the string linear density exceeding its previous maximum, suggesting that there was some sort of threshold effect involved.

Each of these transitions in the tension response was usually accompanied by a corresponding change in the frequency response, shown in subplot (d): if the frequency was fairly stable, it started to fall; if the frequency was already falling, the rate at which it was falling increased. If the linear density did not exceed its previous maximum, then usually the string tension just rose and fell with the linear density, with little or no net effect on the string frequency. The periods where the string linear density was rising and the string tension was falling appeared to mark clear periods of additional string creep. There were other periods, however, where the string appeared to be creeping as soon as water was added to the shallow tray, and the relationship between the changes in the string linear density and tension was less obvious.

The other key transition points occurred when the shallow tray dried out and the string linear density started to fall. The string tension continued to fall, often at much the same rate as before the tray dried out. The string frequency either stopped falling, marking an end to the period of apparent creep, or if it continued to fall it did so at a much lower rate before stopping some time later.

Confirmation that the strings had in fact been creeping was obtained by comparing the string linear density and tension at the start and end of each test: generally, the linear density could be seen to be returning towards its original level, while the string tension, and frequency, finished up significantly lower than at the start of the test. The responses for string T1Aa [13] gave a particularly clear example: over the course of the full test duration of nearly 65 days, the string tension fell by nearly 7%, and the string frequency fell by about 3%, while the linear density fell by less than 1%. Clearly, the string had experienced an internal relaxation over the course of the test.

**Figure 15:**
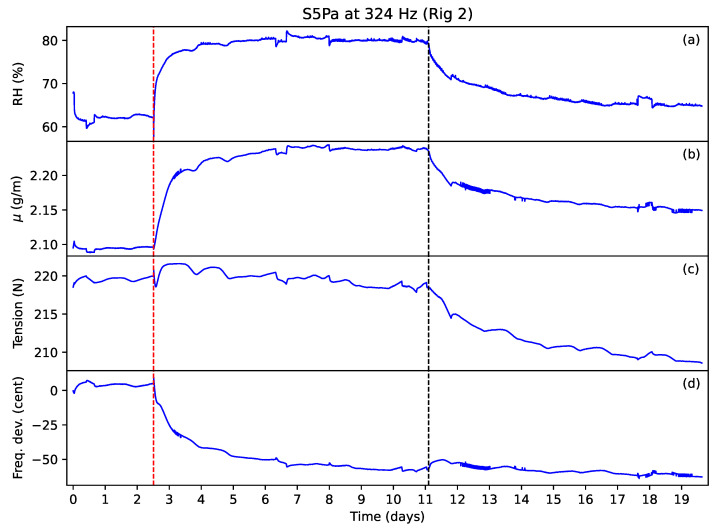
Humidity-variation test results for string S5Pa: (**a**) relative humidity (RH), (**b**) string linear density μ, (**c**) string tension, and (**d**) frequency deviation, all plotted against time. The dashed red line marks the time when water was added to the shallow tray to boost the humidity level inside the test chamber, while the dashed black line marks the transition point when the tray had dried out.

Figure 15 shows the results from string S5Pa, which was ground but not varnished. The unvarnished strings responded much more quickly to the humidity changes. The string linear density responses practically matched the relative humidity responses, even tracking the ripples due to temperature variations, and appeared to reach similar saturation levels as for the varnished strings. The string tension appeared to start falling almost as soon as water had been added to the shallow tray, and again this period of rising linear density and falling tension appeared to correspond to a period of string creep. For these unvarnished strings, however, the string frequency fell much faster initially before levelling out in a fairly exponential manner.

One of the most significant aspects of the string behaviour, for both the varnished and unvarnished strings, is that there were significant periods where there were large changes in the string tension and linear density, after the shallow tray had dried out, where the effects of these changes appeared to cancel out such that the string frequency remained almost constant. This coupling effect between the string linear density and tension did not appear to be dependent on the string twist angle in any way.

The strings were not always induced to start creeping: string S12Ub seemed largely unaffected by the period of elevated humidity. Conversely, string T3Bb was induced to creep to such an extent that it spontaneously broke; the results for this string are included in [13].

Many of the periods of creep in the varnished strings were sufficiently steady that creep rate gradients (¢/day) could be obtained from fitted lines. Within this limited data set, comparing the creep rates obtained from strings T3Aa (23.5${}^{\circ }$) and T3Bb (28.7${}^{\circ }$), and from strings T1Ba (35.2${}^{\circ }$) and T1Aa (36.2${}^{\circ }$): the creep rates appeared to increase with the string twist angle. This would seem to be consistent with the variation in the slow measure of Young’s modulus $E_{S}$ with the twist angle. Possibly, the interfaces between the different strips of gut making up the string were altered by the degree of twist, affecting the propensity to creep.

It also appeared that the unground strings crept slightly faster than the ground strings; this certainly appeared to be the case for the unvarnished strings S5Ua and S5Pa. Comparing the initial fast creep rates for two of the pairs of strings tested at the same times: string S3Ua (25.7${}^{\circ }$) crept at a rate of −12.6¢/day, while string S2Pa (28.3${}^{\circ }$) crept at a rate of −11.1¢/day, and string S12Ua (29.2${}^{\circ }$) crept at a rate of −10.1¢/day, while string S1Pa (30.7${}^{\circ }$) crept at a rate of −9.5¢/day. These differences in creep rate appeared to overcome any effect from the differences in the string twist angles. Apart from this slight difference in creep rate, there did not appear to be any difference in the behaviour of ground and unground strings.

Comparing the different creep rates for those strings where water was added on multiple occasions, the creep rate appeared to be slowing down over time, reminiscent of the possible exponential decay behaviour seen for the unvarnished strings. Other tests made by the authors, with both nylon and gut strings, have shown that threshold effects, similar to that seen here for the string linear density, also occur for the triggering of string creep by increases in the longitudinal stress or temperature. In these cases, the effects on the required string length adjustments (to maintain a constant stress or frequency) were also observed to follow an exponential trend towards a new limiting value.

## 4. Discussion and Conclusions

This study has explored the effects of varying the twist angle and surface finishing on the mechanical properties of natural gut harp strings. Strings were tested over a range of twist angles from 23.5${}^{\circ }$ to 58.3${}^{\circ }$, and with all four combinations of ground or unground and varnished or unvarnished surface finishing. The stretching behaviour was assessed through three different measures of the Young’s modulus, with very different test time scales: on the order of weeks, minutes, and milliseconds. The breaking behaviour was investigated through a number of different strength tests, with strain rates spanning nearly six decades. The resulting stress–strain data also provided a fourth measure of the Young’s modulus. Loss factor plots were produced from string pluck responses in an attempt to explore whether the variations in the string construction might have more subtle effects on how they sounded. The sensitivity of the strings to environmental changes was explored through a combination of temperature-variation tests and isothermal humidity-variation tests, with the latter providing some key insights into the string creep behaviour.

### 4.1. Twist Angle Effects

The string twist angle has been defined as the angular deviation of the twisted string from the untwisted case, which can be estimated as the arctangent of the ratio of the circumference of the string to the longitudinal twist period.

The most significant effect of varying the string twist angle was that both the breaking strength and all four measures of the Young’s modulus fell steadily as the twist angle was increased. Reducing the Young’s modulus, and hence bending stiffness, of the string will result in lower inharmonicity and less high-frequency damping of the string overtones. Gut string makers must therefore make a compromise between the sound quality of the string and its strength. The tensile stress of the strings on a harp increases along the string scale, with the top strings operating close to their breaking points [9,32]. At the same time, the number of audible overtones falls; the top note on a concert harp is G7 on the piano scale (3136 Hz). A sensible compromise, therefore, would be to wind the upper strings as little as possible, to maximise their strength and durability, and to wind the lower strings with a relatively high twist angle to improve the sound quality. Not surprisingly, harp string makers appear to do exactly that, presumably as a result of hard-earned experience through trial and error.

The breaking stress and Young’s modulus results from the string ST1 from Real Guts Strings followed the same trends with twist angle as the Bowbrand strings. In fact, across all the tests, there was no obvious sign that sheep gut was different from cow gut, nor that the different winding construction of string ST1 affected its mechanical properties.

The apparent coupling between humidity-induced changes in the string linear density and complementary changes in the string tension, such that there was almost no net effect on the string frequency when the strings were not creeping, was observed consistently during the humidity-variation tests. This behaviour was observed across a number of different strings, with a range of twist angles from 24${}^{\circ }$ to 36${}^{\circ }$, and tested on two different sets of equipment. This coupling effect appeared to be entirely independent of the twist angle (and the surface finish), which would seem to contradict the previous suggestion that the string tension was coupled to changes in the linear density through the twisted structure of the string and the radial expansion and contraction of its component gut strips [16]. Perhaps there was some sort of more general volumetric effect involved; but at this point, this is just speculation.

When the strings were creeping, there was some suggestion that the creep rate might increase with the twist angle, which would seem consistent with the reduction in the slow measure of Young’s modulus $E_{S}$ as the twist angle was increased.

In conjunction with the variation in the Young’s modulus, the longitudinal CLTE of the varnished strings also varied with the twist angle. The CLTE was negative, with the strings trying to contract as the temperature increased, and became larger (more negative) as the twist angle was increased.

There was possibly also some reduction in the thermal tuning sensitivity of the varnished strings as the twist angle was increased, linked to variations in the string tension, but the effect was small and the evidence far from clear.

Overall, it would seem far more important to set the twist angle to get the best compromise between the string strength and the high-frequency damping and inharmonicity, rather than placing any consideration on these other more minor effects.

### 4.2. Surface Finish Effects

When the surface finish part of this study was devised, it was expected that the more important aspect of the string finishing would be whether or not the string had been ground, leaving a large number of cut sections on the gut strips. It was thought that the varnish would crack when the strings were stretched, and would no longer provide an effective vapour barrier. Consequently, the inclusion of the unvarnished strings (S5P, S5U, ST1) was almost an afterthought.

In fact, whether or not the string was varnished turned out to make a considerable difference in the strings’ sensitivity to humidity changes. This was seen most dramatically in the humidity-variation tests where the string linear density tracked the humidity changes much faster than it did for the varnished strings, and the triggered creep episodes progressed much more quickly.

The other major difference between the varnished and unvarnished strings was in their response to temperature changes. Whereas the thermal sensitivity of the varnished strings was dominated by the changes in the string tension, the unvarnished strings displayed more significant changes in linear density, coupled with smaller changes in tension, with the overall thermal tuning sensitivity, for the strings held at constant length, being more finely balanced and closer to zero. This raises the possibility that the thermal tuning sensitivity of gut strings could perhaps be adjusted by using alternative coatings with different degrees of permeability. Not surprisingly, grinding made a difference for the unvarnished strings, primarily in their tension sensitivity.

Holding the string tension constant greatly reduced the thermal tuning sensitivity for the varnished strings, but generally made things worse for the unvarnished strings. The thermal sensitivity in the Young’s modulus was also much smaller for the unvarnished strings.

Finally, the loss factor plots suggested, perhaps counter-intuitively, that the high-frequency damping might be slightly lower for the varnished strings than for the unvarnished strings but the effect was very small, and the evidence was rather thin.

In contrast, with the exception for the unvarnished strings described above, there were no significant differences found between the ground and unground strings. It would appear that, in providing a barrier to humidity changes, the varnish was also closing up the otherwise exposed cut sections on the ground strings.

There appeared to be some minor differences in terms of the creep rates during the humidity-variation tests, with the unground strings creeping slightly faster than the ground strings, and in the high-frequency damping behaviour seen in the loss factor plots, with the unground strings possibly exhibiting slightly less damping. But neither of these effects was at all significant, and the evidence for them was again rather thin.

### 4.3. Creep Behaviour

The humidity-variation tests demonstrated that additional episodes of string creep could be triggered by raising the humidity and hence the string linear density. As seen previously for creep episodes induced by increases in the applied stress or temperature, there appeared to be a threshold effect, with further creep only being triggered once the linear density had exceeded its previous maximum, and the additional creep episodes appeared to behave exponentially, decaying towards some new settling point.

The breaking strength results at different strain rates confirmed the expectation that the breaking stress would fall as the strain rate was reduced. The intuitive explanation for this behaviour is that a slower strain rate would allow more time for creep to occur, weakening the string.

The Young’s modulus $E_{X}$ values taken from the stress–strain gradients, however, presented something of a puzzle: they were consistently lower than the slow measure of Young’s modulus $E_{S}$, which was derived from the long-term post-creep stress–strain responses. In contrast, the Young’s modulus values obtained in an apparently similar manner by Bell and Firth [14] lay between the $E_{S}$ and $E_{T}$ values. A possible explanation for the lower $E_{X}$ values is that the continuously increasing stress applied during the breaking strength tests resulted in significantly greater levels of plastic creep, compared to that experienced when the strings were allowed to settle between each step increase in stress when the target fundamental frequency was changed. This does not explain, though, the difference between the $E_{X}$ values and those from Bell and Firth.

### 4.4. Corrections to the Results Reported for the Previous Study [1]

As described in the main text, some results reported in [1] were affected by the insufficient characterisation of the load cell used for the gut string tests in that study. The derived thermal compensation functions, used to correct for the changes in the load cell gain and zero offset, and in the gain of the associated electronics, did not extend well to lower temperatures. Consequently, tension measurements taken at temperatures below about 20 ${}^{\circ }$C, and especially for loads above 200 N were unreliable and frequently inaccurate. The most important impact was on the measurements of the thermal tuning sensitivity of the gut strings held at constant tension, shown in Figure 9c of [1], and the λ and ψ results shown in Figures 10c and 10f of [1]. The results shown here in Figure 9c, Figure 10a, and Figure 10c should be referred to instead. The results for string G3b may have been particularly badly affected by its being tested during the colder winter months, while the other gut strings had most of their main-sequence tests run during warmer months. All of the results at the base temperature of $T_{0}=20^{\circ }$C, as well as the thermal variation in the Young’s modulus $E_{T}$, appear to have been largely unaffected by the problems with the load cell characterisation. However, the whole of section 4 in [1] should also be ignored since it was based on erroneous data. The curious behaviour shown in Figure 14 of [1] was an artefact of the load cell thermal compensation functions failing to extend to lower temperatures; this behaviour disappeared when a correctly characterised and compensated load cell was used. Fortunately, discarding that section has very little impact on the rest of the paper, and the conclusions not directly related to these erroneous results all still hold.

## Figures and Tables

**Table 1:** The set of strings studied, showing the unstretched string parameters, and the target fundamental frequencies used for testing. The number in the first column provides a unique reference for each string or string section. It can be used to cross-reference with the summary data sets submitted with this paper and the larger data archive [21].

No.	Diameter (mm)	Density (kg/m${}^{3}$)	Twist Angle	Surface Finish	Test Rig	Target Test Frequencies (Hz)	Test Period
				TWIST ANGLE STUDY			
T4Ba	1.680	1312	33.9${}^{\circ }$	Normal	Rig 1	174, 235, 288, 306, 324	Oct 2018–Jan 2019
T2Ba	1.626	1323	29.3${}^{\circ }$	Normal	Rig 1	174, 235	February 2019
T4Bb	1.680	1312	33.9${}^{\circ }$	Normal	Rig 1 ${}^{‡}$	174, 235, 288	Mar–Apr 2019
T4Ac	1.573	1326	24.0${}^{\circ }$	Normal	Rig 2 ${}^{†}$	174, 235, 288, 324	May–Jul 2019
T2Bb	1.626	1323	29.3${}^{\circ }$	Normal	Rig 2 ${}^{†}$	174, 235, 288	Jul–Sep 2019
T2Ab	1.619	1323	36.2${}^{\circ }$	Normal	Rig 2 ${}^{†§}$	174, 235	Oct–Dec 2019
T3Ba	1.587	1327	28.7${}^{\circ }$	Normal	Rig 2 ${}^{†}$	174, 235, 288, 324	Jan–Mar 2020
T3Bb	1.587	1327	28.7${}^{\circ }$	Normal	Rig 2	174, 235, 288, 324 *	Mar–Jul 2020
T3Aa	1.540	1328	23.5${}^{\circ }$	Normal	Rig 1	174, 235, 288, 324 *	Mar–Aug 2020
T1Ba	1.760	1320	35.2${}^{\circ }$	Normal	Rig 2	174, 235, 288, **300** *	Aug–Dec 2020
T1Aa	1.616	1327	36.2${}^{\circ }$	Normal	Rig 1	174, 235, 288, 324 *	Aug–Dec 2020
				SURFACE FINISH STUDY			
S1Pa	1.636	1311	30.7${}^{\circ }$	Normal	Rig 1	174, 235, 288, 324 *	Sep 2021–Jan 2022
S12Ua	1.641	1320	29.2${}^{\circ }$	Unground	Rig 2	174, 235, 288, 324 *	Sep 2021–Jan 2022
S11Ua	1.864	1318	31.2${}^{\circ }$	Unground	Rig 1	174	January 2022
ST1a	1.990	1283	58.3${}^{\circ }$	Unground and Unvarnished	Rig 2	174	January 2022
S3Ua	1.485	1325	25.7${}^{\circ }$	Unground	Rig 1	174, 235, 288, 324 *	Jan–Apr 2022
S2Pa	1.487	1326	28.3${}^{\circ }$	Normal	Rig 2	174, 235, 288, 324 *	Jan–Apr 2022
S11Pa	1.668	1318	31.2${}^{\circ }$	Normal	Rig 1	174, 235, 288	Apr–Jun 2022
ST1b	1.990	1283	58.3${}^{\circ }$	Unground and Unvarnished	Rig 2	**135**, **156**, 174	Apr–Jun 2022
S12Pa	1.545	1320	29.2${}^{\circ }$	Normal	Rig 2	174, 235, 288, 324	Jun–Aug 2022
S3Pa	1.328	1325	25.7${}^{\circ }$	Normal	Rig 1	174, 235, 288, 324	Jun–Aug 2022
S2Pb	1.487	1326	28.3${}^{\circ }$	Normal	Rig 1	174, 235, 288, 324 *	Aug–Nov 2022
S5Pa	1.492	1329	28.4${}^{\circ }$	Unvarnished	Rig 2	174, 235, 288, 324 *	Aug–Nov 2022
S12Ub	1.641	1320	29.2${}^{\circ }$	Unground	Rig 1	174, 235, 288, 324 *	Nov 2022–Jan 2023
S5Ua	1.608	1329	28.4${}^{\circ }$	Unground and Unvarnished	Rig 2	174, 235, 288, 324 *	Nov 2022–Jan 2023

* Tests marked with an asterisk had a humidity-variation test run at the end of the main test sequence. ${}^{†}$ A number of tests were run using Rig 2 with the load cell from Rig 1, while the load cell from Rig 2 was being re-characterised using the test chamber for Rig 1. ${}^{‡}$ One test sequence was run with Rig 1 swapped into the test chamber for Rig 2. ${}^{§}$ The SHT75 temperature and humidity sensor on Rig 2 failed near the end of the test sequence for string section T2Ab, while the load cell from Rig 2 was being re-characterised using the test chamber for Rig 1. Later tests were run with the SHT85 sensor in both test rigs.

## Data Availability

Data is available in Supplementary Materials.

## Supplementary Materials

The following supporting information can be downloaded at https://www.mdpi.com/article/10.3390/ma16155444/s1. Summary and measurement data files, plus a file guide and the Python code for generating the plots, except for Figure 8. A comprehensive data archive is also available [21].

## Abbreviations

The following abbreviations are used in this manuscript:

CLTE	Coefficient of Linear Thermal Expansion
FFT	Fast Fourier Transform
RH	Relative Humidity
RMS	Root Mean Square
