# Supplementary material for: Effects of Twisting and Surface Finish on the Mechanical Properties of Natural Gut Harp Strings
Source: Materials (Basel). 2023 Aug 3;16(15):5444. doi: 10.3390/ma16155444 (PMC10419811; doi:10.3390/ma16155444)
Supplement: Supplementary file 1 [file materials-16-05444-s001.zip › Figures for twist angle and surface finish studies.html]

Figures for twist angle and surface finish studies


# Figures for Twist Angle and Surface Finish Studies of Natural Gut Harp Strings¶

In [1]:

```
import xlrd
import numpy as np
import numpy.polynomial.polynomial as poly
from scipy.optimize import curve_fit
import matplotlib.pyplot as plt
import matplotlib.lines as mlines
import matplotlib.ticker as ticker
from matplotlib.gridspec import GridSpec
from mpl_toolkits.mplot3d import Axes3D
#
# enable plots to be shown in cells
%matplotlib inline

K_scaler = 600.0 / np.log(2.0)    # K
d_range = 0.005                   # mm - diameter measurement accuracy limit
psi_weight = 0                    # weighting to give to psi term in calculating CLTE

def r_squared(y_act, y_est):
    y_mean = np.mean(y_act)
    sumsq_tot = np.sum((y_act - y_mean)**2)
    sumsq_res = np.sum((y_act - y_est)**2)
    return (1.0 - sumsq_res/sumsq_tot)
```

## Read summary data for twist angle study¶

In [2]:

```
global_excl_old_lc2 = True # global setting to exclude results dependent on original limited characterisation of loadcell 2

# plot colours
T1_pen = 'black'
T1A_pen = 'brown'
T1B_pen = 'purple'
T2_pen = 'red'
T2A_pen = 'red'
T2B_pen = 'orange'
T3_pen = 'purple'
T3A_pen = 'magenta'
T3B_pen = 'purple'
T4_pen = 'blue'
T4A_pen = 'cyan'
T4B_pen = 'blue'
gut_pen = 'green'

# markers
lo_twist_mkr = 'o'
hi_twist_mkr = 'o'
T1A_mkr = hi_twist_mkr
T1B_mkr = lo_twist_mkr
T2A_mkr = hi_twist_mkr
T2B_mkr = lo_twist_mkr
T3A_mkr = lo_twist_mkr
T3B_mkr = hi_twist_mkr
T4A_mkr = lo_twist_mkr
T4B_mkr = hi_twist_mkr

# legends
T1_leg = 'T1'
T2_leg = 'T2'
T3_leg = 'T3'
T4_leg = 'T4'
gut_leg = 'G2, G3, G5'

T1A_leg = 'T1A (36.2)'
T1B_leg = 'T1B (35.2)'
T2A_leg = 'T2A (36.2)'
T2B_leg = 'T2B (29.3)'
T3A_leg = 'T3A (23.5)'
T3B_leg = 'T3B (28.7)'
T4A_leg = 'T4A (24.0)'
T4B_leg = 'T4B (33.9)'

string_list_A = ['T4Ba','T2Ba','T4Bb','T4Ac','T2Bb','T2Ab','T3Ba','T3Bb','T3Aa','T1Ba','T1Aa']
dfdT_F_list_A =                             ['T2Bb','T2Ab','T3Ba','T3Bb','T3Aa','T1Ba','T1Aa']
plot_list_A =   ['T1Aa','T2Ab','T1Ba','T4Ba','T4Bb','T2Ba','T2Bb','T3Ba','T3Bb','T4Ac','T3Aa']

T4Ba = {'rig':1,'lc':1,'start_row':10,'stop_row':16,'pen':T4B_pen,'ppen':T4_pen,'mkr':T4B_mkr,'leg':'T4Ba','sleg':T4B_leg,'pleg':T4_leg}
T2Ba = {'rig':1,'lc':1,'start_row':20,'stop_row':23,'pen':T2B_pen,'ppen':T2_pen,'mkr':T2B_mkr,'leg':'T2Ba','sleg':T2B_leg,'pleg':T2_leg}
T4Bb = {'rig':1,'lc':1,'start_row':30,'stop_row':34,'pen':T4B_pen,'ppen':T4_pen,'mkr':T4B_mkr,'leg':'T4Bb','sleg':T4B_leg,'pleg':T4_leg}
T4Ac = {'rig':2,'lc':1,'start_row':40,'stop_row':45,'pen':T4A_pen,'ppen':T4_pen,'mkr':T4A_mkr,'leg':'T4Ac','sleg':T4A_leg,'pleg':T4_leg}
T2Bb = {'rig':2,'lc':1,'start_row':50,'stop_row':54,'pen':T2B_pen,'ppen':T2_pen,'mkr':T2B_mkr,'leg':'T2Bb','sleg':T2B_leg,'pleg':T2_leg}
T2Ab = {'rig':2,'lc':1,'start_row':60,'stop_row':63,'pen':T2A_pen,'ppen':T2_pen,'mkr':T2A_mkr,'leg':'T2Ab','sleg':T2A_leg,'pleg':T2_leg}
T3Ba = {'rig':2,'lc':1,'start_row':70,'stop_row':75,'pen':T3B_pen,'ppen':T3_pen,'mkr':T3B_mkr,'leg':'T3Ba','sleg':T3B_leg,'pleg':T3_leg}
T3Bb = {'rig':2,'lc':2,'start_row':80,'stop_row':85,'pen':T3B_pen,'ppen':T3_pen,'mkr':T3B_mkr,'leg':'T3Bb','sleg':T3B_leg,'pleg':T3_leg}
T3Aa = {'rig':1,'lc':1,'start_row':90,'stop_row':95,'pen':T3A_pen,'ppen':T3_pen,'mkr':T3A_mkr,'leg':'T3Aa','sleg':T3A_leg,'pleg':T3_leg}
T1Ba = {'rig':2,'lc':2,'start_row':100,'stop_row':105,'pen':T1B_pen,'ppen':T1_pen,'mkr':T1B_mkr,'leg':'T1Ba','sleg':T1B_leg,'pleg':T1_leg}
T1Aa = {'rig':1,'lc':1,'start_row':110,'stop_row':115,'pen':T1A_pen,'ppen':T1_pen,'mkr':T1A_mkr,'leg':'T1Aa','sleg':T1A_leg,'pleg':T1_leg}


# READ SUMMARY DATA FOR TWIST RATIO STUDY
# Note: use zero-indexing for row and column numbers
# connect to data source
file_location = "./twist ratio summary.xls"
workbook1 = xlrd.open_workbook(file_location)
data_sheet = workbook1.sheet_by_index(0)
older_gut = workbook1.sheet_by_index(1)
bell_firth = workbook1.sheet_by_index(2)
#
# List sheet names
sheet_names = workbook1.sheet_names()
print('Sheet Names:', sheet_names)

# read in E versus twist angle data for previous gut strings
# string order in arrays is G2 G3 G5
gut_start_row = 4
gut_stop_row = 7
gut_angle_col = 2
gut_Es_col = 3
gut_Et_col = 4
gut_Eb_col = 5
gut_angle = [older_gut.cell_value(i, gut_angle_col) for i in range(gut_start_row, gut_stop_row)]
gut_Es = [older_gut.cell_value(i, gut_Es_col) for i in range(gut_start_row, gut_stop_row)]
gut_Et = [older_gut.cell_value(i, gut_Et_col) for i in range(gut_start_row, gut_stop_row)]
gut_Eb = [older_gut.cell_value(i, gut_Eb_col) for i in range(gut_start_row, gut_stop_row)]

# read in E versus twist angle data for strings from Bell-Firth study
bf_angle = [bell_firth.cell_value(i, 8) for i in range(3,8)]
bf_Et = [bell_firth.cell_value(i, 6) for i in range(3,8)]
bf_Et_SD = [bell_firth.cell_value(i, 7) for i in range(3,8)]
bf_br_strength = [bell_firth.cell_value(i, 4) for i in range(3,8)]
bf_br_strength_SD = [bell_firth.cell_value(i, 5) for i in range(3,8)]
bf_weights = [bell_firth.cell_value(i, 1) for i in range(3,8)]


# read main data set for tested strings
# columns
param_col = 2                # individual parameters
length_adj_col = 5           # m          measured length adjustment at start of Young's modulus Et test
d_col = 6                    # mm         string diameter
Fw_col = 7                   # N          measured tension at start of Young's modulus Et test
Et_col = 8                   # GPa        Young's modulus from tension modulation 
dEtdT_col = 9                # GPa/°C     thermal variation in Young's modulus
Eb_col = 12                  # GPa        Young's modulus from bending stiffness (manual processing)
Eb_script_col = 13           # GPa        Young's modulus from bending stiffness (script processing)
F0_col = 15                  # N          measured tension during constant length test
dFdT_col = 16                # N/°C       thermal variation in tension during constant length test
mu_col = 22                  # kg/m       linear density (20°C) during constant length test         
psi_col = 23                 #            thermal relative sensitivity of linear density during constant length test
dfdT_L_col = 24              # cent/°C    thermal frequency sensitivity during constant length test
dfdT_F_col = 27              # cent/°C    thermal frequency sensitivity during constant tension test
dfdT_E_col = 30              # cent/°C    thermal frequency sensitivity during Young's modulus test

offset_diff_value_A = []
offset_diff_angle_A = []
offset_diff_diameter_A = []

for string in string_list_A:
    sdict = globals()[string]
    # read data
    start_row = sdict['start_row']
    stop_row = sdict['stop_row']
    sdict['points'] = stop_row - start_row - 1
    print('STRING %s on test rig %d and loadcell %d with %d points' % (string, sdict['rig'], sdict['lc'], sdict['points']))
    #
    L_v = data_sheet.cell_value(start_row, param_col)
    L_total = data_sheet.cell_value(start_row+1, param_col)
    #
    sdict['twist_angle'] = data_sheet.cell_value(start_row+2, param_col)
    length_adj_offset = data_sheet.cell_value(start_row+3, param_col)
    if(sdict['points'] >= 3):
        alternate_offset = data_sheet.cell_value(start_row+4, param_col)
        offset_diff_value_A.append(alternate_offset - length_adj_offset)
        offset_diff_angle_A.append(sdict['twist_angle'])
        offset_diff_diameter_A.append(data_sheet.cell_value(start_row, d_col))
    else:
        alternate_offset = np.nan
    #
    diameter0 = np.array([data_sheet.cell_value(i, d_col) for i in range(start_row, stop_row)])
    sdict['mu0'] = np.array([data_sheet.cell_value(i, mu_col) for i in range(start_row, stop_row)])
    #
    start_row1 = start_row + 1
    sdict['length_adj'] = np.array([data_sheet.cell_value(i, length_adj_col) for i in range(start_row1, stop_row)])
    sdict['Fw'] = np.array([data_sheet.cell_value(i, Fw_col) for i in range(start_row1, stop_row)])
    sdict['Et'] = np.array([data_sheet.cell_value(i, Et_col) for i in range(start_row1, stop_row)])
    sdict['dEtdT'] = np.array([data_sheet.cell_value(i, dEtdT_col) for i in range(start_row1, stop_row)])
    sdict['Eb'] = np.array([data_sheet.cell_value(i, Eb_col) if(data_sheet.cell(i, Eb_col).ctype not in [0,6]) 
                            else np.nan for i in range(start_row1, stop_row)])
    sdict['Eb_script'] = np.array([data_sheet.cell_value(i, Eb_script_col) if(data_sheet.cell(i, Eb_script_col).ctype not in [0,6]) 
                                   else np.nan for i in range(start_row1, stop_row)])
    sdict['F0'] = np.array([data_sheet.cell_value(i, F0_col) for i in range(start_row1, stop_row)])
    sdict['dFdT'] = np.array([data_sheet.cell_value(i, dFdT_col) for i in range(start_row1, stop_row)])
    sdict['psi'] = np.array([data_sheet.cell_value(i, psi_col) for i in range(start_row1, stop_row)])
    sdict['dfdT_L'] = np.array([data_sheet.cell_value(i, dfdT_L_col) for i in range(start_row1, stop_row)])
    if(string in dfdT_F_list_A):
        sdict['dfdT_F'] = np.array([data_sheet.cell_value(i, dfdT_F_col) for i in range(start_row1, stop_row)])
    else:
        sdict['dfdT_F'] = np.array([np.nan for i in range(start_row1, stop_row)])
    sdict['dfdT_E'] = np.array([data_sheet.cell_value(i, dfdT_E_col) for i in range(start_row1, stop_row)])

    # calculate derived values
    # density values
    sdict['area0'] = np.pi * diameter0**2 / 4.0  # mm^2
    sdict['rho0'] = rho0 = 1000000 * sdict['mu0'] / sdict['area0']        # kg/m^3
    # density ratio components    
    sdict['den_ratio'] = rho0 / rho0[0]
    sdict['den_err_plus'] = (4000000 * sdict['mu0'] / (np.pi * (diameter0 - d_range)**2) - rho0) / rho0[0]
    sdict['den_err_minus'] = (rho0 - 4000000 * sdict['mu0'] / (np.pi * (diameter0 + d_range)**2)) / rho0[0]
    sdict['den_err_plus'][0] = 0.0
    sdict['den_err_minus'][0] = 0.0
    # stress and strain  -  area (mm^2) and stress (MPa)
    sdict['stress'] = sdict['Fw'] / sdict['area0'][1:] # MPa
    sdict['stress0'] = np.insert(sdict['stress'], 0, 0.0, axis=0)
    sdict['inv_stress'] = 1.0 / sdict['stress']    # inverse stress values
    sdict['strain'] = np.exp((sdict['length_adj'] + length_adj_offset) / L_total) - 1
    sdict['strain0'] = np.insert(sdict['strain'], 0, 0.0, axis=0)
    sdict['alt_strain'] = np.exp((sdict['length_adj'] + alternate_offset) / L_total) - 1
    sdict['alt_strain0'] = np.insert(sdict['alt_strain'], 0, 0.0, axis=0)
    # Es (GPa) and corresponding stress values (MPa)
    sdict['Es'] = []
    sdict['Es_stress'] = []
    for i in range(0, len(sdict['stress']), 1):
        sdict['Es'].append((sdict['stress0'][i+1]-sdict['stress0'][i])/(1000.0*(sdict['strain0'][i+1]-sdict['strain0'][i])))
        sdict['Es_stress'].append((sdict['stress0'][i]+sdict['stress0'][i+1])/2.0)
    # frequency deviation components
    sdict['lambda'] = sdict['dFdT'] / sdict['F0']    # lambda = (1/F).dF/dT
    sdict['Klambda'] = K_scaler * sdict['lambda']    # K.lambda
    sdict['Kpsi'] = K_scaler * sdict['psi']          # K.psi
    # CLTE and components
    sdict['dstressdT'] = sdict['dFdT'] / sdict['area0'][1:]
    AEterm = 1000.0 * sdict['area0'][1:] * sdict['Et']  # correct for mm^2 and GPa
    FbyAE = sdict['F0'] / AEterm
    # (F/AE^2) dE/dT
    sdict['Eterm'] = FbyAE * sdict['dEtdT'] / sdict['Et']
    # -(1/AE) dF/dT
    sdict['Fterm'] = - sdict['dFdT'] / AEterm
    # -(F/AE) psi
    sdict['Pterm'] = - FbyAE * sdict['psi']
    # CLTE
    sdict['CLTE'] = sdict['Eterm'] + sdict['Fterm'] + (psi_weight * sdict['Pterm'])

loaded_twist_set = True
```

```
Sheet Names: ['summary data', 'Gut.E.vs.angle', 'Bell.Firth', 'data file list', 'Test Type Key']
STRING T4Ba on test rig 1 and loadcell 1 with 5 points
STRING T2Ba on test rig 1 and loadcell 1 with 2 points
STRING T4Bb on test rig 1 and loadcell 1 with 3 points
STRING T4Ac on test rig 2 and loadcell 1 with 4 points
STRING T2Bb on test rig 2 and loadcell 1 with 3 points
STRING T2Ab on test rig 2 and loadcell 1 with 2 points
STRING T3Ba on test rig 2 and loadcell 1 with 4 points
STRING T3Bb on test rig 2 and loadcell 2 with 4 points
STRING T3Aa on test rig 1 and loadcell 1 with 4 points
STRING T1Ba on test rig 2 and loadcell 2 with 4 points
STRING T1Aa on test rig 1 and loadcell 1 with 4 points
```

## Read summary data for surface finish study¶

In [3]:

```
# plot colours
S1U_pen = 'black'
S11U_pen = 'black'
S1P_pen = 'red'
S11P_pen = 'magenta'
S12U_pen = 'purple'
S2P_pen = 'blue'
S12P_pen = 'black'
S3U_pen = 'green'
S3P_pen = 'lime'
S5U_pen = 'orange'
S5P_pen = 'cyan'
ST1_pen = 'brown'

# markers
unground_mkr = 'd'
ground_mkr = 's'
S1U_mkr = unground_mkr
S11U_mkr = unground_mkr
S12U_mkr = unground_mkr
S3U_mkr = unground_mkr
S5U_mkr = unground_mkr
ST1_mkr = unground_mkr
S1P_mkr = ground_mkr
S11P_mkr = ground_mkr
S2P_mkr = ground_mkr
S12P_mkr = ground_mkr
S3P_mkr = ground_mkr
S5P_mkr = ground_mkr

#string labels
S1U_leg = 'S1U'
S11U_leg = 'S11U'
S1P_leg = 'S1P'
S11P_leg = 'S11P'
S12U_leg = 'S12U'
S2P_leg = 'S2P'
S12P_leg = 'S12P'
S3U_leg = 'S3U'
S3P_leg = 'S3P'
S5U_leg = 'S5U'
S5P_leg = 'S5P'
ST1_leg = 'ST1'
 
string_list_S = ['S1Pa','S12Ua','S11Ua','ST1a','S3Ua','S2Pa','S11Pa','ST1b','S12Pa','S3Pa','S2Pb','S5Pa','S12Ub','S5Ua']
plot_list_S =   ['S11Ua','S1Pa','S11Pa','S12Ua','S12Ub','S12Pa','S2Pa','S2Pb','S3Ua','S3Pa','S5Ua','S5Pa','ST1a','ST1b']
plot_list_SV =  ['S11Ua','S1Pa','S11Pa','S12Ua','S12Ub','S12Pa','S2Pa','S2Pb','S3Ua','S3Pa']

S1Pa =  {'rig':1,'start_row':10, 'stop_row':15, 'pen':S1P_pen, 'mkr':S1P_mkr, 'leg':'S1Pa', 'sleg':S1P_leg}
S12Ua = {'rig':2,'start_row':20, 'stop_row':25, 'pen':S12U_pen,'mkr':S12U_mkr,'leg':'S12Ua','sleg':S12U_leg}
S11Ua = {'rig':1,'start_row':30, 'stop_row':32, 'pen':S11U_pen,'mkr':S11U_mkr,'leg':'S11Ua','sleg':S11U_leg}
ST1a =  {'rig':2,'start_row':40, 'stop_row':42, 'pen':ST1_pen, 'mkr':ST1_mkr, 'leg':'ST1a', 'sleg':ST1_leg}
S3Ua =  {'rig':1,'start_row':50, 'stop_row':55, 'pen':S3U_pen, 'mkr':S3U_mkr, 'leg':'S3Ua', 'sleg':S3U_leg}
S2Pa =  {'rig':2,'start_row':60, 'stop_row':65, 'pen':S2P_pen, 'mkr':S2P_mkr, 'leg':'S2Pa', 'sleg':S2P_leg}
S11Pa = {'rig':1,'start_row':70, 'stop_row':74, 'pen':S11P_pen,'mkr':S11P_mkr,'leg':'S11Pa','sleg':S11P_leg}
ST1b =  {'rig':2,'start_row':80, 'stop_row':84, 'pen':ST1_pen, 'mkr':ST1_mkr, 'leg':'ST1b', 'sleg':ST1_leg}
S12Pa = {'rig':2,'start_row':90, 'stop_row':95, 'pen':S12P_pen,'mkr':S12P_mkr,'leg':'S12Pa','sleg':S12P_leg}
S3Pa =  {'rig':1,'start_row':100,'stop_row':105,'pen':S3P_pen, 'mkr':S3P_mkr, 'leg':'S3Pa', 'sleg':S3P_leg}
S2Pb =  {'rig':1,'start_row':110,'stop_row':115,'pen':S2P_pen, 'mkr':S2P_mkr, 'leg':'S2Pb', 'sleg':S2P_leg}
S5Pa =  {'rig':2,'start_row':120,'stop_row':125,'pen':S5P_pen, 'mkr':S5P_mkr, 'leg':'S5Pa', 'sleg':S5P_leg}
S12Ub = {'rig':1,'start_row':130,'stop_row':135,'pen':S12U_pen,'mkr':S12U_mkr,'leg':'S12Ub','sleg':S12U_leg}
S5Ua =  {'rig':2,'start_row':140,'stop_row':145,'pen':S5U_pen, 'mkr':S5U_mkr, 'leg':'S5Ua', 'sleg':S5U_leg}


# READ SUMMARY DATA FOR SURFACE FINISH STUDY
# Note: use zero-indexing for row and column numbers
# connect to data source
file_location = "./unground summary.xls"
workbook1 = xlrd.open_workbook(file_location)
data_sheet = workbook1.sheet_by_index(0)
#
# columns
param_col = 2                # individual parameters
length_adj_col = 5           # m          measured length adjustment at start of Young's modulus Et test
d_col = 6                    # mm         string diameter
Fw_col = 7                   # N          measured tension at start of Young's modulus Et test
Et_col = 8                   # GPa        Young's modulus from tension modulation 
dEtdT_col = 9                # GPa/°C     thermal variation in Young's modulus
Eb_col = 12                  # GPa        Young's modulus from bending stiffness (manual processing)
Eb_script_col = 13           # GPa        Young's modulus from bending stiffness (script processing)
F0_col = 15                  # N          measured tension during constant length test
dFdT_col = 16                # N/°C       thermal variation in tension during constant length test
mu_col = 22                  # kg/m       linear density (20°C) during constant length test         
psi_col = 23                 #            thermal relative sensitivity of linear density during constant length test
dfdT_L_col = 24              # cent/°C    thermal frequency sensitivity during constant length test
dfdT_E_col = 27              # cent/°C    thermal frequency sensitivity during Young's modulus test

offset_diff_value_S = []
offset_diff_angle_S = []
offset_diff_diameter_S = []

for string in string_list_S:
    sdict = globals()[string]
    # read data
    start_row = sdict['start_row']
    stop_row = sdict['stop_row']
    sdict['points'] = stop_row - start_row - 1
    print('STRING %s on test rig %d with %d points' % (string, sdict['rig'], sdict['points']))
    #
    L_v = data_sheet.cell_value(start_row, param_col)
    L_total = data_sheet.cell_value(start_row+1, param_col)
    #
    sdict['twist_angle'] = data_sheet.cell_value(start_row+2, param_col)
    length_adj_offset = data_sheet.cell_value(start_row+3, param_col)
    if(sdict['points'] >= 3):
        alternate_offset = data_sheet.cell_value(start_row+4, param_col)
        offset_diff_value_S.append(alternate_offset - length_adj_offset)
        offset_diff_angle_S.append(sdict['twist_angle'])
        offset_diff_diameter_S.append(data_sheet.cell_value(start_row, d_col))
    else:
        alternate_offset = np.nan
    #
    diameter0 = np.array([data_sheet.cell_value(i, d_col) for i in range(start_row, stop_row)])
    sdict['mu0'] = np.array([data_sheet.cell_value(i, mu_col) for i in range(start_row, stop_row)])
    #
    start_row1 = start_row + 1
    sdict['length_adj'] = np.array([data_sheet.cell_value(i, length_adj_col) for i in range(start_row1, stop_row)])
    sdict['Fw'] = np.array([data_sheet.cell_value(i, Fw_col) for i in range(start_row1, stop_row)])
    sdict['Et'] = np.array([data_sheet.cell_value(i, Et_col) for i in range(start_row1, stop_row)])
    sdict['dEtdT'] = np.array([data_sheet.cell_value(i, dEtdT_col) for i in range(start_row1, stop_row)])
    sdict['Eb'] = np.array([data_sheet.cell_value(i, Eb_col) if(data_sheet.cell(i, Eb_col).ctype not in [0,6]) 
                            else np.nan for i in range(start_row1, stop_row)])
    sdict['Eb_script'] = np.array([data_sheet.cell_value(i, Eb_script_col) if(data_sheet.cell(i, Eb_script_col).ctype not in [0,6]) 
                                   else np.nan for i in range(start_row1, stop_row)])
    sdict['F0'] = np.array([data_sheet.cell_value(i, F0_col) for i in range(start_row1, stop_row)])
    sdict['dFdT'] = np.array([data_sheet.cell_value(i, dFdT_col) for i in range(start_row1, stop_row)])
    sdict['psi'] = np.array([data_sheet.cell_value(i, psi_col) for i in range(start_row1, stop_row)])
    sdict['dfdT_L'] = np.array([data_sheet.cell_value(i, dfdT_L_col) for i in range(start_row1, stop_row)])
    sdict['dfdT_E'] = np.array([data_sheet.cell_value(i, dfdT_E_col) for i in range(start_row1, stop_row)])
    
    # calculate derived values
    # density values
    sdict['area0'] = np.pi * diameter0**2 / 4.0  # mm^2
    sdict['rho0'] = rho0 = 1000000 * sdict['mu0'] / sdict['area0']        # kg/m^3
    # density ratio components    
    sdict['den_ratio'] = rho0 / rho0[0]
    sdict['den_err_plus'] = (4000000 * sdict['mu0'] / (np.pi * (diameter0 - d_range)**2) - rho0) / rho0[0]
    sdict['den_err_minus'] = (rho0 - 4000000 * sdict['mu0'] / (np.pi * (diameter0 + d_range)**2)) / rho0[0]
    sdict['den_err_plus'][0] = 0.0
    sdict['den_err_minus'][0] = 0.0
    # stress and strain  -  area (mm^2) and stress (MPa)
    sdict['stress'] = sdict['Fw'] / sdict['area0'][1:] # MPa
    sdict['stress0'] = np.insert(sdict['stress'], 0, 0.0, axis=0)
    sdict['inv_stress'] = 1.0 / sdict['stress']    # inverse stress values
    sdict['strain'] = np.exp((sdict['length_adj'] + length_adj_offset) / L_total) - 1
    sdict['strain0'] = np.insert(sdict['strain'], 0, 0.0, axis=0)
    sdict['alt_strain'] = np.exp((sdict['length_adj'] + alternate_offset) / L_total) - 1
    sdict['alt_strain0'] = np.insert(sdict['alt_strain'], 0, 0.0, axis=0)
    # Es (GPa) and corresponding stress values (MPa)
    sdict['Es'] = []
    sdict['Es_stress'] = []
    for i in range(0, len(sdict['stress']), 1):
        sdict['Es'].append((sdict['stress0'][i+1]-sdict['stress0'][i])/(1000.0*(sdict['strain0'][i+1]-sdict['strain0'][i])))
        sdict['Es_stress'].append((sdict['stress0'][i]+sdict['stress0'][i+1])/2.0)
    # frequency deviation components
    sdict['lambda'] = sdict['dFdT'] / sdict['F0']    # lambda = (1/F).dF/dT
    sdict['Klambda'] = K_scaler * sdict['lambda']    # K.lambda
    sdict['Kpsi'] = K_scaler * sdict['psi']          # K.psi
    # CLTE and components
    sdict['dstressdT'] = sdict['dFdT'] / sdict['area0'][1:]
    AEterm = 1000.0 * sdict['area0'][1:] * sdict['Et']  # correct for mm^2 and GPa
    FbyAE = sdict['F0'] / AEterm
    # (F/AE^2) dE/dT
    sdict['Eterm'] = FbyAE * sdict['dEtdT'] / sdict['Et']
    # -(1/AE) dF/dT
    sdict['Fterm'] = - sdict['dFdT'] / AEterm
    # -(F/AE) psi
    sdict['Pterm'] = - FbyAE * sdict['psi']
    # CLTE
    sdict['CLTE'] = sdict['Eterm'] + sdict['Fterm'] + (psi_weight * sdict['Pterm'])

loaded_surface_set = True
```

```
STRING S1Pa on test rig 1 with 4 points
STRING S12Ua on test rig 2 with 4 points
STRING S11Ua on test rig 1 with 1 points
STRING ST1a on test rig 2 with 1 points
STRING S3Ua on test rig 1 with 4 points
STRING S2Pa on test rig 2 with 4 points
STRING S11Pa on test rig 1 with 3 points
STRING ST1b on test rig 2 with 3 points
STRING S12Pa on test rig 2 with 4 points
STRING S3Pa on test rig 1 with 4 points
STRING S2Pb on test rig 1 with 4 points
STRING S5Pa on test rig 2 with 4 points
STRING S12Ub on test rig 1 with 4 points
STRING S5Ua on test rig 2 with 4 points
```

## Arrays of average values¶

In [4]:

```
# array sequence is T1A T1B T2A T2B T3A T3B T4A T4B S11U S1P S11P S12U S12P S2P S3U S3P S5U S5P ST1
def assemble_avg_arrays():
    array_list = ['T1A','T1B','T2A','T2B','T3A','T3B','T4A','T4B',
                  'S11U','S1P','S11P','S12U','S12P','S2P','S3U','S3P','S5U','S5P','ST1']
    string_list = string_list_A + string_list_S
    #
    for variable in ['pen','leg','angle','Es','Et','Eb','dfdT_L','Klambda','Kpsi','dEtdT','dstressdT',
                     'CLTE', 'Eterm','Fterm','Pterm']:
        var_array = globals()['avg_'+variable] = []
        for entry in array_list:
            if(variable in ['pen','leg']):
                root = entry[:-1] if(entry[0]=='T') else entry
                root += '_'
                var_array.append(globals()[root+variable])
            elif(variable == 'angle'):
                done = False
                for string in string_list:
                    if((entry in string) and (not done)):
                        var_array.append(globals()[string]['twist_angle'])
                        done = True
            elif(variable in ['Es','Et','Eb','dstressdT','Fterm','Pterm']):
                values = np.array([])
                for string in string_list:
                    if(entry in string):
                        values = np.append(values, globals()[string][variable])
                var_array.append(np.average(values))
            else: # variable in ['dfdT_L','Klambda','Kpsi','dEtdT','CLTE','Eterm']
                values = np.zeros(4)
                for index in range(len(values)):
                    count = 0
                    for string in string_list:
                        if((entry in string) and (len(globals()[string][variable]) > index)):
                            if((string == 'T4Ba') and (index == 3)):
                                values[index] += globals()[string][variable][index+1]
                            else:
                                values[index] += globals()[string][variable][index]
                            count += 1
                    values[index] = values[index] / count if(count > 0) else np.nan
                var_array.append(values)
    
# array sequence is T1A T1B T2A T2B T3A T3B T4A T4B S11U S1P S11P S12U S12P S2P S3U S3P S5U S5P ST1
if(loaded_twist_set and loaded_surface_set):
    assemble_avg_arrays()
```

## Load breaking test data¶

In [5]:

```
G1 = {'pen':gut_pen,'mkr':'s','leg':'G1','sleg':'G1'}
G2 = {'pen':gut_pen,'mkr':'d','leg':'G2','sleg':'G2'}
T1A = {'pen':T1A_pen,'ppen':T1_pen,'mkr':T1A_mkr,'leg':T1A_leg,'sleg':T1A_leg,'pleg':T1_leg}
T1B = {'pen':T1B_pen,'ppen':T1_pen,'mkr':T1B_mkr,'leg':T1B_leg,'sleg':T1B_leg,'pleg':T1_leg}
T2A = {'pen':T2A_pen,'ppen':T2_pen,'mkr':T2A_mkr,'leg':T2A_leg,'sleg':T2A_leg,'pleg':T2_leg}
T2B = {'pen':T2B_pen,'ppen':T2_pen,'mkr':T2B_mkr,'leg':T2B_leg,'sleg':T2B_leg,'pleg':T2_leg}
T3A = {'pen':T3A_pen,'ppen':T3_pen,'mkr':T3A_mkr,'leg':T3A_leg,'sleg':T3A_leg,'pleg':T3_leg}
T3B = {'pen':T3B_pen,'ppen':T3_pen,'mkr':T3B_mkr,'leg':T3B_leg,'sleg':T3B_leg,'pleg':T3_leg}
T4A = {'pen':T4A_pen,'ppen':T4_pen,'mkr':T4A_mkr,'leg':T4A_leg,'sleg':T4A_leg,'pleg':T4_leg}
T4B = {'pen':T4B_pen,'ppen':T4_pen,'mkr':T4B_mkr,'leg':T4B_leg,'sleg':T4B_leg,'pleg':T4_leg}
S1U  =  {'pen':S1U_pen, 'mkr':S1U_mkr, 'leg':S1U_leg, 'sleg':S1U_leg}
S1P  =  {'pen':S1P_pen, 'mkr':S1P_mkr, 'leg':S1P_leg, 'sleg':S1P_leg}
S11U =  {'pen':S11U_pen,'mkr':S11U_mkr,'leg':S11U_leg,'sleg':S11U_leg}
S11P =  {'pen':S11P_pen,'mkr':S11P_mkr,'leg':S11P_leg,'sleg':S11P_leg}
S12U =  {'pen':S12U_pen,'mkr':S12U_mkr,'leg':S12U_leg,'sleg':S12U_leg}
S12P =  {'pen':S12P_pen,'mkr':S12P_mkr,'leg':S12P_leg,'sleg':S12P_leg}
S2P  =  {'pen':S2P_pen, 'mkr':S2P_mkr, 'leg':S2P_leg, 'sleg':S2P_leg}
S3U  =  {'pen':S3U_pen, 'mkr':S3U_mkr, 'leg':S3U_leg, 'sleg':S3U_leg}
S3P  =  {'pen':S3P_pen, 'mkr':S3P_mkr, 'leg':S3P_leg, 'sleg':S3P_leg}
S5U  =  {'pen':S5U_pen, 'mkr':S5U_mkr, 'leg':S5U_leg, 'sleg':S5U_leg}
S5P  =  {'pen':S5P_pen, 'mkr':S5P_mkr, 'leg':S5P_leg, 'sleg':S5P_leg}
ST1  =  {'pen':ST1_pen, 'mkr':ST1_mkr, 'leg':ST1_leg, 'sleg':ST1_leg}
ST1R =  {'pen':ST1_pen, 'mkr':ST1_mkr, 'leg':ST1_leg, 'sleg':ST1_leg}


# columns
string_col =       0
twist_angle_col =  1
test_type_col =    2
stretch_rate_col = 3
strain_rate_col =  4
br_stress_col =    5
br_strain_col =    6
br_E_col =         7

start_row = 10
br_string_list = []
string_list_C = []
angle_list_C = []
stress_list_C = []
strain_list_C = []
E_list_C = []

if(loaded_twist_set and loaded_surface_set):
    # connect to data source
    file_location = "./stretching summary.xls"
    workbook1 = xlrd.open_workbook(file_location)
    data_sheet = workbook1.sheet_by_index(0)
    row_count = data_sheet.nrows
    row = start_row
    while(row < row_count):
        if(data_sheet.cell(row,string_col).ctype in [0,6]):
            row += 1  # skip this row
        else:
            # next string
            string = data_sheet.cell_value(row,string_col)
            br_string_list.append(string)
            sdict = globals()[string]
            sdict['twist_angle'] = data_sheet.cell_value(row, twist_angle_col) \
                                   if(data_sheet.cell(row, twist_angle_col).ctype not in [0,6]) else np.nan
            # count number of records for this string
            count = 0
            while(((row + count) < row_count) and (data_sheet.cell(row+count,test_type_col).ctype not in [0,6])):
                count += 1
            # load full arrays
            sdict['test_type'] = [data_sheet.cell_value(i, test_type_col) for i in range(row, row + count)]
            sdict['stretch_rate'] = np.array([data_sheet.cell_value(i, stretch_rate_col) 
                                              if(data_sheet.cell(i, stretch_rate_col).ctype not in [0,6]) 
                                              else np.nan for i in range(row, row + count)])
            sdict['strain_rate'] = np.array([data_sheet.cell_value(i, strain_rate_col) for i in range(row, row + count)])
            sdict['br_stress'] = np.array([data_sheet.cell_value(i, br_stress_col) for i in range(row, row + count)])
            sdict['br_strain'] = np.array([data_sheet.cell_value(i, br_strain_col) 
                                           if(data_sheet.cell(i, br_strain_col).ctype not in [0,6]) 
                                           else np.nan for i in range(row, row + count)])
            sdict['br_E'] = np.array([data_sheet.cell_value(i, br_E_col) 
                                      if(data_sheet.cell(i, br_E_col).ctype not in [0,6]) 
                                      else np.nan for i in range(row, row + count)])
            # separate out results from Bowbrand tests and Instron tests at 3 mm/min
            found_B = False
            found_C3 = False
            for i in range(count):
                # Bowbrand test results
                if(sdict['test_type'][i] == 'B'):
                    sdict['br_stress_B'] = sdict['br_stress'][i]
                    found_B = True
                # Instron test results at 3 mm/min
                if((sdict['test_type'][i] == 'C') and (sdict['stretch_rate'][i] == 3)):
                    sdict['br_stress_C'] = sdict['br_stress'][i]
                    sdict['br_strain_C'] = sdict['br_strain'][i]
                    sdict['br_E_C'] = sdict['br_E'][i]
                    string_list_C.append(string)
                    angle_list_C.append(sdict['twist_angle'])
                    stress_list_C.append(sdict['br_stress_C'])
                    strain_list_C.append(sdict['br_strain_C'])
                    E_list_C.append(sdict['br_E_C'])
                    found_C3 = True
            if(not found_B):
                sdict['br_stress_B'] = np.nan
            if(not found_C3):
                sdict['br_stress_C'] = np.nan
                sdict['br_strain_C'] = np.nan
                sdict['br_E_C'] = np.nan
            print(string, count)
            row += count
else:
    print('\n**** Load other summary data sets first\n')

print('\n',br_string_list,'\n')

for i in range(len(string_list_C)):
     print(string_list_C[i], angle_list_C[i], stress_list_C[i])
print('\n',string_list_C,'\n')
```

```
G1 3
G2 4
T1A 2
T1B 2
T2A 3
T2B 3
T3A 6
T3B 2
T4A 1
T4B 3
S1U 1
S1P 3
S11U 1
S11P 2
S12U 1
S12P 1
S2P 1
S3U 1
S3P 1
S5U 1
S5P 1
ST1 1
ST1R 1

 ['G1', 'G2', 'T1A', 'T1B', 'T2A', 'T2B', 'T3A', 'T3B', 'T4A', 'T4B', 'S1U', 'S1P', 'S11U', 'S11P', 'S12U', 'S12P', 'S2P', 'S3U', 'S3P', 'S5U', 'S5P', 'ST1', 'ST1R'] 

T1A 36.192752460875795 277.0
T1B 35.18641337606319 276.0
T2A 36.24764448802202 209.0
T2B 29.261557454021343 268.0
T3A 23.531922623968303 331.0
T3B 28.672941656986204 279.0
T4B 33.92786032256368 211.0
S1U 30.658431381741376 260.0
S1P 30.658431381741376 341.0
S11U 31.15205575057074 279.0
S11P 31.15205575057074 218.0
S12U 29.242886678695452 283.0
S12P 29.242886678695452 272.0
S2P 28.310670291569 321.0
S3U 25.691985900870723 355.0
S3P 25.691985900870723 299.0
S5U 28.39248934731805 265.0
S5P 28.39248934731805 325.0
ST1 58.26355828049381 61.0
ST1R 58.26355828049381 70.0

 ['T1A', 'T1B', 'T2A', 'T2B', 'T3A', 'T3B', 'T4B', 'S1U', 'S1P', 'S11U', 'S11P', 'S12U', 'S12P', 'S2P', 'S3U', 'S3P', 'S5U', 'S5P', 'ST1', 'ST1R']
```

## Length adjustment offset comparison¶

In [6]:

```
fig=plt.figure(figsize=(12,3))
ax1=fig.add_subplot(131)
ax2=fig.add_subplot(132)
ax3=fig.add_subplot(133)
#
ax1.plot(offset_diff_value_A, 'bo', ms=4, label='Twist angle study')
ax1.plot(offset_diff_value_S, 'co', ms=4, label='Surface finish study')
ax1.set_xlabel('index')
ax1.set_ylabel('Offset difference (m)')
#
ax2.plot(offset_diff_angle_A, offset_diff_value_A, 'bo', ms=4)
ax2.plot(offset_diff_angle_S, offset_diff_value_S, 'co', ms=4)
ax2.set_xlabel('Twist angle (degrees)')
ax2.set_ylabel('Offset difference (m)')
#
ax3.plot(offset_diff_diameter_A, offset_diff_value_A, 'bo', ms=4, label='Twist angle study')
ax3.plot(offset_diff_diameter_S, offset_diff_value_S, 'co', ms=4, label='Surface finish study')
ax3.set_xlabel('Diameter (mm)')
ax3.set_ylabel('Offset difference (m)')
ax3.legend()
#
plt.tight_layout()
plt.show()
```

## Density ratio vs. strain¶

In [7]:

```
fig=plt.figure(figsize=(10,3.7))
ax1=fig.add_subplot(121)
ax2=fig.add_subplot(122)
axis_limits = [0, 0.14, 0.9, 1.02]

# ground strings
for string in (plot_list_A + ['S1Pa','S11Pa','S12Pa','S2Pa','S2Pb','S3Pa','S5Pa']):
    sdict = globals()[string]
    ax1.errorbar(x=sdict['strain0'], y=sdict['den_ratio'], yerr=[sdict['den_err_minus'], sdict['den_err_plus']], 
                 color=sdict['pen'], ls='-', lw=1, marker=sdict['mkr'], ms=4, elinewidth=1, capsize=3, label=sdict['sleg'])
handles, labels = ax1.get_legend_handles_labels()  # for avoiding duplicate labels
by_label = dict(zip(labels, handles))              # for avoiding duplicate labels
ax1.legend(by_label.values(), by_label.keys(), loc='lower right', ncol = 3, prop={'size':8}, framealpha=0)
ax1.axis(axis_limits)
ax1.set_xlabel('Strain')
ax1.set_ylabel('Density ratio')
ax1.set_title('Ground strings')
#
# unound strings
for string in (['S11Ua','S12Ua','S12Ub','S3Ua','S5Ua'] + ['ST1a','ST1b']):
    sdict = globals()[string]
    ax2.errorbar(x=sdict['strain0'], y=sdict['den_ratio'], yerr=[sdict['den_err_minus'], sdict['den_err_plus']], 
                 color=sdict['pen'], ls='-', lw=1, marker=sdict['mkr'], ms=4, elinewidth=1, capsize=3, label=sdict['sleg'])
handles, labels = ax2.get_legend_handles_labels()  # for avoiding duplicate labels
by_label = dict(zip(labels, handles))              # for avoiding duplicate labels
ax2.legend(by_label.values(), by_label.keys(), loc='lower right', ncol = 2, prop={'size':8}, framealpha=0)
ax2.axis(axis_limits)
ax2.set_xlabel('Strain')
ax2.set_ylabel('Density ratio')
ax2.set_title('Unground strings')

#
plt.tight_layout()
ax1.text(0.9, 0.9,'(a)', transform=ax1.transAxes)
ax2.text(0.9, 0.9,'(b)', transform=ax2.transAxes)
#plt.savefig('./density_ratio.eps', format='eps', dpi=1000)
plt.show()
```

In [8]:

```
fig=plt.figure(figsize=(8,7.1))
ax1=fig.add_subplot(221)
ax2=fig.add_subplot(222)
ax3=fig.add_subplot(223)
ax4=fig.add_subplot(224)
axis_limits = [0, 0.09, 0.87, 1.0]
#
# linear density ratio - ground strings
for string in ['S1Pa','S11Pa','S12Pa','S2Pa','S2Pb','S3Pa','S5Pa']:
    sdict = globals()[string]
    ax1.plot(sdict['strain0'], sdict['mu0']/sdict['mu0'][0], color=sdict['pen'], ls='-', lw=1, 
             marker=sdict['mkr'], ms=4, label=sdict['sleg'])
handles, labels = ax1.get_legend_handles_labels()  # for avoiding duplicate labels
by_label = dict(zip(labels, handles))              # for avoiding duplicate labels
ax1.legend(by_label.values(), by_label.keys(), loc='lower left', ncol = 2, prop={'size':8}, framealpha=0)
ax1.axis(axis_limits)
ax1.set_xlabel('Strain')
ax1.set_ylabel('Linear density ratio')
ax1.set_title('Surface finish study - ground strings')
#
# linear density ratio - unground strings
for string in ['S11Ua','S12Ua','S12Ub','S3Ua','S5Ua']:
    sdict = globals()[string]
    ax2.plot(sdict['strain0'], sdict['mu0']/sdict['mu0'][0], color=sdict['pen'], ls='-', lw=1, 
             marker=sdict['mkr'], ms=4, label=sdict['sleg'])
handles, labels = ax2.get_legend_handles_labels()  # for avoiding duplicate labels
by_label = dict(zip(labels, handles))              # for avoiding duplicate labels
ax2.legend(by_label.values(), by_label.keys(), loc='lower left', ncol = 2, prop={'size':8}, framealpha=0)
ax2.axis(axis_limits)
ax2.set_xlabel('Strain')
ax2.set_ylabel('Linear density ratio')
ax2.set_title('Surface finish study - unground strings')
#
# cross-sectional area ratio - ground strings
for string in ['S1Pa','S11Pa','S12Pa','S2Pa','S2Pb','S3Pa','S5Pa']:
    sdict = globals()[string]
    ax3.plot(sdict['strain0'], sdict['area0']/sdict['area0'][0], color=sdict['pen'], ls='-', lw=1, 
             marker=sdict['mkr'], ms=4, label=sdict['sleg'])
handles, labels = ax3.get_legend_handles_labels()  # for avoiding duplicate labels
by_label = dict(zip(labels, handles))              # for avoiding duplicate labels
ax3.legend(by_label.values(), by_label.keys(), loc='lower left', ncol = 2, prop={'size':8}, framealpha=0)
ax3.axis(axis_limits)
ax3.set_xlabel('Strain')
ax3.set_ylabel('Cross-sectional area ratio')
#ax3.set_title('Surface finish study - ground strings')
#
# cross-sectional area ratio - unground strings
for string in ['S11Ua','S12Ua','S12Ub','S3Ua','S5Ua']:
    sdict = globals()[string]
    ax4.plot(sdict['strain0'], sdict['area0']/sdict['area0'][0], color=sdict['pen'], ls='-', lw=1, 
             marker=sdict['mkr'], ms=4, label=sdict['sleg'])
handles, labels = ax4.get_legend_handles_labels()  # for avoiding duplicate labels
by_label = dict(zip(labels, handles))              # for avoiding duplicate labels
ax4.legend(by_label.values(), by_label.keys(), loc='lower left', ncol = 2, prop={'size':8}, framealpha=0)
ax4.axis(axis_limits)
ax4.set_xlabel('Strain')
ax4.set_ylabel('Cross-sectional area ratio')
#ax4.set_title('Surface finish study - unground strings')
#
plt.tight_layout()
ax1.text(0.9, 0.9,'(a)', transform=ax1.transAxes)
ax2.text(0.9, 0.9,'(b)', transform=ax2.transAxes)
ax3.text(0.9, 0.9,'(c)', transform=ax3.transAxes)
ax4.text(0.9, 0.9,'(d)', transform=ax4.transAxes)
#plt.savefig('./mu_and_area_ratios_S.eps', format='eps', dpi=1000)
plt.show()
```

## Breaking Stress¶

In [9]:

```
# breaking stress vs. strain rate
fig=plt.figure(figsize=(6,3.7))
ax1=fig.add_subplot(111)
for string in br_string_list:
    sdict = globals()[string]
    if(np.count_nonzero(~np.isnan(sdict['br_stress'])) > 1):
        str_label = sdict['sleg'][0:3] if(string[0] == 'T') else sdict['sleg']
        ax1.semilogx(sdict['strain_rate'], sdict['br_stress'], color=sdict['pen'], ls='none', marker=sdict['mkr'], ms=4, 
                     label=str_label)
        for i in range(len(sdict['br_stress'])-1):
            l_style = '-' if(sdict['test_type'][i] == sdict['test_type'][i+1]) else ':'
            ax1.semilogx([sdict['strain_rate'][i],sdict['strain_rate'][i+1]], [sdict['br_stress'][i],sdict['br_stress'][i+1]],
                         color=sdict['pen'], ls=l_style)
handles, labels = ax1.get_legend_handles_labels()  # for avoiding duplicate labels
by_label = dict(zip(labels, handles))              # for avoiding duplicate labels
ax1.legend(by_label.values(), by_label.keys(), loc='upper left', ncol = 2, prop={'size':8}, framealpha=0)
ax1.set_xlabel('Strain rate (/s)')
ax1.set_ylabel('Breaking stress (MPa)')
plt.tight_layout()  
#plt.savefig('./br_stress_strain_rate.eps', format='eps', dpi=1000)
plt.show()
    
# breaking strain vs. strain rate
fig=plt.figure(figsize=(6,3.7))
ax2=fig.add_subplot(111)
for string in br_string_list:
    sdict = globals()[string]
    if(np.count_nonzero(~np.isnan(sdict['br_strain'])) > 1):
        str_label = sdict['sleg'][0:3] if(string[0] == 'T') else sdict['sleg']
        ax2.semilogx(sdict['strain_rate'], sdict['br_strain'], color=sdict['pen'], ls='none', marker=sdict['mkr'], ms=4, 
                     label=str_label)
        for i in range(len(sdict['br_strain'])-1):
            l_style = '-' if(sdict['test_type'][i] == sdict['test_type'][i+1]) else ':'
            ax2.semilogx([sdict['strain_rate'][i],sdict['strain_rate'][i+1]], [sdict['br_strain'][i],sdict['br_strain'][i+1]],
                         color=sdict['pen'], ls=l_style)
handles, labels = ax2.get_legend_handles_labels()  # for avoiding duplicate labels
by_label = dict(zip(labels, handles))              # for avoiding duplicate labels
ax2.legend(by_label.values(), by_label.keys(), loc='upper left', ncol = 2, prop={'size':8}, framealpha=0)
ax2.set_xlabel('Strain rate (/s)')
ax2.set_ylabel('Breaking strain')
plt.tight_layout()  
#plt.savefig('./br_strain_strain_rate.eps', format='eps', dpi=1000)
plt.show()

# Young's modulus vs. strain rate
plt.figure(figsize=(6,3.7))
for string in br_string_list:
    sdict = globals()[string]
    if(np.count_nonzero(~np.isnan(sdict['br_E'])) > 1):
        str_label = sdict['sleg'][0:3] if(string[0] == 'T') else sdict['sleg']
        plt.semilogx(sdict['strain_rate'], sdict['br_E'], color=sdict['pen'], ls='-', marker=sdict['mkr'], ms=4, 
                     label=str_label)
    if(string == 'T3A'):
        plt.semilogx([sdict['strain_rate'][-1],sdict['strain_rate'][-3]], [sdict['br_E'][-1],sdict['br_E'][-3]],
                     color=sdict['pen'], ls='-')
plt.legend(loc='upper left', framealpha=0)
plt.xlabel('Strain rate (/s)')
plt.ylabel("Young's modulus (GPa)")
plt.tight_layout()  
#plt.savefig('./br_E_strain_rate.eps', format='eps', dpi=1000)
plt.show()
```

In [10]:

```
# string order in arrays is T1A T1B T2A T2B T3A T3B T4B S1U S1P S11U S11P S12U S12P S2P S3U S3P S5U S5P ST1 ST1R 
fig, (ax1, ax2) = plt.subplots(1, 2, gridspec_kw={'width_ratios': [2,1]}, figsize=(10,3.7))

# Breaking stress vs. twist angle
# fitted line
x_range = np.array([19,80])
fit_C = poly.polyfit(np.log10(np.array(angle_list_C[2:-2])), stress_list_C[2:-2], 1)  # excluding T1A, T1B, ST1, ST1R
print(fit_C)
print(10**(-fit_C[0]/fit_C[1]))
ax1.plot(x_range, poly.polyval(np.log10(x_range), fit_C), 'k--', lw=0.5)
# data from Bowbrand tests
ax1.plot([T1A['twist_angle'],T1B['twist_angle']], [T1A['br_stress_B'],T1B['br_stress_B']], color=T1_pen, lw=1, 
         marker='o', mec=T1_pen, mfc=T1_pen, mew=1, label=T1_leg)
ax1.plot([T2A['twist_angle'],T2B['twist_angle']], [T2A['br_stress_B'],T2B['br_stress_B']], color=T2_pen, lw=1, 
         marker='o', mec=T2_pen, mfc=T2_pen, mew=1, label=T2_leg)
ax1.plot([T3A['twist_angle'],T3B['twist_angle']], [T3A['br_stress_B'],T3B['br_stress_B']], color=T3_pen, lw=1, 
         marker='o', mec=T3_pen, mfc=T3_pen, mew=1, label=T3_leg)
ax1.plot([T4A['twist_angle'],T4B['twist_angle']], [T4A['br_stress_B'],T4B['br_stress_B']], color=T4_pen, lw=1, 
         marker='o', mec=T4_pen, mfc=T4_pen, mew=1, label=T4_leg)
# data from Cambridge Instron tests
ax1.plot([T1A['twist_angle'],T1B['twist_angle']], [T1A['br_stress_C'],T1B['br_stress_C']], color=T1_pen, lw=1, 
         marker='o', mec=T1_pen, mfc=T1_pen, mew=1)
ax1.plot([T2A['twist_angle'],T2B['twist_angle']], [T2A['br_stress_C'],T2B['br_stress_C']], color=T2_pen, lw=1, 
         marker='o', mec=T2_pen, mfc=T2_pen, mew=1)
ax1.plot([T3A['twist_angle'],T3B['twist_angle']], [T3A['br_stress_C'],T3B['br_stress_C']], color=T3_pen, lw=1, 
         marker='o', mec=T3_pen, mfc=T3_pen, mew=1)
ax1.plot([T4A['twist_angle'],T4B['twist_angle']], [T4A['br_stress_C'],T4B['br_stress_C']], color=T4_pen, lw=1, 
         marker='o', mec=T4_pen, mfc=T4_pen, mew=1)
for string in ['S1U','S1P','S11U','S11P','S12U','S12P','S2P','S3U','S3P','S5U','S5P','ST1','ST1R']:
    sdict = globals()[string]
    ax1.plot(sdict['twist_angle'], sdict['br_stress_C'], color=sdict['pen'], marker=sdict['mkr'], ms=4, 
                     label=sdict['sleg'])
# Bell & Firth
ax1.errorbar(bf_angle, bf_br_strength, yerr=bf_br_strength_SD, ls='none', marker='o', ms=4, mec='magenta', mfc='magenta', 
             ecolor='magenta', capsize=3, elinewidth=1, label='Bell & Firth')
# axes
handles, labels = ax1.get_legend_handles_labels()  # for avoiding duplicate labels
by_label = dict(zip(labels, handles))              # for avoiding duplicate labels
ax1.legend(by_label.values(), by_label.keys(), loc='upper right', bbox_to_anchor=(0, 0, 1, 0.98), ncol=2, prop={'size':8}, 
           framealpha=0)
ax1.axis([19, 90, 0, 680])
ax1.set_xscale("log")
x_axis = np.arange(20,100,10)
x_values = [20,30,40,50,60,70,80,90]
ax1.set_xticks(x_axis, x_values)
ax1.set_xlabel('Twist angle')
ax1.set_ylabel('Breaking stress (MPa)')
ax1.set_title('All strings plus strings from previous studies')
ax1.xaxis.set_major_formatter(ticker.StrMethodFormatter(u"{x:.0f}°"))
#
# ============================================================
# compare breaking stress for ground and unground strings
#ax2.plot([-2,2],[0,0],'k--',lw=1)  # Zero reference
ax2.plot([0,0],[0, 800],'k:',lw=1)
ax2.plot([1,1],[0, 800],'k:',lw=1)
# connecting lines
ax2.plot([0,1], [S11P['br_stress_C'],S11U['br_stress_C']], 'k--', lw=0.5)   
ax2.plot([0,1], [S12P['br_stress_C'],S12U['br_stress_C']], 'k--', lw=0.5)   
ax2.plot([0,1], [S3P['br_stress_C'],S3U['br_stress_C']], 'k--', lw=0.5)   
ax2.plot([0,1], [S5P['br_stress_C'],S5U['br_stress_C']], 'k--', lw=0.5)   
# ground strings
for string in ['S1P','S11P','S12P','S2P','S3P','S5P']:
    sdict = globals()[string]
    ax2.plot(0, sdict['br_stress_C'], color=sdict['pen'], marker=sdict['mkr'], ms=4)
for string in ['S11U','S12U','S3U','S5U']:
    sdict = globals()[string]
    ax2.plot(1, sdict['br_stress_C'], color=sdict['pen'], marker=sdict['mkr'], ms=4)
ax2.axis([-0.2, 1.2, 0, 680])
x_axis = np.arange(2)
x_values = ['ground', 'unground']
ax2.set_xticks(x_axis, x_values)
ax2.set_xlabel('Surface finish')
ax2.set_ylabel('Breaking stress (MPa)')
ax2.set_title('Ground vs. unground')
#
plt.tight_layout()
plt.subplots_adjust(wspace=0.2)
ax1.text(0.47, 0.92,'(a)', transform=ax1.transAxes)
ax2.text(0.45, 0.92,'(b)', transform=ax2.transAxes)
#plt.savefig('./breaking_stress.eps', format='eps', dpi=1000)
plt.show()
```

```
[1369.18007137 -741.44688896]
70.2477957183505
```

In [11]:

```
# string order in arrays is T1A T1B T2A T2B T3A T3B T4B S1U S1P S11U S11P S12U S12P S2P S3U S3P S5U S5P ST1 ST1R 
fig, (ax1, ax2) = plt.subplots(1, 2, gridspec_kw={'width_ratios': [2,1]}, figsize=(10,3.7))

# Breaking strain vs. twist angle
# data from Cambridge Instron tests
ax1.plot([T1A['twist_angle'],T1B['twist_angle']], [T1A['br_strain_C'],T1B['br_strain_C']], color=T1_pen, lw=1, 
         marker='o', mec=T1_pen, mfc=T1_pen, mew=1, label=T1_leg)
ax1.plot([T2A['twist_angle'],T2B['twist_angle']], [T2A['br_strain_C'],T2B['br_strain_C']], color=T2_pen, lw=1, 
         marker='o', mec=T2_pen, mfc=T2_pen, mew=1, label=T2_leg)
ax1.plot([T3A['twist_angle'],T3B['twist_angle']], [T3A['br_strain_C'],T3B['br_strain_C']], color=T3_pen, lw=1, 
         marker='o', mec=T3_pen, mfc=T3_pen, mew=1, label=T3_leg)
ax1.plot([T4A['twist_angle'],T4B['twist_angle']], [T4A['br_strain_C'],T4B['br_strain_C']], color=T4_pen, lw=1, 
         marker='o', mec=T4_pen, mfc=T4_pen, mew=1, label=T4_leg)
for string in ['S1U','S1P','S11U','S11P','S12U','S12P','S2P','S3U','S3P','S5U','S5P','ST1','ST1R']:
    sdict = globals()[string]
    ax1.plot(sdict['twist_angle'], sdict['br_strain_C'], color=sdict['pen'], marker=sdict['mkr'], ms=4, 
                     label=sdict['sleg'])
# axes
handles, labels = ax1.get_legend_handles_labels()  # for avoiding duplicate labels
by_label = dict(zip(labels, handles))              # for avoiding duplicate labels
ax1.legend(by_label.values(), by_label.keys(), loc='upper right', bbox_to_anchor=(0, 0, 1, 0.98), ncol=2, prop={'size':8}, 
           framealpha=0)
ax1.axis([19, 90, 0.1, 0.33])
ax1.set_xscale("log")
x_axis = np.arange(20,100,10)
x_values = [20,30,40,50,60,70,80,90]
ax1.set_xticks(x_axis, x_values)
ax1.set_xlabel('Twist angle')
ax1.set_ylabel('Breaking strain')
ax1.set_title('All strings plus strings from previous studies')
ax1.xaxis.set_major_formatter(ticker.StrMethodFormatter(u"{x:.0f}°"))
#
# ============================================================
# compare breaking stress for ground and unground strings
#ax2.plot([-2,2],[0,0],'k--',lw=1)  # Zero reference
ax2.plot([0,0],[0, 800],'k:',lw=1)
ax2.plot([1,1],[0, 800],'k:',lw=1)
# connecting lines
ax2.plot([0,1], [S11P['br_strain_C'],S11U['br_strain_C']], 'k--', lw=0.5)   
ax2.plot([0,1], [S12P['br_strain_C'],S12U['br_strain_C']], 'k--', lw=0.5)   
ax2.plot([0,1], [S3P['br_strain_C'],S3U['br_strain_C']], 'k--', lw=0.5)   
ax2.plot([0,1], [S5P['br_strain_C'],S5U['br_strain_C']], 'k--', lw=0.5)   
# ground strings
for string in ['S1P','S11P','S12P','S2P','S3P','S5P']:
    sdict = globals()[string]
    ax2.plot(0, sdict['br_strain_C'], color=sdict['pen'], marker=sdict['mkr'], ms=4)
for string in ['S11U','S12U','S3U','S5U']:
    sdict = globals()[string]
    ax2.plot(1, sdict['br_strain_C'], color=sdict['pen'], marker=sdict['mkr'], ms=4)
ax2.axis([-0.2, 1.2, 0.1, 0.33])
x_axis = np.arange(2)
x_values = ['ground', 'unground']
ax2.set_xticks(x_axis, x_values)
ax2.set_xlabel('Surface finish')
ax2.set_ylabel('Breaking strain')
ax2.set_title('Ground vs. unground')
#
plt.tight_layout()
plt.subplots_adjust(wspace=0.2)
ax1.text(0.47, 0.92,'(a)', transform=ax1.transAxes)
ax2.text(0.45, 0.92,'(b)', transform=ax2.transAxes)
#plt.savefig('./breaking_strain.eps', format='eps', dpi=1000)
plt.show()
```

## Stress vs. strain¶

In [12]:

```
fig=plt.figure(figsize=(8,3.7))
ax1=fig.add_subplot(121)
ax2=fig.add_subplot(122)
axis_limits = [0, 0.15, 0, 145]

for string in plot_list_A:
    sdict = globals()[string]
    ax1.plot(sdict['strain0'], sdict['stress0'], color=sdict['pen'], ls='-', lw=1, marker=sdict['mkr'], ms=4, 
             label=sdict['sleg'])
handles, labels = ax1.get_legend_handles_labels()  # for avoiding duplicate labels
by_label = dict(zip(labels, handles))              # for avoiding duplicate labels
ax1.legend(by_label.values(), by_label.keys(), loc='lower right', ncol = 1, prop={'size':8}, framealpha=0)
ax1.axis(axis_limits)
ax1.set_xlabel('Strain')
ax1.set_ylabel('Stress (MPa)')
ax1.set_title('Twist angle study')
#
for string in plot_list_S:
    sdict = globals()[string]
    ax2.plot(sdict['strain0'], sdict['stress0'], color=sdict['pen'], ls='-', lw=1, marker=sdict['mkr'], ms=4, label=sdict['sleg'])
handles, labels = ax2.get_legend_handles_labels()  # for avoiding duplicate labels
by_label = dict(zip(labels, handles))              # for avoiding duplicate labels
ax2.legend(by_label.values(), by_label.keys(), loc='lower right', ncol = 1, prop={'size':8}, framealpha=0)
ax2.axis(axis_limits)
ax2.set_xlabel('Strain')
ax2.set_ylabel('Stress (MPa)')
ax2.set_title('Surface finish study')
#
plt.tight_layout()
ax1.text(0.05, 0.9,'(a)', transform=ax1.transAxes)
ax2.text(0.05, 0.9,'(b)', transform=ax2.transAxes)
#plt.savefig('./stress_strain.eps', format='eps', dpi=1000)
plt.show()
```

## Stress vs. strain using alternate length adjustment offset correction¶

In [13]:

```
fig=plt.figure(figsize=(8,3.7))
ax1=fig.add_subplot(121)
ax2=fig.add_subplot(122)
axis_limits = [0, 0.15, 0, 145]

for string in plot_list_A:
    sdict = globals()[string]
    ax1.plot(sdict['alt_strain0'], sdict['stress0'], color=sdict['pen'], ls='-', lw=1, marker=sdict['mkr'], ms=4, label=sdict['sleg'])
handles, labels = ax1.get_legend_handles_labels()  # for avoiding duplicate labels
by_label = dict(zip(labels, handles))              # for avoiding duplicate labels
ax1.legend(by_label.values(), by_label.keys(), loc='lower right', ncol = 1, prop={'size':8}, framealpha=0)
ax1.axis(axis_limits)
ax1.set_facecolor('silver')
ax1.set_xlabel('Strain using alternate length adj. correction')
ax1.set_ylabel('Stress (MPa)')
ax1.set_title('Twist angle study')
#
for string in plot_list_S:
    sdict = globals()[string]
    ax2.plot(sdict['alt_strain0'], sdict['stress0'], color=sdict['pen'], ls='-', lw=1, marker=sdict['mkr'], ms=4, label=sdict['sleg'])
handles, labels = ax2.get_legend_handles_labels()  # for avoiding duplicate labels
by_label = dict(zip(labels, handles))              # for avoiding duplicate labels
ax2.legend(by_label.values(), by_label.keys(), loc='lower right', ncol = 1, prop={'size':8}, framealpha=0)
ax2.axis(axis_limits)
ax2.set_facecolor('silver')
ax2.set_xlabel('Strain using alternate length adj. correction')
ax2.set_ylabel('Stress (MPa)')
ax2.set_title('Surface finish study')
#
plt.tight_layout()
ax1.text(0.05, 0.9,'(a)', transform=ax1.transAxes)
ax2.text(0.05, 0.9,'(b)', transform=ax2.transAxes)
#plt.savefig('./stress_strain_alt.eps', format='eps', dpi=1000)
plt.show()
```

## Young's modulus vs. stress & twist angle¶

In [14]:

```
# array sequence is T1A T1B T2A T2B T3A T3B T4A T4B S11U S1P S11P S12U S12P S2P S3U S3P S5U S5P ST1
assemble_avg_arrays()

fig=plt.figure(figsize=(10,3.7))
ax1=fig.add_subplot(121)
ax2=fig.add_subplot(122)

# Young's modulus versus stress - data from surface finish study
print('Value ranges:')
for string in plot_list_A:
    sdict = globals()[string]
    ax1.plot(sdict['Es_stress'], sdict['Es'], color=sdict['pen'], ls='-', lw=1, marker='*')
    ax1.plot(sdict['stress'], sdict['Et'], color=sdict['pen'], ls='-', lw=1, marker='+') 
    ax1.plot(sdict['stress'], sdict['Eb'], color=sdict['pen'], lw=1, marker='o', ms=4, mec=sdict['pen'], mfc='none', mew=1)
    print('%5s: Es: %.2f, Et: %.2f, Eb: %.2f' % (string, (max(sdict['Es'])-min(sdict['Es'])), 
                                                 (max(sdict['Et'])-min(sdict['Et'])), (max(sdict['Eb'])-min(sdict['Eb']))))
    # plain lines for legend
    ax1.plot([-2,-1], [-2,-1], color=sdict['pen'], ls='-', lw=1, label=sdict['sleg'])
handles, labels = ax1.get_legend_handles_labels()  # for avoiding duplicate labels
by_label = dict(zip(labels, handles))              # for avoiding duplicate labels
ax1.legend(by_label.values(), by_label.keys(), loc='upper left', ncol = 1, prop={'size':8}, framealpha=0)
ax1.axis([0, 145, 0, 9])
ax1.set_xlabel('Stress (MPa)')
ax1.set_ylabel("Young's modulus (GPa)")
ax1.set_title('Twist angle study')
#
# ============================================================
# Young's modulus versus stress - data from surface finish study
for string in plot_list_S:
    sdict = globals()[string]
    ax2.plot(sdict['Es_stress'], sdict['Es'], color=sdict['pen'], ls='-', lw=1, marker='*')
    ax2.plot(sdict['stress'], sdict['Et'], color=sdict['pen'], ls='-', lw=1, marker='+') 
    ax2.plot(sdict['stress'], sdict['Eb'], color=sdict['pen'], lw=1, marker='o', ms=4, mec=sdict['pen'], mfc='none', mew=1) 
    # plain lines for legend
    ax2.plot([-2,-1], [-2,-1], color=sdict['pen'], ls='-', lw=1, label=sdict['sleg'])
handles, labels = ax2.get_legend_handles_labels()  # for avoiding duplicate labels
by_label = dict(zip(labels, handles))              # for avoiding duplicate labels
ax2.legend(by_label.values(), by_label.keys(), loc='upper left', ncol = 1, prop={'size':8}, framealpha=0)
ax2.axis([0, 145, 0, 9])
ax2.set_xlabel('Stress (MPa)')
ax2.set_ylabel("Young's modulus (GPa)")
ax2.set_title('Surface finish study')
#
plt.tight_layout()
ax1.text(0.9, 0.9,'(a)', transform=ax1.transAxes)
ax2.text(0.9, 0.9,'(b)', transform=ax2.transAxes)
#plt.savefig('./E_vs_stress.eps', format='eps', dpi=1000)
plt.show()
```

```
Value ranges:
 T1Aa: Es: 0.25, Et: 0.29, Eb: 0.49
 T2Ab: Es: 0.01, Et: 0.00, Eb: 0.72
 T1Ba: Es: 0.19, Et: 0.21, Eb: 0.39
 T4Ba: Es: 0.70, Et: 0.68, Eb: 0.43
 T4Bb: Es: 0.38, Et: 0.37, Eb: 0.22
 T2Ba: Es: 0.28, Et: 0.12, Eb: 0.10
 T2Bb: Es: 0.35, Et: 0.20, Eb: 0.84
 T3Ba: Es: 0.35, Et: 0.40, Eb: 0.32
 T3Bb: Es: 0.31, Et: 0.30, Eb: 0.56
 T4Ac: Es: 0.34, Et: 0.37, Eb: 0.93
 T3Aa: Es: 0.26, Et: 0.16, Eb: 0.23
```

In [15]:

```
from sklearn.linear_model import LinearRegression
lm = LinearRegression(fit_intercept = False)

# array sequence is T1A T1B T2A T2B T3A T3B T4A T4B S11U S1P S11P S12U S12P S2P S3U S3P S5U S5P ST1
assemble_avg_arrays()

fig, (ax1, ax2) = plt.subplots(1, 2, gridspec_kw={'width_ratios': [5,2]}, figsize=(10,3.7))

# ============================================================
# Young's modulus vs. twist angle
x_plot_range = np.array([10,90])
x_fit_range = np.log10(x_plot_range) - np.log10(90)
#
# data from Cambridge Instron tests
x_data = np.log10(np.array(angle_list_C[:-2])) - np.log10(90)  # excluding ST1, ST1R
E_data = np.array(E_list_C[:-2])
lm.fit(x_data.reshape(-1, 1), E_data)
E_preds = lm.predict(x_fit_range.reshape(-1,1))
ax1.plot(x_plot_range, E_preds, 'k--', lw=0.5)
#
ax1.plot([T1A['twist_angle'],T1B['twist_angle']], [T1A['br_E_C'],T1B['br_E_C']], color=T1_pen, lw=1, 
         marker='x', mec=T1_pen, mfc=T1_pen, mew=1)
ax1.plot([T2A['twist_angle'],T2B['twist_angle']], [T2A['br_E_C'],T2B['br_E_C']], color=T2_pen, lw=1, 
         marker='x', mec=T2_pen, mfc=T2_pen, mew=1)
ax1.plot([T3A['twist_angle'],T3B['twist_angle']], [T3A['br_E_C'],T3B['br_E_C']], color=T3_pen, lw=1, 
         marker='x', mec=T3_pen, mfc=T3_pen, mew=1)
ax1.plot([T4A['twist_angle'],T4B['twist_angle']], [T4A['br_E_C'],T4B['br_E_C']], color=T4_pen, lw=1, 
         marker='x', mec=T4_pen, mfc=T4_pen, mew=1)
for string in ['S1U','S1P','S11U','S11P','S12U','S12P','S2P','S3U','S3P','S5U','S5P','ST1','ST1R']:
    sdict = globals()[string]
    ax1.plot(sdict['twist_angle'], sdict['br_E_C'], color=sdict['pen'], marker='x', ms=4)
#
# fitted lines
x_data = np.log10(np.array(avg_angle+gut_angle)) - np.log10(90)
Es_data = np.array(avg_Es + gut_Es)
Et_data = np.array(avg_Et + gut_Et)
Eb_data = np.array(avg_Eb + gut_Eb)
# Es
lm.fit(x_data.reshape(-1, 1), Es_data)
Es_preds = lm.predict(x_fit_range.reshape(-1,1))
ax1.plot(x_plot_range, Es_preds, 'k--', lw=0.5)
# Et
lm.fit(x_data.reshape(-1, 1), Et_data)
Et_preds = lm.predict(x_fit_range.reshape(-1,1))
ax1.plot(x_plot_range, Et_preds, 'k--', lw=0.5)
# Eb
lm.fit(x_data.reshape(-1, 1), Eb_data)
Eb_preds = lm.predict(x_fit_range.reshape(-1,1))
ax1.plot(x_plot_range, Eb_preds, 'k--', lw=0.5)
#
# data from twist angle study
for i in range(4):
    j = 2*i
    ax1.plot(avg_angle[j:j+2], avg_Es[j:j+2], color=avg_pen[j], lw=1, marker='*', mec=avg_pen[j], mew=1)
    ax1.plot(avg_angle[j:j+2], avg_Et[j:j+2], color=avg_pen[j], lw=1, marker='+', mec=avg_pen[j], mew=1, label=avg_leg[j])
    ax1.plot(avg_angle[j:j+2], avg_Eb[j:j+2], color=avg_pen[j], lw=1, marker='o', mec=avg_pen[j], mew=1, mfc='none')
# data from surface finish study
for index in range(8,len(avg_angle)):
    ax1.plot(avg_angle[index], avg_Es[index], color=avg_pen[index], marker='*', mec=avg_pen[index], mew=1)
    ax1.plot(avg_angle[index], avg_Et[index], color=avg_pen[index], marker='+', mec=avg_pen[index], mew=1, label=avg_leg[index])
    ax1.plot(avg_angle[index], avg_Eb[index], color=avg_pen[index], marker='o', mec=avg_pen[index], mew=1, mfc='none')
# older gut data
ax1.plot(gut_angle, gut_Es, color=gut_pen, ls='none', marker='*', mec=gut_pen, mew=1)
ax1.plot(gut_angle, gut_Et, color=gut_pen, ls='none', marker='+', mec=gut_pen, mew=1, label=gut_leg)
ax1.plot(gut_angle, gut_Eb, color=gut_pen, ls='none', marker='o', mec=gut_pen, mew=1, mfc='none')
ax1.plot([gut_angle[0],gut_angle[0]],[1,7.3],'g:',lw=0.5)
ax1.plot([gut_angle[1],gut_angle[1]],[1,7.3],'g:',lw=0.5)
ax1.plot([gut_angle[2],gut_angle[2]],[1,7.3],'g:',lw=0.5)
ax1.annotate("G2", xy=(gut_angle[0], 0.9), xytext=(gut_angle[0]-0.4, 0.1), arrowprops=dict(arrowstyle="-|>"), fontsize=8)
ax1.annotate("G3", xy=(gut_angle[1], 0.9), xytext=(gut_angle[1]-0.8, 0.1), arrowprops=dict(arrowstyle="-|>"), fontsize=8)
ax1.annotate("G5", xy=(gut_angle[2], 0.9), xytext=(gut_angle[2]-0.15, 0.1), arrowprops=dict(arrowstyle="-|>"), fontsize=8)
# Bell & Firth
ax1.errorbar(bf_angle, bf_Et, yerr=bf_Et_SD, ls='none', marker='o', ms=4, mec='magenta', mfc='magenta', ecolor='magenta', 
             capsize=3, elinewidth=1, label='Bell & Firth')
# lines for extra labels
ax1.plot([0,0],[0,0],ls='none',label='  ')
ax1.plot([0,0],[0,0],'ko',mew=1,mfc='none',label='$E_B$ values')
ax1.plot([0,0],[0,0],'k+',mew=1,label='$E_T$ values')
ax1.plot([0,0],[0,0],'k*',mew=1,label='$E_S$ values')
ax1.plot([0,0],[0,0],'kx',mew=1,label='Instron tests')
# legend
handles, labels = ax1.get_legend_handles_labels()  # for avoiding duplicate labels
handles.insert(-6, handles[-1])                    # adjust order
labels.insert(-6, labels[-1])                      # adjust order
by_label = dict(zip(labels[:-1], handles[:-1]))    # for avoiding duplicate labels
ax1.legend(by_label.values(), by_label.keys(), loc='upper right', bbox_to_anchor=(0, 0, 1, 0.98), 
           ncol=3, prop={'size':8}, framealpha=0)
# axes
ax1.axis([18, 90, 0, 7.5])
ax1.set_xscale("log")
x_axis = np.arange(20,100,10)
x_values = [20,30,40,50,60,70,80,90]
ax1.set_xticks(x_axis, x_values)
ax1.set_xlabel('Twist angle')
ax1.set_ylabel("Young's modulus (GPa)")
ax1.set_title('All strings plus strings from previous studies')
ax1.xaxis.set_major_formatter(ticker.StrMethodFormatter(u"{x:.0f}°"))
#
# ============================================================
# compare Young's modulus for ground and unground strings
ax2.plot([0,0],[0.5,7],'k:',lw=1)
ax2.plot([1,1],[0.5,7],'k:',lw=1)
#
# data from Cambridge Instron tests
# connecting lines
ax2.plot([0,1], [S11P['br_E_C'],S11U['br_E_C']], 'k--', lw=0.5)   
ax2.plot([0,1], [S12P['br_E_C'],S12U['br_E_C']], 'k--', lw=0.5)   
ax2.plot([0,1], [S3P['br_E_C'],S3U['br_E_C']], 'k--', lw=0.5)   
ax2.plot([0,1], [S5P['br_E_C'],S5U['br_E_C']], 'k--', lw=0.5)   
# ground strings
for string in ['S1P','S11P','S12P','S2P','S3P','S5P']:
    sdict = globals()[string]
    ax2.plot(0, sdict['br_E_C'], color=sdict['pen'], marker='x', ms=4)
for string in ['S11U','S12U','S3U','S5U']:
    sdict = globals()[string]
    ax2.plot(1, sdict['br_E_C'], color=sdict['pen'], marker='x', ms=4)
#
# array sequence is T1A T1B T2A T2B T3A T3B T4A T4B S11U S1P S11P S12U S12P S2P S3U S3P S5U S5P ST1
# indeces:           0   1   2   3   4   5   6   7   8    9   10   11   12   13  14  15  16  17  18
# Es
ax2.plot([1,0], [avg_Es[8],avg_Es[10]], 'k--', lw=0.5)   
ax2.plot([1,0], [avg_Es[11],avg_Es[12]], 'k--', lw=0.5)     
ax2.plot([1,0], [avg_Es[14],avg_Es[15]], 'k--', lw=0.5)     
ax2.plot([1,0], [avg_Es[16],avg_Es[17]], 'k--', lw=0.5)      
# Et
ax2.plot([1,0], [avg_Et[8],avg_Et[10]], 'k--', lw=0.5)       
ax2.plot([1,0], [avg_Et[11],avg_Et[12]], 'k--', lw=0.5)       
ax2.plot([1,0], [avg_Et[14],avg_Et[15]], 'k--', lw=0.5)       
ax2.plot([1,0], [avg_Et[16],avg_Et[17]], 'k--', lw=0.5)       
# Eb
ax2.plot([1,0], [avg_Eb[8],avg_Eb[10]], 'k--', lw=0.5)       
ax2.plot([1,0], [avg_Eb[11],avg_Eb[12]], 'k--', lw=0.5)       
ax2.plot([1,0], [avg_Eb[14],avg_Eb[15]], 'k--', lw=0.5)       
ax2.plot([1,0], [avg_Eb[16],avg_Eb[17]], 'k--', lw=0.5)      
#
for index in [9,10,12,13,15,17]:
    ax2.plot(0, avg_Es[index], color=avg_pen[index], marker='*', mec=avg_pen[index], mew=1.5)
    ax2.plot(0, avg_Et[index], color=avg_pen[index], marker='+', mec=avg_pen[index], mew=1.5)
    ax2.plot(0, avg_Eb[index], color=avg_pen[index], marker='o', mec=avg_pen[index], mew=1.5, mfc='none')
for index in [8,11,14,16]:
    ax2.plot(1, avg_Es[index], color=avg_pen[index], marker='*', mec=avg_pen[index], mew=1.5)
    ax2.plot(1, avg_Et[index], color=avg_pen[index], marker='+', mec=avg_pen[index], mew=1.5)
    ax2.plot(1, avg_Eb[index], color=avg_pen[index], marker='o', mec=avg_pen[index], mew=1.5, mfc='none')
ax2.axis([-0.2, 1.2, 0, 7.5])
x_axis = np.arange(2)
x_values = ['ground', 'unground']
ax2.set_xticks(x_axis, x_values)
ax2.set_xlabel('Surface finish')
ax2.set_ylabel("Young's modulus (GPa)")
ax2.set_title('Ground vs. unground')
#
plt.tight_layout()
ax1.text(0.47, 0.92,'(a)', transform=ax1.transAxes)
ax2.text(0.45, 0.92,'(b)', transform=ax2.transAxes)
#plt.savefig('./E_logx90_I.eps', format='eps', dpi=1000)
plt.show()
```

## $df/dT|\_L$ (constant length) & $df/dT|\_F$ (constant tension)¶

In [16]:

```
fig=plt.figure(figsize=(10,3.5))
ax1=fig.add_subplot(131)
ax2=fig.add_subplot(132)
ax3=fig.add_subplot(133)
#
# plots against 1/stress
axis_limits = [0.005, 0.047, -5, 2]
x_plot = np.arange(5.0, 300.0, 5.0)
#
ax1.plot([0,0.1],[0,0],'k--',lw=0.5)  # Zero reference
ax2.plot([0,70], [0,0],'k--',lw=0.5)  # Zero reference
ax3.plot([0,0.1],[0,0],'k--',lw=0.5)  # Zero reference
#
# df/dT at constant length 
for string in (plot_list_A + plot_list_S):
    sdict = globals()[string]
    ax1.plot(sdict['inv_stress'], sdict['dfdT_L'], color=sdict['pen'], ls='none', marker=sdict['mkr'], ms=3, 
             label=sdict['sleg'])
    if(sdict['points'] > 1):
        # fitted expression
        x_data = sdict['inv_stress']
        y_data = sdict['dfdT_L']
        sdict['dfdT_L_fit'] = poly.polyfit(x_data, y_data, 1)  # note coefficients now in increasing order
        ax1.plot(1/x_plot, poly.polyval(1/x_plot, sdict['dfdT_L_fit']), color=sdict['pen'], ls='--', lw=1)
        print("%s fit: offset: %9.6f,  slope: %11.6f,  rsq: %.6f" % 
              (string, sdict['dfdT_L_fit'][0], sdict['dfdT_L_fit'][1], 
               r_squared(y_data, poly.polyval(x_data, sdict['dfdT_L_fit']))))
ax1.axis(axis_limits)
ax1.set_xlabel('1/Stress (MPa$^{-1}$)')
ax1.set_ylabel('$df/dT$ (cent/°C)')
ax1.set_title('Constant length')
#
# df/dT at constant length versus twist angle
# array sequence is T1A T1B T2A T2B T3A T3B T4A T4B S11U S1P S11P S12U S12P S2P S3U S3P S5U S5P ST1
markers = ['s','o']
#
for index in [0,1]:
    # data from twist angle study
    for i in range(4):
        j = 2*i
        ax2.plot([avg_angle[j],avg_angle[j+1]], [avg_dfdT_L[j][index],avg_dfdT_L[j+1][index]], 
                 color=avg_pen[j], lw=1, marker=markers[index], mec=avg_pen[j], mfc=avg_pen[j], ms=3, mew=1)
    # data from surface finish study
    for j in range(8,len(avg_angle)-3):
        ax2.plot(avg_angle[j], avg_dfdT_L[j][index], color=avg_pen[j], marker=markers[index], 
                 mec=avg_pen[j], mfc=avg_pen[j], ms=3, mew=1)
#
# plain lines for legend
ax2.plot([-2,-1], [-2,-1], color=avg_pen[0], ls='-', lw=1, label=avg_leg[0])
ax2.plot([-2,-1], [-2,-1], color=avg_pen[2], ls='-', lw=1, label=avg_leg[2])
ax2.plot([-2,-1], [-2,-1], color='black', ls='none', marker=markers[0], mec='black', 
         mfc='black', ms=3, mew=1, label='174 Hz')
ax2.plot([-2,-1], [-2,-1], color=avg_pen[4], ls='-', lw=1, label=avg_leg[4])
ax2.plot([-2,-1], [-2,-1], color=avg_pen[6], ls='-', lw=1, label=avg_leg[6])
ax2.plot([-2,-1], [-2,-1], color='black', ls='none', marker=markers[1], mec='black',
         mfc='black', ms=3, mew=1, label='235 Hz')
#
handles, labels = ax2.get_legend_handles_labels()  # for avoiding duplicate labels
by_label = dict(zip(labels, handles))              # for avoiding duplicate labels
ax2.legend(by_label.values(), by_label.keys(), loc='upper left', ncol = 2, prop={'size':8}, framealpha=0)
ax2.axis([22, 38, -5, 2])
ax2.set_xlabel('Twist angle')
ax2.set_ylabel('$df/dT$ (cent/°C)')
ax2.set_title('Constant length')
ax2.xaxis.set_major_formatter(ticker.StrMethodFormatter(u"{x:.0f}°"))
#
#df/dT at constant tension
for string in (plot_list_A + plot_list_S):
    sdict = globals()[string]
    str_label = sdict['sleg'][0:3] if(string in plot_list_A) else sdict['sleg']
    if(string in dfdT_F_list_A):
        ax3.plot(sdict['inv_stress'], sdict['dfdT_F'], color=sdict['pen'], ls='-', lw=1, marker=sdict['mkr'], ms=3)
    ax3.plot(sdict['inv_stress'], sdict['dfdT_E'], color=sdict['pen'], ls='-', lw=1, marker=sdict['mkr'], ms=3, 
             label=str_label)
handles, labels = ax3.get_legend_handles_labels()  # for avoiding duplicate labels
by_label = dict(zip(labels, handles))              # for avoiding duplicate labels
ax3.legend(by_label.values(), by_label.keys(), loc='lower center', ncol = 3, prop={'size':8}, framealpha=0)
ax3.axis(axis_limits)
ax3.set_xlabel('1/Stress (MPa$^{-1}$)')
ax3.set_ylabel('$df/dT$ (cent/°C)')
ax3.set_title('Constant tension')
#
plt.tight_layout()
ax1.text(0.88, 0.9,'(a)', transform=ax1.transAxes)
ax2.text(0.88, 0.9,'(b)', transform=ax2.transAxes)
ax3.text(0.88, 0.9,'(c)', transform=ax3.transAxes)
#plt.savefig('./dfdT.eps', format='eps', dpi=1000)
plt.show()
```

```
T1Aa fit: offset: -0.004822,  slope: -133.497499,  rsq: 0.997447
T2Ab fit: offset:  0.159405,  slope: -108.071956,  rsq: 1.000000
T1Ba fit: offset: -0.058351,  slope: -110.117425,  rsq: 0.999942
T4Ba fit: offset: -0.074954,  slope: -126.017334,  rsq: 0.999808
T4Bb fit: offset:  0.013079,  slope: -127.217978,  rsq: 0.999631
T2Ba fit: offset: -0.186864,  slope: -124.674389,  rsq: 1.000000
T2Bb fit: offset: -0.217841,  slope: -105.977789,  rsq: 0.999944
T3Ba fit: offset: -0.027244,  slope: -119.070735,  rsq: 0.998979
T3Bb fit: offset:  0.116632,  slope: -124.426987,  rsq: 0.989357
T4Ac fit: offset: -0.196531,  slope: -122.517289,  rsq: 0.999033
T3Aa fit: offset: -0.184394,  slope: -143.146248,  rsq: 0.999195
S1Pa fit: offset: -0.157398,  slope: -139.485208,  rsq: 0.999087
S11Pa fit: offset: -0.233003,  slope: -133.987041,  rsq: 0.988897
S12Ua fit: offset: -0.187207,  slope: -125.172377,  rsq: 0.998277
S12Ub fit: offset: -0.146594,  slope: -150.934365,  rsq: 0.987919
S12Pa fit: offset: -0.041545,  slope: -133.942294,  rsq: 0.998868
S2Pa fit: offset: -0.191454,  slope: -120.356144,  rsq: 0.997499
S2Pb fit: offset: -0.338749,  slope: -124.552312,  rsq: 0.997563
S3Ua fit: offset: -0.277204,  slope: -133.853138,  rsq: 0.989387
S3Pa fit: offset: -0.160701,  slope: -149.590015,  rsq: 0.997746
S5Ua fit: offset:  0.331103,  slope:    7.926340,  rsq: 0.810084
S5Pa fit: offset:  0.211528,  slope:  -13.823246,  rsq: 0.889523
ST1b fit: offset:  0.585884,  slope:   -8.432552,  rsq: 0.376933
```

## $K\lambda = (K/F\_0)~dF/dT$ & $K\psi = - (K/\mu\_0)~d\mu/dT$ vs. 1/stress & twist angle¶

In [17]:

```
# array sequence is T1A T1B T2A T2B T3A T3B T4A T4B S11U S1P S11P S12U S12P S2P S3U S3P S5U S5P ST1
assemble_avg_arrays()
#
# K.lambda and K.psi
fig=plt.figure(figsize=(10,3.55))
ax1=fig.add_subplot(131)
ax2=fig.add_subplot(132)
ax3=fig.add_subplot(133)
#
#==================================================
# plots against 1/stress
axis_limits = [0.005, 0.047, -5, 2]
x_plot = np.arange(5.0, 300.0, 5.0)
#
ax1.plot([0,0.1],[0,0],'k--',lw=0.5)  # Zero reference
ax2.plot([0,70], [0,0],'k--',lw=0.5)  # Zero reference
ax3.plot([0,0.1],[0,0],'k--',lw=0.5)  # Zero reference
#
for string in (plot_list_A + plot_list_S):
    sdict = globals()[string]
    str_label = sdict['sleg'][0:3] if(string in plot_list_A) else sdict['sleg']
    # K.lambda = (K/F).dF/dT
    ax1.plot(sdict['inv_stress'], sdict['Klambda'], color=sdict['pen'], ls='-', lw=1, marker=sdict['mkr'], ms=3)
    # K.psi
    ax3.plot(sdict['inv_stress'], sdict['Kpsi'], color=sdict['pen'], ls='-', lw=1, marker=sdict['mkr'], ms=3, 
             label=str_label)
#
ax1.axis(axis_limits)
ax1.set_xlabel('1/Stress (MPa$^{-1}$)')
ax1.set_ylabel('$K\lambda$ (cent/°C)')
ax1.set_title('$K\lambda = K/F_0$ $dF/dT$')
#
handles, labels = ax3.get_legend_handles_labels()  # for avoiding duplicate labels
by_label = dict(zip(labels, handles))              # for avoiding duplicate labels
ax3.legend(by_label.values(), by_label.keys(), loc='lower center', ncol = 3, prop={'size':8}, framealpha=0)
ax3.axis(axis_limits)
ax3.set_xlabel('1/Stress (MPa$^{-1}$)')
ax3.set_ylabel('$K\psi$ (cent/°C)')
ax3.set_title('$K\psi = -K/ \mu_0$ $d \mu / dT$')
#
#==================================================
# plots against twist angle
axis_limits = [22, 38, -5, 2]
markers = ['s','o']
#
for index in [0,1]:
    # data from twist angle study
    for i in range(4):
        j = 2*i
        ax2.plot([avg_angle[j],avg_angle[j+1]], [avg_Klambda[j][index],avg_Klambda[j+1][index]], 
                 color=avg_pen[j], lw=1, marker=markers[index], mec=avg_pen[j], mfc=avg_pen[j], ms=3, mew=1)
    # data from surface finish study
    for j in range(8,len(avg_angle)-3):
        ax2.plot(avg_angle[j], avg_Klambda[j][index], color=avg_pen[j], marker=markers[index], 
                 mec=avg_pen[j], mfc=avg_pen[j], ms=3, mew=1)
#
# plain lines for legend
ax2.plot([-2,-1], [-2,-1], color=avg_pen[0], ls='-', lw=1, label=avg_leg[0])
ax2.plot([-2,-1], [-2,-1], color=avg_pen[2], ls='-', lw=1, label=avg_leg[2])
ax2.plot([-2,-1], [-2,-1], color='black', ls='none', marker=markers[0], mec='black', 
         mfc='black', ms=3, mew=1, label='174 Hz')
ax2.plot([-2,-1], [-2,-1], color=avg_pen[4], ls='-', lw=1, label=avg_leg[4])
ax2.plot([-2,-1], [-2,-1], color=avg_pen[6], ls='-', lw=1, label=avg_leg[6])
ax2.plot([-2,-1], [-2,-1], color='black', ls='none', marker=markers[1], mec='black',
         mfc='black', ms=3, mew=1, label='235 Hz')
#
handles, labels = ax2.get_legend_handles_labels()  # for avoiding duplicate labels
by_label = dict(zip(labels, handles))              # for avoiding duplicate labels
ax2.legend(by_label.values(), by_label.keys(), loc='upper left', ncol = 2, prop={'size':8}, framealpha=0)
ax2.axis(axis_limits)
ax2.set_xlabel('Twist angle')
ax2.set_ylabel('$K\lambda$ (cent/°C)')
ax2.set_title('$K\lambda = K/F_0$ $dF/dT$')
ax2.xaxis.set_major_formatter(ticker.StrMethodFormatter(u"{x:.0f}°"))
#
plt.tight_layout()
plt.subplots_adjust(wspace=0.26)
ax1.text(0.87, 0.9,'(a)', transform=ax1.transAxes)
ax2.text(0.87, 0.9,'(b)', transform=ax2.transAxes)
ax3.text(0.87, 0.9,'(c)', transform=ax3.transAxes)
#plt.savefig('./lambda_psi.eps', format='eps', dpi=1000)
plt.show()
```

## $(1/A\_0)\ d(AE\_T)/dT$ vs. stress & twist angle¶

In [18]:

```
# array sequence is T1A T1B T2A T2B T3A T3B T4A T4B S11U S1P S11P S12U S12P S2P S3U S3P S5U S5P ST1
assemble_avg_arrays()

Tpnts = len(plot_list_A)

fig, (ax1,ax2,ax3) = plt.subplots(1, 3, gridspec_kw={'width_ratios': [3,3,2]}, figsize=(10,3.4))

# dE/dT versus stress
for string in (plot_list_A + plot_list_S):
    sdict = globals()[string]
    str_label = sdict['sleg'][0:3] if(string in plot_list_A) else sdict['sleg']
    ax1.plot(sdict['stress'], sdict['dEtdT'], color=sdict['pen'], ls='-', lw=1, marker=sdict['mkr'], ms=4, 
             label=str_label)
handles, labels = ax1.get_legend_handles_labels()  # for avoiding duplicate labels
handles_T = handles[:Tpnts]                        # labels for twist angle study strings
handles_S = handles[Tpnts:]                        # labels for surface finish study strings
labels_T = labels[:Tpnts]
labels_S = labels[Tpnts:]
by_label_T = dict(zip(labels_T, handles_T))
by_label_S = dict(zip(labels_S, handles_S))
ax1.legend(by_label_T.values(), by_label_T.keys(), loc='lower right', ncol = 3, prop={'size':8}, framealpha=0)
ax1.axis([20, 150, -0.045, 0.0])
ax1.set_xlabel('Stress (MPa)')
ax1.set_ylabel('$1/A_0\ d(AE_T)/dT$ (GPa/°C)')
ax1.set_title('All strings')
#
# ============================================================
# dE/dT vs. twist angle
# data from twist angle study
for i in range(4):
    j = 2*i
    ax2.plot([avg_angle[j],avg_angle[j+1]], [avg_dEtdT[j][2],avg_dEtdT[j+1][2]], color=avg_pen[j], lw=1, marker='^', 
             mec=avg_pen[j], mfc=avg_pen[j], ms=3, mew=1)
    if(j == 0): # exclude T1B data point for 300 Hz
        ax2.plot([avg_angle[j]], [avg_dEtdT[j][3]], color=avg_pen[j], ls='none', marker='v', 
                 mec=avg_pen[j], mfc=avg_pen[j], ms=3, mew=1)
    else:
        ax2.plot([avg_angle[j],avg_angle[j+1]], [avg_dEtdT[j][3],avg_dEtdT[j+1][3]], color=avg_pen[j], lw=1, marker='v', 
                 mec=avg_pen[j], mfc=avg_pen[j], ms=3, mew=1)
# data from surface finish study
for j in range(8,len(avg_angle)-3):
    ax2.plot(avg_angle[j], avg_dEtdT[j][2], color=avg_pen[j], marker='^', mec=avg_pen[j], mfc=avg_pen[j], ms=3, mew=1)
    ax2.plot(avg_angle[j], avg_dEtdT[j][3], color=avg_pen[j], marker='v', mec=avg_pen[j], mfc=avg_pen[j], ms=3, mew=1)
# plain lines for legend
ax2.plot([-2,-1], [-2,-1], color=avg_pen[0], ls='-', lw=1, label=avg_leg[0])
ax2.plot([-2,-1], [-2,-1], color=avg_pen[2], ls='-', lw=1, label=avg_leg[2])
ax2.plot([-2,-1], [-2,-1], color='black', ls='none', marker='^', mec='black', 
         mfc='black', ms=3, mew=1, label='288 Hz')
ax2.plot([-2,-1], [-2,-1], color=avg_pen[4], ls='-', lw=1, label=avg_leg[4])
ax2.plot([-2,-1], [-2,-1], color=avg_pen[6], ls='-', lw=1, label=avg_leg[6])
ax2.plot([-2,-1], [-2,-1], color='black', ls='none', marker='v', mec='black',
         mfc='black', ms=3, mew=1, label='324 Hz')
# axes
legend_S = ax2.legend(by_label_S.values(), by_label_S.keys(), loc='lower center', ncol = 3, prop={'size':8}, framealpha=0)
ax2.add_artist(legend_S)
ax2.legend(loc='upper left', bbox_to_anchor=(0.08, 0, 1, 1), ncol=2, prop={'size':8}, framealpha=0)
ax2.axis([23, 37, -0.045, 0.0])
ax2.set_xlabel('Twist angle')
#ax2.set_ylabel('$1/A_0\ d(AE_T)/dT$ (GPa/°C)')
ax2.set_title('Varnished strings')
ax2.xaxis.set_major_formatter(ticker.StrMethodFormatter(u"{x:.0f}°"))
#
# ============================================================
# compare dE/dT for ground and unground strings
ax3.plot([0,0],[-0.045, 0.0],'k:',lw=1)
ax3.plot([1,1],[-0.045, 0.0],'k:',lw=1)
# array sequence is T1A T1B T2A T2B T3A T3B T4A T4B S11U S1P S11P S12U S12P S2P S3U S3P S5U S5P ST1
# indeces:           0   1   2   3   4   5   6   7   8    9   10   11   12   13  14  15  16  17  18
# 3rd point
ax3.plot([1,0], [avg_dEtdT[8][2],avg_dEtdT[10][2]], 'k--', lw=0.5)   
ax3.plot([1,0], [avg_dEtdT[11][2],avg_dEtdT[12][2]], 'k--', lw=0.5)     
ax3.plot([1,0], [avg_dEtdT[14][2],avg_dEtdT[15][2]], 'k--', lw=0.5)     
ax3.plot([1,0], [avg_dEtdT[16][2],avg_dEtdT[17][2]], 'k--', lw=0.5)      
# 4th point
ax3.plot([1,0], [avg_dEtdT[8][3],avg_dEtdT[10][3]], 'k--', lw=0.5)       
ax3.plot([1,0], [avg_dEtdT[11][3],avg_dEtdT[12][3]], 'k--', lw=0.5)       
ax3.plot([1,0], [avg_dEtdT[14][3],avg_dEtdT[15][3]], 'k--', lw=0.5)       
ax3.plot([1,0], [avg_dEtdT[16][3],avg_dEtdT[17][3]], 'k--', lw=0.5)       
#
for index in [9,10,12,13,15,17]:
    ax3.plot(0, avg_dEtdT[index][2], color=avg_pen[index], marker='^', mec=avg_pen[index], mfc=avg_pen[index], mew=1, ms=5)
    ax3.plot(0, avg_dEtdT[index][3], color=avg_pen[index], marker='v', mec=avg_pen[index], mfc=avg_pen[index], mew=1, ms=5)
for index in [8,11,14,16]:
    ax3.plot(1, avg_dEtdT[index][2], color=avg_pen[index], marker='^', mec=avg_pen[index], mfc=avg_pen[index], mew=1, ms=5)
    ax3.plot(1, avg_dEtdT[index][3], color=avg_pen[index], marker='v', mec=avg_pen[index], mfc=avg_pen[index], mew=1, ms=5)
ax3.axis([-0.2, 1.2, -0.045, 0.0])
x_axis = np.arange(2)
x_values = ['ground', 'unground']
ax3.set_xticks(x_axis, x_values)
ax3.set_xlabel('Surface finish')
#ax3.set_ylabel('$1/A_0\ d(AE_T)/dT$ (GPa/°C)')
ax3.set_title('Ground vs. unground')
#
plt.tight_layout()
plt.subplots_adjust(wspace=0.25)
ax1.text(0.88, 0.9,'(a)', transform=ax1.transAxes)
ax2.text(0.88, 0.9,'(b)', transform=ax2.transAxes)
ax3.text(0.45, 0.9,'(c)', transform=ax3.transAxes)
#plt.savefig('./dEdT.eps', format='eps', dpi=1000)
plt.show()
```

## $(1/A\_0)\ dF/dT$ vs. stress and twist angle¶

In [19]:

```
# array sequence is T1A T1B T2A T2B T3A T3B T4A T4B S11U S1P S11P S12U S12P S2P S3U S3P S5U S5P ST1
assemble_avg_arrays()

Tpnts = len(plot_list_A)

fig, (ax1,ax2,ax3) = plt.subplots(1, 3, gridspec_kw={'width_ratios': [3,3,2]}, figsize=(10,3.4))

# (1/A_0) dF/dT versus stress
for string in (plot_list_A + plot_list_S):
    sdict = globals()[string]
    str_label = sdict['sleg'][0:3] if(string in plot_list_A) else sdict['sleg']
    ax1.plot(sdict['stress'], sdict['dstressdT'], color=sdict['pen'], ls='-', lw=1, marker=sdict['mkr'], ms=4, 
             label=str_label)
handles, labels = ax1.get_legend_handles_labels()  # for avoiding duplicate labels
handles_T = handles[:Tpnts]                        # labels for twist angle study strings
handles_S = handles[Tpnts:]                        # labels for surface finish study strings
labels_T = labels[:Tpnts]
labels_S = labels[Tpnts:]
by_label_T = dict(zip(labels_T, handles_T))
by_label_S = dict(zip(labels_S, handles_S))
ax1.legend(by_label_T.values(), by_label_T.keys(), loc='lower center', ncol = 3, prop={'size':8}, framealpha=0)
ax1.axis([20, 150, -0.4, 0.0])
ax1.set_xlabel('Stress (MPa)')
ax1.set_ylabel('$1/A_0\ dF/dT$ (MPa/°C)')
ax1.set_title('All strings')
#
# ============================================================
# (1/A_0) dF/dT vs. twist angle
# data from twist angle study
for i in range(4):
    j = 2*i
    ax2.plot(avg_angle[j:j+2], avg_dstressdT[j:j+2], color=avg_pen[j], lw=1, marker='+', mec=avg_pen[j], mew=1, 
             label=avg_leg[j])
# data from surface finish study
for index in range(8,len(avg_angle)-3):
    ax2.plot(avg_angle[index], avg_dstressdT[index], color=avg_pen[index], marker='+', mec=avg_pen[index], mew=1)
# axes
legend_S = ax2.legend(by_label_S.values(), by_label_S.keys(), loc='lower center', ncol = 3, prop={'size':8}, framealpha=0)
ax2.add_artist(legend_S)
ax2.legend(loc='upper left', bbox_to_anchor=(0.08, 0, 1, 1), ncol=2, prop={'size':8}, framealpha=0)
ax2.axis([23, 37, -0.4, 0])
ax2.set_xlabel('Twist angle')
#ax2.set_ylabel('$1/A_0\ dF/dT$ (MPa/°C)')
ax2.set_title('Varnished strings')
ax2.xaxis.set_major_formatter(ticker.StrMethodFormatter(u"{x:.0f}°"))
#
# ============================================================
# compare (1/A_0) dF/dT for ground and unground strings
ax3.plot([0,0],[-0.4,0],'k:',lw=1)
ax3.plot([1,1],[-0.4,0],'k:',lw=1)
# array sequence is T1A T1B T2A T2B T3A T3B T4A T4B S11U S1P S11P S12U S12P S2P S3U S3P S5U S5P ST1
# indeces:           0   1   2   3   4   5   6   7   8    9   10   11   12   13  14  15  16  17  18
ax3.plot([1,0], [avg_dstressdT[8],avg_dstressdT[10]], 'k--', lw=0.5)       
ax3.plot([1,0], [avg_dstressdT[11],avg_dstressdT[12]], 'k--', lw=0.5)       
ax3.plot([1,0], [avg_dstressdT[14],avg_dstressdT[15]], 'k--', lw=0.5)       
ax3.plot([1,0], [avg_dstressdT[16],avg_dstressdT[17]], 'k--', lw=0.5)       
#
for index in [9,10,12,13,15,17]:
    ax3.plot(0, avg_dstressdT[index], color=avg_pen[index], marker='+', mec=avg_pen[index], mew=1.5)
for index in [8,11,14,16]:
    ax3.plot(1, avg_dstressdT[index], color=avg_pen[index], marker='+', mec=avg_pen[index], mew=1.5)
ax3.axis([-0.2, 1.2, -0.4, 0])
x_axis = np.arange(2)
x_values = ['ground', 'unground']
ax3.set_xticks(x_axis, x_values)
ax3.set_xlabel('Surface finish')
#ax3.set_ylabel('$1/A_0\ dF/dT$ (MPa/°C)')
ax3.set_title('Ground vs. unground')
#
plt.tight_layout()
plt.subplots_adjust(wspace=0.25)
ax1.text(0.88, 0.9,'(a)', transform=ax1.transAxes)
ax2.text(0.88, 0.9,'(b)', transform=ax2.transAxes)
ax3.text(0.45, 0.9,'(c)', transform=ax3.transAxes)
#plt.savefig('./dstressdT.eps', format='eps', dpi=1000)
plt.show()
```

## CLTE components¶

In [20]:

```
# array sequence is T1A T1B T2A T2B T3A T3B T4A T4B S11U S1P S11P S12U S12P S2P S3U S3P S5U S5P ST1
assemble_avg_arrays()
#
fig=plt.figure(figsize=(10,3.45))
ax1=fig.add_subplot(131)
ax2=fig.add_subplot(132)
ax3=fig.add_subplot(133)
#
#==================================================
# plots against stress
scaler = 1e6  # to give nice numbers on y axis
axis_limits = [20, 150, -300, 100]
ax1.plot([0,200],[0,0],'k--',lw=0.5)  # Zero reference
ax2.plot([0,200],[0,0],'k--',lw=0.5)  # Zero reference
ax3.plot([0,200],[0,0],'k--',lw=0.5)  # Zero reference

for string in (plot_list_A + plot_list_S):
    sdict = globals()[string]
    str_label = sdict['sleg'][0:3] if(string in plot_list_A) else sdict['sleg']
    # (F/AE^2) dE/dT
    ax1.plot(sdict['stress'], scaler*sdict['Eterm'], color=sdict['pen'], lw=1, marker=sdict['mkr'], ms=4)
    # -(1/AE) dF/dT
    ax2.plot(sdict['stress'], scaler*sdict['Fterm'], color=sdict['pen'], lw=1, marker=sdict['mkr'], ms=4,
             label=str_label)
    # -(F/AE) psi
    ax3.plot(sdict['stress'], scaler*sdict['Pterm'], color=sdict['pen'], lw=1, marker=sdict['mkr'], ms=4) 
#
ax1.axis(axis_limits)
ax1.set_xlabel('Stress (MPa)')
#ax1.set_ylabel(r'$(F_0/A_0^2 E_0^2)\ d(AE_T)/dT$ ($10^{-6}$/°C)')
ax1.set_ylabel(r'($10^{-6}$/°C)')
ax1.set_title(r'$(F_0/A_0^2 E_0^2)\ d(AE_T)/dT$')
#
handles, labels = ax2.get_legend_handles_labels()  # for avoiding duplicate labels
by_label = dict(zip(labels, handles))              # for avoiding duplicate labels
ax2.legend(by_label.values(), by_label.keys(), loc='lower center', ncol = 3, prop={'size':8}, framealpha=0)
ax2.axis(axis_limits)
ax2.set_xlabel('Stress (MPa)')
#ax2.set_ylabel(r'$-(1/A_0E_0)\ dF/dT$ ($10^{-6}$/°C)')
ax2.set_title(r'$-(1/A_0E_0)\ dF/dT$')
#
ax3.axis(axis_limits)
ax3.set_xlabel('Stress (MPa)')
#ax3.set_ylabel(r'$-(F_0/A_0 E_0)\ \psi$ ($10^{-6}$/°C)')
ax3.set_title(r'$-(F_0/A_0 E_0)\ \psi$')
#
plt.tight_layout()
plt.subplots_adjust(wspace=0.25)
ax1.text(0.86, 0.9,'(a)', transform=ax1.transAxes)
ax2.text(0.86, 0.9,'(b)', transform=ax2.transAxes)
ax3.text(0.86, 0.9,'(c)', transform=ax3.transAxes)
#plt.savefig('./CLTE_components.eps', format='eps', dpi=1000)
plt.show()
```

## CLTE vs. stress & twist angle¶

In [21]:

```
# array sequence is T1A T1B T2A T2B T3A T3B T4A T4B S11U S1P S11P S12U S12P S2P S3U S3P S5U S5P ST1
assemble_avg_arrays()

Tpnts = len(plot_list_A)

fig, (ax1,ax2,ax3) = plt.subplots(1, 3, gridspec_kw={'width_ratios': [3,3,2]}, figsize=(10,3.4))

scaler = 1e6  # to give nice numbers on y axis
sc_avg_CLTE = scaler * np.array(avg_CLTE)

# CLTE versus stress - varnished strings only
ax1.plot([0,300],[0,0],'k--',lw=0.5)  # Zero reference
for string in (plot_list_A + plot_list_SV):
    sdict = globals()[string]
    str_label = sdict['sleg'][0:3] if(string in plot_list_A) else sdict['sleg']
    ax1.plot(sdict['stress'], scaler*sdict['CLTE'], color=sdict['pen'], ls='-', lw=1, marker=sdict['mkr'], ms=4, 
             label=str_label)
handles, labels = ax1.get_legend_handles_labels()  # for avoiding duplicate labels
handles_T = handles[:Tpnts]                        # labels for twist angle study strings
handles_S = handles[Tpnts:]                        # labels for surface finish study strings
labels_T = labels[:Tpnts]
labels_S = labels[Tpnts:]
by_label_T = dict(zip(labels_T, handles_T))
by_label_S = dict(zip(labels_S, handles_S))
ax1.legend(by_label_T.values(), by_label_T.keys(), loc='lower left', ncol = 3, prop={'size':8}, framealpha=0)
ax1.axis([20, 150, -300, 50])
ax1.set_xlabel('Stress (MPa)')
ax1.set_ylabel(r'CLTE $\alpha$ ($10^{-6}$/°C)')
ax1.set_title('Varnished strings')
#
# ============================================================
# CLTE vs. twist angle - varnished strings only
markers = ['s','o','^','v']
ax2.plot([0,70],[0,0],'k--',lw=0.5)  # Zero reference
# array sequence is T1A T1B T2A T2B T3A T3B T4A T4B S11U S1P S11P S12U S12P S2P S3U S3P S5U S5P ST1
# indeces:           0   1   2   3   4   5   6   7   8    9   10   11   12   13  14  15  16  17  18
for index in [1,2]:
    # data from twist angle study
    for i in range(4):
        j = 2*i
        ax2.plot([avg_angle[j],avg_angle[j+1]], [sc_avg_CLTE[j][index],sc_avg_CLTE[j+1][index]], 
                 color=avg_pen[j], lw=1, marker=markers[index], mec=avg_pen[j], mfc=avg_pen[j], ms=3, mew=1)
        # plain lines for legend
        ax2.plot([-2,-1], [-2,-1], color=avg_pen[j], ls='-', lw=1, label=avg_leg[j])
    # data from surface finish study
    for j in range(8,len(avg_angle)-3):
        ax2.plot(avg_angle[j], sc_avg_CLTE[j][index], 
                 color=avg_pen[j], marker=markers[index], mec=avg_pen[j], mfc=avg_pen[j], ms=3, mew=1)
# axes
legend_S = ax2.legend(by_label_S.values(), by_label_S.keys(), loc='lower left', ncol = 3, prop={'size':8}, framealpha=0)
ax2.add_artist(legend_S)
handles, labels = ax2.get_legend_handles_labels()  # for avoiding duplicate labels
by_label = dict(zip(labels, handles))              # for avoiding duplicate labels
leg = ax2.legend(by_label.values(), by_label.keys(), loc='upper left', bbox_to_anchor=(0, 0, 1, 0.99), 
                 ncol=2, prop={'size':8}, framealpha=1)
leg.get_frame().set_linewidth(0.0)
ax2.axis([23, 37, -300, 50])
ax2.set_xlabel('Twist angle')
#ax2.set_ylabel(r'CLTE $\alpha$ ($10^{-6}$/°C)')
ax2.set_title('Varnished strings')
ax2.xaxis.set_major_formatter(ticker.StrMethodFormatter(u"{x:.0f}°"))
#
# ============================================================
# compare CLTE for ground and unground strings - varnished strings only
ax3.plot([-2,2],[0,0],'k--',lw=0.5)  # Zero reference
ax3.plot([0,0],[-300, 50],'k:',lw=1)
ax3.plot([1,1],[-300, 50],'k:',lw=1)
# array sequence is T1A T1B T2A T2B T3A T3B T4A T4B S11U S1P S11P S12U S12P S2P S3U S3P S5U S5P ST1
# indeces:           0   1   2   3   4   5   6   7   8    9   10   11   12   13  14  15  16  17  18
for index in [1,2]:
    ax3.plot([1,0], [sc_avg_CLTE[8][index],sc_avg_CLTE[10][index]], 'k--', lw=0.5)   
    ax3.plot([1,0], [sc_avg_CLTE[11][index],sc_avg_CLTE[12][index]], 'k--', lw=0.5)     
    ax3.plot([1,0], [sc_avg_CLTE[14][index],sc_avg_CLTE[15][index]], 'k--', lw=0.5)     
    for j in [9,10,12,13,15]:
        ax3.plot(0, sc_avg_CLTE[j][index], color=avg_pen[j], marker=markers[index], 
                 mec=avg_pen[j], mfc=avg_pen[j], mew=1, ms=5)
    for j in [8,11,14]:
        ax3.plot(1, sc_avg_CLTE[j][index], color=avg_pen[j], marker=markers[index], 
                 mec=avg_pen[j], mfc=avg_pen[j], mew=1, ms=5)
# plain lines for legend
ax3.plot([-2,-1], [-2,-1], color='black', ls='none', marker=markers[1], mec='black',
         mfc='black', ms=5, mew=1, label='235 Hz')
ax3.plot([-2,-1], [-2,-1], color='black', ls='none', marker=markers[2], mec='black',
         mfc='black', ms=5, mew=1, label='288 Hz')
ax3.legend(loc='lower center', ncol=1, prop={'size':8}, bbox_to_anchor=(0, 0.1, 1, 1), framealpha=0)
ax3.axis([-0.2, 1.2, -300, 50])
x_axis = np.arange(2)
x_values = ['ground', 'unground']
ax3.set_xticks(x_axis, x_values)
ax3.set_xlabel('Surface finish')
#ax3.set_ylabel(r'CLTE $\alpha$ ($10^{-6}$/°C)')
ax3.set_title('Ground vs. unground')
#
plt.tight_layout()
plt.subplots_adjust(wspace=0.25)
ax1.text(0.88, 0.9,'(a)', transform=ax1.transAxes)
ax2.text(0.88, 0.9,'(b)', transform=ax2.transAxes)
ax3.text(0.45, 0.9,'(c)', transform=ax3.transAxes)
#plt.savefig('./CLTE.eps', format='eps', dpi=1000)
plt.show()
```

In [ ]:

```

```
