# Supplementary material for: Effects of Twisting and Surface Finish on the Mechanical Properties of Natural Gut Harp Strings
Source: Materials (Basel). 2023 Aug 3;16(15):5444. doi: 10.3390/ma16155444 (PMC10419811; doi:10.3390/ma16155444)
Supplement: Supplementary file 1 [file materials-16-05444-s001.zip › Surface finish humidity tests plots for paper.html]

Surface finish humidity tests plots for paper


# Figures for humidity tests with gut strings¶

In [1]:

```
from pathlib import Path
import math
import numpy as np
import numpy.polynomial.polynomial as poly
import matplotlib.pyplot as plt
import matplotlib.ticker as ticker
#
# enable plots to be shown in cells
%matplotlib inline

from datetime import datetime
FMT_time = '%H:%M:%S'
FMT_date = '%Y/%m/%d'

def read_data_file(filename, time_array, frequency_array, tension_array, temperature_array, RH_array, AH_array):
    # basic version, excludes points where fundamental frequency not identified
    # words[2] always set to 'ok' during constant-length (no adjustment) main test run
    global start_date
    global start_time
    f = open(filename, "r")
    # read in data
    data = f.readlines()
    found_start = False
    start_tension = 0.0
    for line in data:
        words = line.split(',')
        if('No_Adjustment_mode' in words[0]):
            found_start = True
        elif((len(words) > 9) and (words[2] == 'ok') and (words[3] == 'ok')):   # data record with fundamental identified OK
            if(found_start == False):   # pre-tuning phase
                start_tension = float(words[5])  # ends up holding the last tension reading from the pre-tuning phase
            else:   # found_start == True, main test run
                line_date = datetime.strptime(words[0],FMT_date)
                line_time = datetime.strptime(words[1],FMT_time)
                if(len(time_array)==0):
                    start_date = line_date
                    start_time = line_time
                    days = 0.0
                else:
                    date_diff = line_date - start_date
                    time_diff = line_time - start_time # could be negative
                    days = date_diff.days + time_diff.days + time_diff.seconds / (24.0 * 3600.0)
                time_array.append(days)
                frequency_array.append(float(words[4]))
                tension_array.append(float(words[5]))
                temperature_array.append(float(words[7]))
                RH_array.append(float(words[8]))
                AH_array.append(float(words[9]))
    # close file
    f.close()
    return start_tension

def read_data_file0(filename, time_array, frequency_array, tension_array, temperature_array, RH_array, AH_array):
    # modified version, includes points where fundamental frequency not identified
    # words[2] always set to 'ok' during constant-length (no adjustment) main test run
    global start_date
    global start_time
    f = open(filename, "r")
    # read in data
    data = f.readlines()
    found_start = False
    start_tension = 0.0
    for line in data:
        words = line.split(',')
        if('No_Adjustment_mode' in words[0]):
            found_start = True
        elif((len(words) > 9) and (words[2] == 'ok')):   # data record
            if(found_start == False):   # pre-tuning phase
                start_tension = float(words[5])  # ends up holding the last tension reading from the pre-tuning phase
            else:   # found_start == True, main test run
                line_date = datetime.strptime(words[0],FMT_date)
                line_time = datetime.strptime(words[1],FMT_time)
                if(len(time_array)==0):
                    start_date = line_date
                    start_time = line_time
                    days = 0.0
                else:
                    date_diff = line_date - start_date
                    time_diff = line_time - start_time # could be negative
                    days = date_diff.days + time_diff.days + time_diff.seconds / (24.0 * 3600.0)
                time_array.append(days)
                frequency_array.append(float(words[4]))
                tension_array.append(float(words[5]))
                temperature_array.append(float(words[7]))
                RH_array.append(float(words[8]))
                AH_array.append(float(words[9]))
    # close file
    f.close()
    return start_tension

def derived_responses(freq_array, tension_array, target_freq, freq_dev_array, vibr_length, mu_array): # vibr_length in m
    four_Lv_sq = 4.0 * vibr_length * vibr_length
    for i in range(0, len(freq_array), 1):
        freq_dev_array.append(1200.0 * math.log(freq_array[i] / target_freq, 2.0))
        mu_array.append(1000.0 * tension_array[i] / (four_Lv_sq * freq_array[i] * freq_array[i])) # g/m

def locate_time_index(time_array, search_time, start_index): 
    # returns 0 if end of time_array reached before search_time found
    finished = False
    found_it = False
    scan_index = start_index - 1
    while((found_it == False) and (finished == False)):
        scan_index += 1;
        if(scan_index >= len(time_array)):
            finished = True
        elif(time_array[scan_index] >= search_time):
            found_it = True
    if(found_it == True):
        return scan_index
    else:
        return 0
```

## Read and plot creep response data¶

In [2]:

```
time_margin = 0.3
T_margin = 0.5
RH_margin = 1
mu_margin = 0.005
F_margin = 1
fdev_margin = 5

save_format = 'eps' #'png'
fn_start = 'sf_'

S2Pa =  {'rig':2,'target':324}  
S5Pa =  {'rig':2,'target':324}  

# string specific parameters
# sdict['cr_times'] gives start and stop times for creep periods to have lines fitted; use -1 to specify end of response
# S2Pa, start time = 26/03/2022 19:00:36 GMT
S2Pa['filelist'] = ["string_test_data_2022.03.26.17.34.csv"]
S2Pa['filecopy'] = False
S2Pa['rt_index'] = -1
S2Pa['xtick_spacing'] = 1
S2Pa['ytick_spacing_t'] = 1
S2Pa['ytick_spacing_f'] = 2
S2Pa['water_add'] = [1.735,14.85]
S2Pa['transition'] = [3.2,7.92,18.2,23.5]  # 16.7?
S2Pa['temp_exc'] = []
S2Pa['cr_times'] = [S2Pa['water_add'][0],S2Pa['transition'][0],S2Pa['transition'][0],S2Pa['transition'][1],
                    S2Pa['water_add'][1],S2Pa['transition'][3]]  
# S5Pa, start time = 24/10/2022 19:39:15 GMT
S5Pa['filelist'] = ["string_test_data_2022.10.24.17.53.csv"]
S5Pa['filecopy'] = False
S5Pa['rt_index'] = -1
S5Pa['xtick_spacing'] = 1
S5Pa['ytick_spacing_t'] = 1
S5Pa['ytick_spacing_f'] = 2
S5Pa['water_add'] = [2.5109]
S5Pa['transition'] = [11.1] 
S5Pa['temp_exc'] = []
S5Pa['cr_times'] = []  

for string in ['S2Pa','S5Pa']:
    sdict = globals()[string]

    if(sdict['rig'] == 1):
        sdict['L_total'] = 0.555
        sdict['L_v'] = 0.5
    else: # rig 2
        sdict['L_total'] = 0.562
        sdict['L_v'] = 0.4985
        
    print('\nSTRING %s at %d Hz on test rig %d\n' % (string, sdict['target'], sdict['rig']))

    # load data set
    sdict['time'] = []
    sdict['freq'] = []
    sdict['tens'] = []
    sdict['temp'] = []
    sdict['RH'] = []
    sdict['AH'] = []
    for filename in sdict['filelist']:
        d_filename = "./" + filename
        if (string in ['S2Pa']):
            read_data_file0(d_filename, sdict['time'], sdict['freq'], sdict['tens'], sdict['temp'], sdict['RH'], sdict['AH'])
        else:
            read_data_file(d_filename, sdict['time'], sdict['freq'], sdict['tens'], sdict['temp'], sdict['RH'], sdict['AH'])
    if(len(sdict['time']) <= 0):
        print("\n*** NO DATA ***\n")
    else:
        # derived responses
        sdict['freq_dev'] = []
        sdict['mu'] = []
        derived_responses(sdict['freq'], sdict['tens'], sdict['target'], sdict['freq_dev'], sdict['L_v'], sdict['mu'])
        run_time = sdict['time'][sdict['rt_index']]

        # plot main response set
        fig=plt.figure(figsize=(8,6))
        ax1=fig.add_subplot(411)
        ax2=fig.add_subplot(412)
        ax3=fig.add_subplot(413)
        ax4=fig.add_subplot(414)
        # y axis ranges
        y1_min = np.min(sdict['RH']) - RH_margin
        y1_max = np.max(sdict['RH']) + RH_margin
        y2_min = np.min(sdict['mu']) - mu_margin
        y2_max = np.max(sdict['mu']) + mu_margin
        y3_min = np.min(sdict['tens']) - F_margin
        y3_max = np.max(sdict['tens']) + F_margin
        y4_min = np.min(sdict['freq_dev']) - fdev_margin
        y4_max = np.max(sdict['freq_dev']) + fdev_margin
        # plot data
        ax1.plot(sdict['time'], sdict['RH'], 'b', lw=1)
        ax2.plot(sdict['time'], sdict['mu'], 'b', lw=1)
        ax3.plot(sdict['time'], sdict['tens'], 'b', lw=1)
        ax4.plot(sdict['time'], sdict['freq_dev'], 'b', lw=1)
        # water addition markers
        for i in range(len(sdict['water_add'])):
            ax1.plot([sdict['water_add'][i],sdict['water_add'][i]],[y1_min,y1_max], 'r--', lw=1)
            ax2.plot([sdict['water_add'][i],sdict['water_add'][i]],[y2_min,y2_max], 'r--', lw=1)
            ax3.plot([sdict['water_add'][i],sdict['water_add'][i]],[y3_min,y3_max], 'r--', lw=1)
            ax4.plot([sdict['water_add'][i],sdict['water_add'][i]],[y4_min,y4_max], 'r--', lw=1)
        # transition markers
        for i in range(len(sdict['transition'])):
            ax1.plot([sdict['transition'][i],sdict['transition'][i]],[y1_min,y1_max], 'k--', lw=1)
            ax2.plot([sdict['transition'][i],sdict['transition'][i]],[y2_min,y2_max], 'k--', lw=1)
            ax3.plot([sdict['transition'][i],sdict['transition'][i]],[y3_min,y3_max], 'k--', lw=1)
            ax4.plot([sdict['transition'][i],sdict['transition'][i]],[y4_min,y4_max], 'k--', lw=1)
        # creep rate slopes
        sections = int(np.round(len(sdict['cr_times']) / 2))
        sdict['cr_rates'] = np.zeros(sections)
        for i in range(sections):
            start_time = sdict['cr_times'][2*i]
            stop_time = sdict['cr_times'][2*i+1] if(sdict['cr_times'][2*i+1] > 0) else (sdict['time'][-1] - time_margin)
            index1 = locate_time_index(sdict['time'], start_time, 0)
            index2 = locate_time_index(sdict['time'], stop_time, 0)
            x1 = start_time - time_margin
            x2 = stop_time + time_margin
            if((string == 'S1Pa') and (i==0)): # first fit for string S1Pa
                cut_start = 2.7
                cut_stop = 4.0
                cut_start_index = locate_time_index(sdict['time'], cut_start, 0)
                cut_stop_index = locate_time_index(sdict['time'], cut_stop, 0)
                time_piece = np.concatenate((sdict['time'][index1:cut_start_index],sdict['time'][cut_stop_index:index2]))
                freq_dev_piece = np.concatenate((sdict['freq_dev'][index1:cut_start_index],sdict['freq_dev'][cut_stop_index:index2]))
                cr_fit = poly.polyfit(time_piece, freq_dev_piece, 1)  # note coefficients now in increasing order
            else:
                cr_fit = poly.polyfit(sdict['time'][index1:index2], sdict['freq_dev'][index1:index2], 1)  # note coefficients now in increasing order
            sdict['cr_rates'][i] = cr_fit[1]
            print("fit %d:  offset: %0.6f,  slope: %0.6f cent/day" % (i+1, cr_fit[0], cr_fit[1]))
            ax4.plot([x1,x2], poly.polyval([x1,x2], cr_fit), 'k--', lw=1)
        # labels
        if (string == 'S2Pa'):
            ax4.text(2.8, 0, "%.1f cent/day" %(sdict['cr_rates'][0]), backgroundcolor='1')
            ax4.text(5.5, -30, "%.1f cent/day" %(sdict['cr_rates'][1]), backgroundcolor='1')
            ax4.text(18, -70, "%.1f cent/day" %(sdict['cr_rates'][2]), backgroundcolor='1')
        #
        # axes and titles
        ax1.axis([-0.2,run_time+0.2,y1_min,y1_max])
        ax2.axis([-0.2,run_time+0.2,y2_min,y2_max])
        ax3.axis([-0.2,run_time+0.2,y3_min,y3_max])
        ax4.axis([-0.2,run_time+0.2,y4_min,y4_max])
        ax1.xaxis.set_major_locator(ticker.MultipleLocator(sdict['xtick_spacing']))
        ax2.xaxis.set_major_locator(ticker.MultipleLocator(sdict['xtick_spacing']))
        ax3.xaxis.set_major_locator(ticker.MultipleLocator(sdict['xtick_spacing']))
        ax4.xaxis.set_major_locator(ticker.MultipleLocator(sdict['xtick_spacing']))
        ax1.tick_params(axis='x',which='both',bottom=False,top=False,labelbottom=False)
        ax2.tick_params(axis='x',which='both',bottom=False,top=False,labelbottom=False)
        ax3.tick_params(axis='x',which='both',bottom=False,top=False,labelbottom=False)
        ax4.set_xlabel('Time (days)')
        ax1.set_ylabel('RH (%)')
        ax2.set_ylabel('$\mu$ (g/m)')
        ax3.set_ylabel('Tension (N)')
        ax4.set_ylabel('Freq. dev. (cent)')
        fig.align_ylabels([ax1,ax2,ax3,ax4])
        title_str = string + ' at ' + str(sdict['target']) + ' Hz (Rig ' + str(sdict['rig']) + ')'
        ax1.set_title(title_str)
        #
        fig.tight_layout()  
        plt.subplots_adjust(hspace=0)
        ax1.text(0.96, 0.85,'(a)', transform=ax1.transAxes)
        ax2.text(0.96, 0.85,'(b)', transform=ax2.transAxes)
        ax3.text(0.96, 0.85,'(c)', transform=ax3.transAxes)
        ax4.text(0.96, 0.85,'(d)', transform=ax4.transAxes)
        s_format = save_format
        savefile = './' + fn_start + string + '_humidity.' + s_format
        print('\n',savefile)
#        plt.savefig(savefile, format=s_format, dpi=1000)
        plt.show()
```

```
STRING S2Pa at 324 Hz on test rig 2

fit 1:  offset: 5.410310,  slope: -6.418452 cent/day
fit 2:  offset: 18.099180,  slope: -11.118516 cent/day
fit 3:  offset: 9.543806,  slope: -5.121702 cent/day

 ./sf_S2Pa_humidity.eps
```

```
STRING S5Pa at 324 Hz on test rig 2


 ./sf_S5Pa_humidity.eps
```

In [ ]:

```

```
